# Glibenclamide targets MDH2 to relieve aging phenotypes through metabolism-regulated epigenetic modification

**DOI:** 10.1038/s41392-025-02157-3

**Published:** 2025-02-17

**Authors:** Zhifan Mao, Wenwen Liu, Rong Zou, Ling Sun, Shuman Huang, Lingyu Wu, Liru Chen, Jiale Wu, Shijie Lu, Zhouzhi Song, Xie Li, Yunyuan Huang, Yong Rao, Yi-You Huang, Baoli Li, Zelan Hu, Jian Li

**Affiliations:** 1 State Key Laboratory of Bioreactor Engineering, Shanghai Frontiers Science Center of Optogenetic Techniques for Cell Metabolism, Frontiers Science Center for Materialbiology and Dynamic Chemistry, Shanghai Key Laboratory of New Drug Design, School of Pharmacy, East China University of Science and Technology, Shanghai, 200237 China; 2 Key Laboratory of Tropical Biological Resources of Ministry of Education and Hainan, Engineering Research Center for Drug Screening and Evaluation, School of Pharmaceutical Sciences, Hainan University, Haikou, 570228 China; 3 Hubei Key Laboratory of Genetic Regulation and Integrative Biology, School of Life Sciences, Central China Normal University, Wuhan, China; 4 Key Laboratory of Xinjiang Phytomedicine Resource and Utilization, Ministry of Education, School of Pharmacy, Shihezi University, Shihezi, 832003 China

**Keywords:** Drug discovery, Chemical biology

## Abstract

Mitochondrial metabolism-regulated epigenetic modification is a driving force of aging and a promising target for therapeutic intervention. Mitochondrial malate dehydrogenase (MDH2), an enzyme in the TCA cycle, was identified as an anti-aging target through activity-based protein profiling in present study. The expression level of MDH2 was positively correlated with the cellular senescence in *Mdh2* knockdown or overexpression fibroblasts. Glibenclamide (Gli), a classic anti-glycemic drug, was found to inhibit the activity of MDH2 and relieve fibroblast senescence in an MDH2-dependent manner. The anti-aging effects of Gli were also further validated in vivo, as it extended the lifespan and reduced the frailty index of naturally aged mice. Liver specific *Mdh2* knockdown eliminated Gli’s beneficial effects in naturally aged mice, reducing p16^INK4a^ expression and hepatic fibrosis. Mechanistically, MDH2 inhibition or knockdown disrupted central carbon metabolism, then enhanced the methionine cycle flux, and subsequently promoted histone methylation. Notably, the tri-methylation of H3K27, identified as a crucial methylation site in reversing cellular senescence, was significantly elevated in hepatic tissues of naturally aged mice with *Mdh2* knockdown. Taken together, these findings reveal that MDH2 inhibition or knockdown delays the aging process through metabolic-epigenetic regulation. Our research not only identified MDH2 as a potential therapeutic target and Gli as a lead compound for anti-aging drug development, but also shed light on the intricate interplay of metabolism and epigenetic modifications in aging.

## Introduction

Aging, accompanied with internal accumulation of senescent cells, leads to various age-related pathologies, including frailty, cancer, and multiple organ dysfunctions.^1^ Drug interventions that reverse the aging process can delay the onset of age-related diseases, offering great social and economic benefits.^2,3^ However, a major challenge in the development of anti-aging drugs is the identification of effective targets.

Epigenetic dysregulation has emerged as a critical hallmark and driving force of aging,^4^ suggesting that ameliorating this dysregulation could be a promising strategy for relieving aging phenotypes.^5^ Alteration in histone modifications is a form of epigenetic modifications, that plays a crucial role in DNA packaging, and thus regulates the expression of age-related genes.^6^ For instance, H3K4me3 and H3K27me3 act as activation and suppression marks, respectively, to mediate the expression of *Cdkn1a* (*p21*^*WAF1/Cip1*^)^7,8^ and *Cdkn2a* (*p16*^*INK4a*^),^9,10^ which are cyclin-dependent kinase inhibitors upregulated in senescent cells. Defects in H3K9me3 cause repetitive elements triggered inflammatory response,^11,12^ which is both a hallmark and driving force of cellular senescence and tissue aging. Thus, amenable targets for pharmacological intervention through epigenetic modifications hold great potential in relieving aging phenotypes.

Though interventions of age-related histone methylation changes hold therapeutical benefits for relieving aging phenotypes,^13^ few small molecules precisely targeting transmethylase and demethylase have been developed for rejuvenations, likely due to the structural similarity of these enzymes.^11^ Consequently, the metabolic-epigenetic circuit presents an alternative strategy to regulate histone methylation through metabolic intervention.^14,15^ Metabolic process provides substrates and cofactors for epigenetic modifications, which constitutes the metabolic-epigenetic circuitry.^16,17^ Supplementation of metabolites, including NAD^+^ precursors,^18,19^ α-ketoglutarates,^20,21^ taurine,^22^ and uridine^23^ has been reported to exhibit rejuvenating effects partly through epigenetic regulation. However, to the best of our knowledge, whether targeting enzymes in metabolic process by small molecule compounds is capable to relieve the aging phenotypes through metabolic-epigenetic regulation remains largely unknown.

Our previous studies discovered that chlorpropamide (Chl), a compound of the sulfonylurea class, exerts anti-aging effects on *Caenorhabditis elegans* through mitochondrial related pathways.^24^ However, the anti-aging target of Chl and compounds in sulfonylurea class remains to be elucidated. In this study, mitochondrial malate dehydrogenase (MDH2) was identified as a potential target for aging intervention utilizing a chemical probe designed based on Chl. We found that knockdown of *Mdh2* delayed cellular senescence, while overexpression of *Mdh2* aggravated cellular senescence in mammalian fibroblasts. Furthermore, we demonstrated that glibenclamide (Gli), which exhibited the most potent MDH2 inhibition activity among listed sulfonylureas, delayed the aging process both in vitro and in vivo in an MDH2-dependent manner. Additionally, it was found that both endogenous metabolites and histone methylation regulated by Gli were dependent on MDH2, which suggests that knockdown of *Mdh2* or inhibition of MDH2 activity remodel the histone modifications through metabolic regulation, and thus relieve cellular senescence and mice aging. These findings provide an amenable target within the TCA cycle for aging intervention, and highlight that inhibiting the activity of enzymes involved in energy metabolism can potentially reverse age-related epigenetic alterations through metabolic regulation.

## Results

### MDH2 regulates cellular senescence

**Fig. 1:**
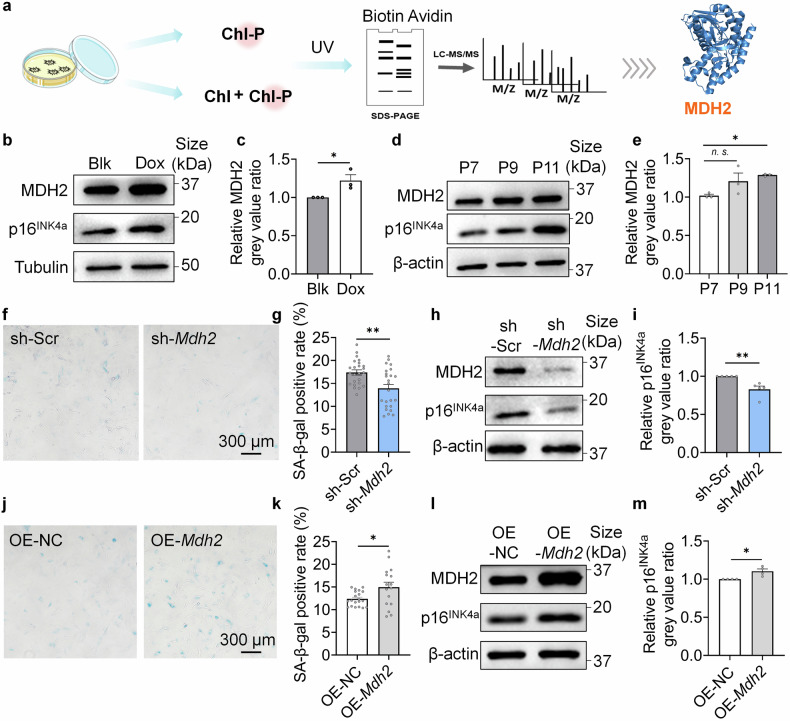
MDH2 regulates cellular senescence. **a** Schematic diagram of ABPP. **b** Relative MDH2 and p16^INK4a^ level in Blk and Dox-induced senescent MRC-5 cells. **c** Quantification of **b**. **d** Relative MDH2 and p16^INK4a^ level in replicative senescent MEFs form P7 to P11. **e** Quantification of **d**. **f** SA-β-gal staining of sh-Scr/sh-*Mdh2* MEFs (P11) transfected at P4. **g** Quantification of **f**. **h** Relative MDH2 and p16^INK4a^ level in sh-Scr/sh-*Mdh2* MEFs (P7) transfected at P4. **i** Quantification of **h**. **j** SA-β-gal staining of OE-NC/OE-*Mdh2* MEFs (P10) transfected at P4. **k** Quantification of **j**. **l** Relative MDH2 and p16^INK4a^ level in OE-NC/OE-*Mdh2* MEFs (P8) transfected at P4. **m** Quantification of **l**. Error bars represent the standard deviation (± SEM.). The significance of differences of **c,** **g,** **i,** **k,** **m** was analyzed with two-sided Student’s *t*-test. The significance of difference of **e** was analyzed with Dunnett’s multiple comparisons tests (*p < 0.05, **p < 0.01, ***p < 0.005, *n. s.*, not significant)

The focus of the work is discovering therapeutic target proteins for aging intervention. Using a chemical probe (Chl-P) designed based on our previously reported anti-aging compound (Chl) targeting mitochondria (Supplementary Data 1 Scheme 1),^24^ we performed activity-based protein profiling (ABPP) to discover potential anti-aging targets of sulfonylureas (Fig. 1a). Among the proteins detected through in situ Chl-P labeling and competition of 10-fold Chl in human embryonic lung fibroblasts (MRC-5 cells) (Supplementary Fig. 1a), MDH2 emerged as a potential target (Supplementary Fig. 1b, Fig. 1a), because its functions in mitochondrial metabolism are related to the anti-aging mechanism of sulfonylureas that we previously reported.^24^

We conducted immunoblotting to measure MDH2 level in doxorubicin (Dox)-induced senescent MRC-5 cells (Fig. 1b, c) and replicative senescent mice embryonic fibroblasts (MEFs) (Fig. 1d, e). The results showed a positive correlation between MDH2 level and cellular senescence (Fig. 1b–e). Notably, increased MDH2 expression preceded the upregulation of p16^INK4a^ as passage times increased (Fig. 1d), suggesting that the elevated MDH2 may contribute to cellular senescence in vitro. MEFs with either knockdown (sh-*Mdh2*) or overexpression (OE-*Mdh2*) of MDH2 were then constructed. Biomarkers of cellular senescence including SA-β-gal and p16^INK4a^ were measured in both cell lines. Consistent with the increased MDH2 level in senescent fibroblasts, sh-*Mdh2* MEFs exhibited lower SA-β-gal positivity (Fig. 1f, g) and reduced p16^INK4a^ expression (Fig. 1h, i), while OE-*Mdh2* MEFs showed higher SA-β-gal positivity (Fig. 1j, k) and increased p16^INK4a^ expression (Fig. 1l, m). These results confirmed that MDH2 regulates cellular senescent process of fibroblasts cultured in vitro.

### Gli inhibits MDH2 and delays cellular senescence and mice aging

**Fig. 2:**
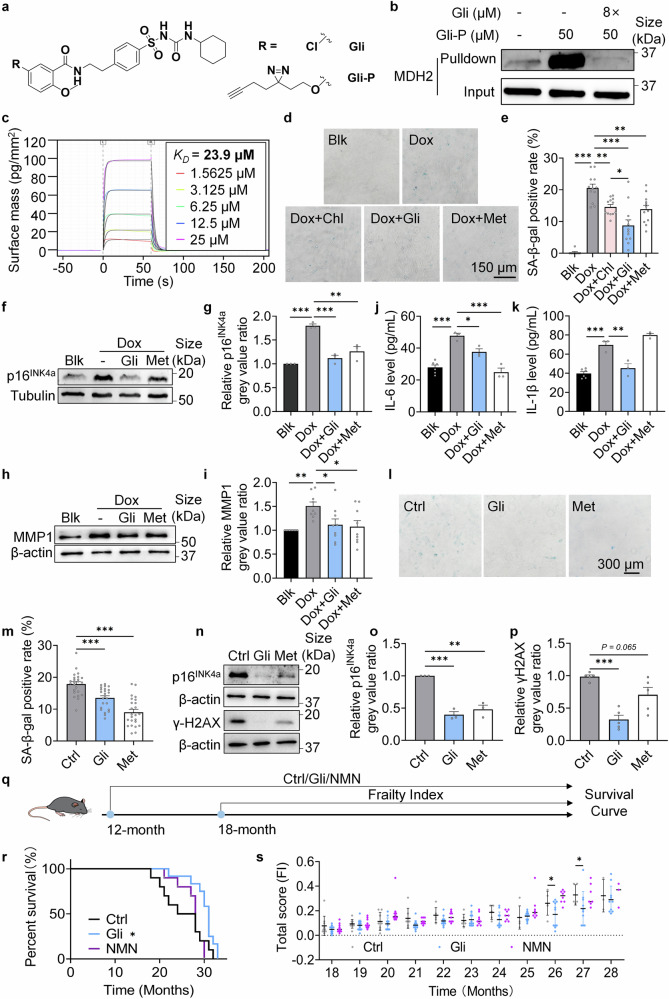
Gli relieves aging phenotypes in vitro and in vivo. **a** Structure of Gli and the chemical probe of Gli (Gli-P). **b** MDH2 immunoblotting of proteins pulldown from in situ labeling of MRC-5 cells using Gli-P. **c** Binding constant (*K*_*D*_) of Gli to MDH2 measured by Grating-Coupled Interferometry. **d** SA-β-gal staining of Blk and Dox-induced senescent MRC-5 cells treated with -/Chl (200 μM)/Gli (100 μM)/Met (100 μM) for 7 days. **e** Quantification of **d**. **f** Relative p16^INK4a^ level in Blk and Dox-induced senescent MRC-5 cells treated with -/Gli (100 μM)/Met (100 μM) for 7 days. **g** Quantification of **f**. **h** Relative MMP1 level in Blk and Dox-induced senescent MRC-5 cells treated with -/Gli (100 μM)/Met (100 μM) for 7 days. **i** Quantification of **h**. **j** IL-6 level in the medium of Blk and Dox-induced senescent MRC-5 cells treated with -/Gli (100 μM)/Met (100 μM) for 7 days. **k** IL-1β level in the medium of Blk and Dox-induced senescent MRC-5 cells treated with -/Gli (100 μM)/Met (100 μM) for 7 days. **l** SA-β-gal staining of MEFs (P6) treated with -/Gli (100 μM)/Met (100 μM) for 15 days. **m** Quantification of **l**. **n** Relative p16^INK4a^ and γH2AX level in MEFs (P6) treated with -/Gli (100 μM)/Met (100 μM) for 5 days. **o** Quantification of p16^INK4a^ in **n**. **p** Quantification of γH2AX in **n**. **q** Diagram of assays on naturally aged mice treated with -/Gli (10 mg/kg)/NMN (500 mg/kg) daily. **r** Lifespan curves of mice in different groups (Ctrl, *n* = 10; Gli, *n* = 12; NMN, *n* = 10). **s** Frailty index of mice in different groups (Ctrl, *n* = 10; Gli, *n* = 12; NMN, *n* = 10 when the test began at 18-month age). For **s**, every dot in the plot presents the data of 1 mouse. Error bars represent the standard deviation (± SEM.). Log-rank tests were used for analyzing the significant differences of **r**. Other differences were analyzed with Tukey’s multiple comparations tests (*p < 0.05, **p < 0.01, ***p < 0.005, *n*. *s*., not significant)

To test the effects of sulfonylureas on MDH2, we evaluated the activity of recombinant MDH2 when treated with various sulfonylureas including Chl, Gli, tolbutamine (Tolb), gliclazide (Glic), and glipizide (Glip). Among these, Gli exhibited the highest inhibition rate of MDH2, whereas Chl only showed a weak inhibitory effect (Supplementary Fig. 1c). To verify the interaction between Gli and MDH2, a chemical probe (Gli-P) was synthesized (Fig. 2a, Supplementary Data 1 Scheme 2). In situ labeling of MDH2 in MRC-5 cells with Gli-P was significantly competed by 8-fold Gli (Fig. 2b), indicating a direct interaction between Gli and MDH2. The binding constant (*K*_*D*_) of Gli to MDH2 was determined to be 23.9 μM (Fig. 2c), which confirms the interaction between Gli and MDH2. Accordingly, Gli showed better effect in relieving senescent-associated β-galactosidase (SA-β-gal) staining in Dox-induced senescent cells than Chl (Fig. 2d, e), supporting that the inhibitory rate of MDH2 activity may determine the strength of the effects on relieving cellular senescence. Metformin (Met) was used as a positive control showing that the senescent process of cells was delayable.

To further confirm the effect of Gli on delaying cellular senescence, we examined the level of p16^INK4a^, a biomarker of cell cycle arrest, in Dox-induced senescent MRC-5 cells. As expected, p16^INK4a^ level was significantly reduced in Gli-treated cells (Fig. 2f, g). The senescence-associated secretory phenotype (SASP), which includes cytokines, chemokines, growth factors, and matrix metalloproteinases is produced by senescent cells and drives secondary senescence of peripheral cells and chronic inflammation.^25–27^ We measured matrix metalloproteinase 1 (MMP1) level in Dox-induced senescent MRC-5 cells and found a reduction in Gli-treated groups (Fig. 2h, i). Further investigation into the SASP inhibitory effect of Gli revealed decreased secretion of interleukin 6 (IL-6) (Fig. 2j) and interleukin 1β (IL-1β) (Fig. 2k) in the cultured medium of MRC-5 cells. Additionally, Gli treatment reduced the SA-β-gal positivity (Fig. 2l, m), p16^INK4a^ level (Fig. 2n, o), and γH2AX level (Fig. 2n, p) in replicative senescent MEFs, indicating that Gli delays both Dox-induced cellular senescence and replicative senescence, and relieves the DNA damage accumulation accompanied with the cellular senescence process.

To validate the anti-aging effects of Gli in vivo, 12-month-old mice were administered 10 mg/kg Gli, 500 mg/kg NMN or a solvent control daily until death (Fig. 2q). After 17 months of treatment (29-month-age), Gli-treated mice displayed better fur color and coat condition (Supplementary Fig. 2). Besides, Gli treatment significantly extended the lifespan of naturally aged mice (Fig. 2r), and reduced the total frailty score of mice in their late life (26 to 27 months old) (Fig. 2s). These results indicates that Gli prolonged both lifespan and healthspan of naturally aged mice, confirming that Gli, an MDH2 inhibitor, exerts anti-aging effects in vivo.

### Gli alleviates cellular senescence dependent on MDH2

**Fig. 3:**
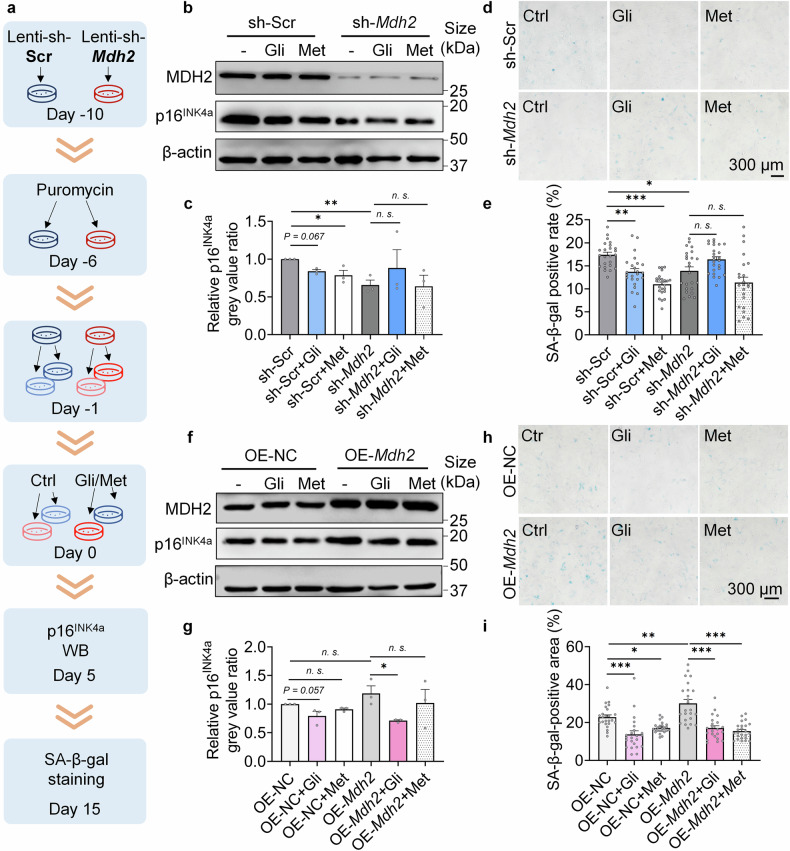
Gli alleviates cellular senescence dependent on MDH2. **a** Diagram of the MDH2 knockdown or overexpression assay in cells. **b** Relative p16^INK4a^ level in sh-Scr/sh-*Mdh2* MEFs (P7) treated with -/Gli (100 μM)/Met (100 μM) for 5 days. **c** Quantification of **b**. **d** SA-β-gal staining of sh-Scr/sh-*Mdh2* MEFs (P7) treated with -/Gli (100 μM)/Met (100 μM) for 15 days. **e** Quantification of **d**. **f** Relative p16^INK4a^ level in OE-NC/OE-*Mdh2* MEFs (P7) treated with -/Gli (100 μM)/Met (100 μM) for 5 days. **g** Quantification of **f**. **h** SA-β-gal staining of OE-NC/OE-*Mdh2* MEFs (P7) treated with -/Gli (100 μM)/Met (100 μM) for 15 days. **i** Quantification of **h**. Error bars represent the standard deviation (± SEM.). The significance of differences (*p < 0.05, **p < 0.01, ***p < 0.005, n. s., not significant) of all panels were analyzed with Tukey’s multiple comparations tests

To determine whether the effects of Gli in relieving cellular senescence are dependent on MDH2, we created sh-*Mdh2* MRC-5 cells (Supplementary Fig. 3a) and sh-*Mdh2* MEFs (Fig. 3a), and assessed the biomarkers of cellular senescence on both cell lines. In replicative senescent MRC-5 cells, sh-*Mdh2* significantly reduced SA-β-gal positivity and nullified the effects of Chl and Gli, while Gli relieved more SA-β-gal staining than Chl (Supplementary Fig. 3b, c). Similarly, in MEFs, the effects of Gli on p16^INK4a^ expression (Fig. 3b, c) and SA-β-gal positivity (Fig. 3d, e) were dependent on MDH2. We also tested the effects of Gli on OE-*Mdh2* MEFs, and found that Gli reduced p16^INK4a^ expression (Fig. 3f, g) and SA-β-gal positivity (Fig. 3h, i) in OE-*Mdh2* MEFs to the same extent as in OE-NC (negative control) MEFs. These results demonstrate that Gli delays cellular senescence in an MDH2-dependent manner.

### Gli regulates central carbon metabolism through inhibiting MDH2

**Fig. 4:**
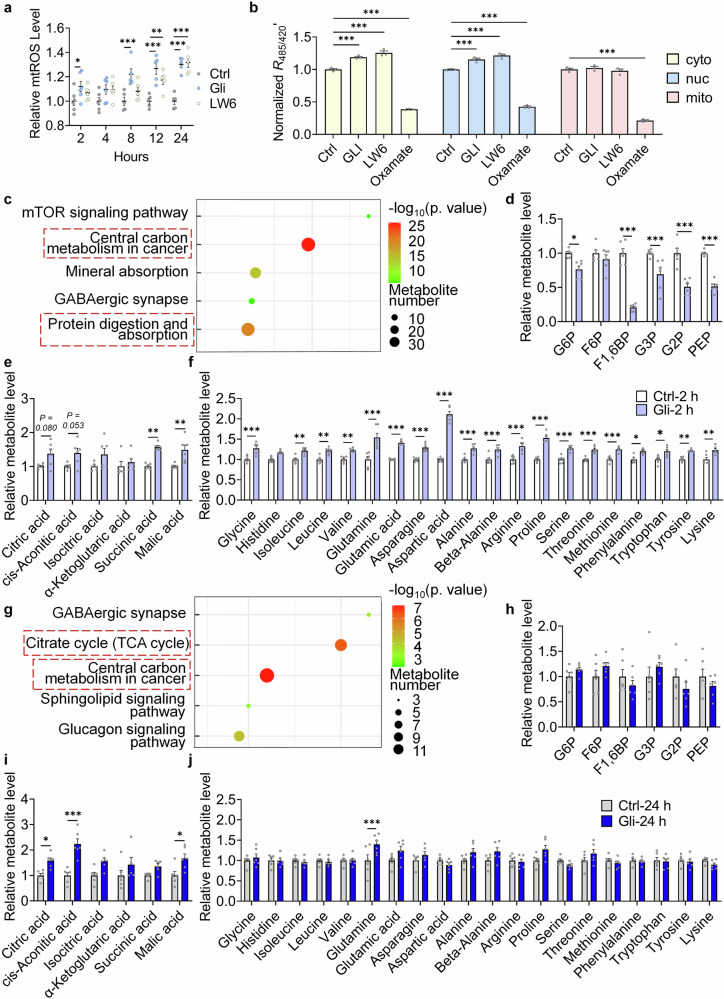
Gli targets the TCA cycle and regulates central carbon metabolism. **a** mtROS level in MEFs treated with -/Gli (100 μM)/LW6 (10 μM) for 2/4/8/12/24 h. **b** Relative fluorescence ratio indicating subcellular lactate level in H1299 cells treated with -/LW6 (10 μM)/Gli (100 μM)/oxamate (10 mM) for 2 h. **c** KEGG pathway analysis of differential metabolites in MRC-5 cells treated with -/Gli (100 μM) for 2 h. **d** Relative level of glycolysis intermediates in MRC-5 cells treated with -/Gli (100 μM) for 2 h. **e** Relative level of TCA cycle intermediates in MRC-5 cells treated with -/Gli (100 μM) for 2 h. **f** Relative level of amino acids in MRC-5 cells treated with -/Gli (100 μM) for 2 h. **g** KEGG pathway analysis of differential metabolites in MRC-5 cells treated with -/Gli (100 μM) for 24 h. **h** Relative level of glycolysis intermediates in MRC-5 cells treated with -/Gli (100 μM) for 24 h. **i** Relative level of TCA cycle intermediates in MRC-5 cells treated with -/Gli (100 μM) for 24 h. **j** Relative level of amino acids in MRC-5 cells treated with -/Gli (100 μM) for 24 h. For metabolome assays, 6 replicates were tested. Error bars represent the standard deviation (± SEM.). The significance of differences of **a** and **b** were analyzed with Tukey’s multiple comparisons tests, and other significance of differences in metabolome assays were analyzed with Sidak’s multiple comparisons tests (*p < 0.05, **p < 0.01, ***p < 0.005)

Previous studies have shown that Chl induced complex II derived mitochondrial reactive oxygen species (mtROS).^24^ Fluorescence of MitoSOX^TM^ was increased in Gli treated cells (Fig. 4a), constant with what was observed in former reports. Effects of LW6 (reported MDH2 inhibitor) on mtROS were also tested, and LW6 significantly elevated mtROS as the prolongation of the treatment (Fig. 4a), suggesting that inhibition of MDH2 mediates the upregulation of mtROS. To further investigate the effects of Gli or sh-*Mdh2* on energy metabolism, subcellular lactate level was detected using an ultrasensitive fluorescence sensor, FiLa.^28^ As an indicator of aerobic glycolysis, lactate level in the cytoplasm and nucleus was found increased under 2-h (immediate) Gli or LW6 treatment (Fig. 4b), suggesting that inhibitors of MDH2 lead to TCA cycle inhibition, which induces aerobic glycolysis.^29^

As an enzyme in the TCA cycle, MDH2 catalyzes transformation of malic acid and oxaloacetic acid using NAD^+^ as a coenzyme.^30^ We used targeted metabolome assays to investigate metabolites changes after 2-h and 24-h Gli treatments (Supplementary Table 1). In cells treated with or without Gli for 2 h, KEGG enrichment analysis indicated that the differential metabolites between groups were mainly involved in the processes of “Central carbon metabolism in cancer” and “Protein digestion and absorption” (Fig. 4c). Glycolytic intermediates were downregulated (Fig. 4d), while TCA cycle intermediates (Fig. 4e) and all 20 amino acids (Fig. 4f) that makes up proteins in living organisms were up-regulated. After 24 hours’ treatment, only metabolites in “Citrate cycle (TCA cycle)” and “Central carbon metabolism in cancer” were enriched (Fig. 4g). Level of glycolytic intermediates (Fig. 4h) and 18 amino acids (Fig. 4j) was restored, while level of TCA cycle intermediates remained upregulated (Fig. 4i). These results confirmed that Gli directly targets TCA cycle and keeps its metabolic alteration, while other changes in the metabolic pathways can be compensated in long-term treatment of Gli.

**Fig. 5:**
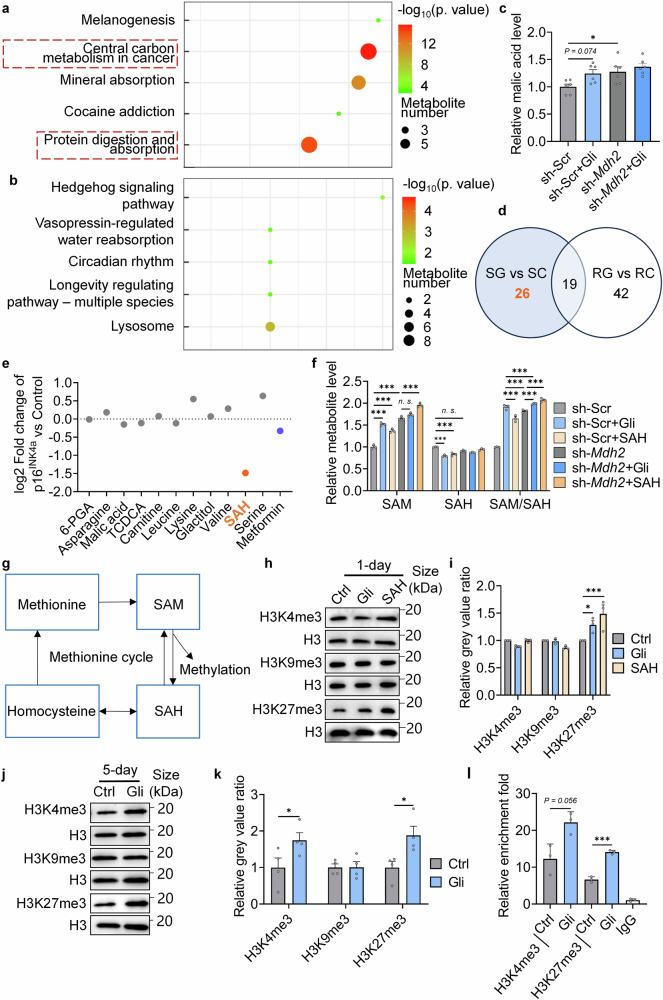
Gli regulates central carbon metabolism and methionine cycle flux through MDH2, and induces the methylation potential. **a** KEGG pathway analysis of differential metabolites in sh-Scr and sh-Scr+Gli (100 μM) groups. **b** KEGG pathway analysis of differential metabolites in sh-*Mdh2* and sh-*Mdh2*+Gli (100 μM) groups. **c** Relative level of malic acid in different groups (*n* = 6). **d** Venn graph of differential metabolites in different groups (SC: sh-Scr; SG: sh-Scr+Gli (100 μM); RC: sh-*Mdh2*; RG: sh-*Mdh2*+Gli (100 μM)). Gli-regulated metabolites dependent on MDH2 were marked in orange font. **e** Relative p16^INK4a^ level in MEFs treated with metabolites (100 μM) for 5 days. **f** Relative SAM, SAH, and SAM/SAH level in sh-Scr/sh-*Mdh2* MRC-5 cells under different treatments. **g** Diagram of methionine cycle. **h** Relative H3K4me3, H3K9me3, and H3K27me3 level in MEFs treated with -/Gli (100 μM)/SAH (100 μM) for 1 day. **i** Quantification of **h**. **j** Relative H3K4me3, H3K9me3, and H3K27me3 level in MEFs treated with -/Gli (100 μM)/SAH (100 μM) for 5 days. **k** Quantification of **j**. **l** CUT&TAG-qPCR assays testing H3K4me3 and H3K27me3 level along the p16^INK4a^ locus in MEFs treated with -/Gli (100 μM) for 5 days. Error bars represent the standard deviation (± SEM.). The significance of differences (*p < 0.05, **p < 0.01, ***p < 0.005) of all panels were analyzed with Tukey’s multiple comparisons tests

To confirm that Gli regulates central carbon metabolism through inhibiting MDH2, differential metabolites in sh-Scr and sh-*Mdh2* cells regulated by 2-h Gli treatment were measured (Supplementary Table 2). As the KEGG enrichment analysis revealed, “central carbon metabolism in cancer” was significantly enriched in sh-Scr cells treated with Gli (Fig. 5a), while knockdown of *Mdh2* expression nullified the effects of Gli (Fig. 5b). Additionally, both Gli treatment and sh-*Mdh2* elevated cellular malic acid level (Fig. 5c), indicating that the MDH2-catalyzed conversion from malic acid to oxaloacetic acid was blocked in Gli treated and sh-*Mdh2* cells. Gli also downregulated cellular amino acid level in sh-Scr cells, and this effect was nullified by sh-*Mdh2* (Supplementary Fig. 4a, b). These results demonstrate that Gli regulates metabolites in central carbon metabolism in an MDH2-dependent manner.

### MDH2 inhibition regulates SAH metabolism and histone methylation

In the targeted metabolome, 45 differential metabolites between sh-Scr (SC) and sh-Scr+Gli (SG), and 61 differential metabolites between sh-*Mdh2* (RC) and sh-*Mdh2*+Gli (RG) were detected (Supplementary Table 2). Among the 26 Gli-regulated differential metabolites dependent on MDH2 (Fig. 5d, orange font), 11 metabolites with easy commercial accessibility were supplemented to MEFs (Supplementary Table 3), and supplementation of S-adenosylhomocysteine (SAH) most significantly decreased p16^INK4a^ expression (Fig. 5e). It has been reported that appropriate supplementation of SAH can extend the lifespan of worms through simulating the synthesis of S-adenosyl-L-methionine (SAM),^31^ whereas high cellular SAH level is toxic and induce senescence in mammalian cells.^32^ Interestingly, supplementary of SAH reduced cellular SAH level and increased cellular SAM level in our model (Fig. 5f), consistent with the metabolic changes observed under Gli treatment (Supplementary Table 3). These results indicates that MDH2 inhibition enhances the methionine cycle flux, which hydrolyzes SAH to homocysteine and synthesizes SAM using methionine as a substrate (Fig. 5g). To validate the hypothesis, expression of SAH hydrolysates (*Achy* and *Achyl2*) and SAM synthase (*Mat2a*) were tested in MEFs (Supplementary Fig. 3a, b). While no difference was observed under 1-day’s treatment (Supplementary Fig. 5a), genes expression of all these enzymes were upregulated after 5-days’ treatment (Supplementary Fig. 5b), suggesting that transcriptional expression of these enzymes was upregulated under MDH2 inhibition for a longer period (5-day). As supplementary of Gli and SAH for 1-day reduced cellular SAH level and increased cellular SAM level, we supposed that the activation of the methionine cycle flux under 1-day’s MDH2 inhibition may be caused by alterations of the substrate level. In addition, expression of *Mat2a* was found elevated in sh-*Mdh2* cells compared with sh-Scr cells (Supplementary Fig. 5c), confirming the activation of methionine cycle under inhibition or knockdown of MDH2.

The ratio of SAM (a universal methyl donor) to SAH indicates the cellular methylation potential.^33^ As supplementary of Gli or SAH upregulated cellular SAM/SAH ratio (Fig. 5f), histone methylation level under treatments was tested. Among the methylation sites of histones, Gli upregulated H3K27me3 level in 1-day’s treatment while H3K4me3 and H3K9me3 level were not influenced (Fig. 5h, i). The methylation level of histones was then tested in 5-days’ Gli treatment, where the transcriptional level of enzymes in methionine cycle were elevated (Supplementary Fig. 5b). In 5-days’ treatment, tri-methylation of both H3K4 and H3K27 were increased (Fig. 5j, k). Since histone methylation is sensitive to the dynamics of methionine metabolism,^34,35^ these phenotypes supported the activation of methionine cycle flux under MDH2 inhibition. CUT&TAG-qPCR was used to test H3K4me3 and H3K27me3 level along the p16^INK4a^ locus. Consistent with the results in immunoblotting, 5-days’ Gli treatment elevated tri-methylation level at both K4 and K27 alongside p16^INK4a^ (Fig. 5l), supporting that the enhanced methylation potential caused by MDH2 inhibition affected the accessibility of p16^INK4a^ locus.

To further assay the effects of MDH2 inhibition on other cell types, mitomycin (MMC) induced senescent rat proximal renal tubular epithelial cells (NRK-52E) were used.^36^ Gli significantly decreased the SA-β-gal positive rate of MMC-induced senescent cells, while SAH did not (Supplementary Fig. 5d,e). It was observed that MMC decreased the histone methylation level of NRK-52E cells on both H3K4 and H3K27 (Supplementary Fig. 5f–i). Gli upregulated both H3K4me3 and H3K27me3 level in NRK-52E cells, while SAH only induced the tri-methylation of H3K4 (Supplementary Fig. 5f–i), suggesting that H3K27me3 acted as the critical site of methylation to delay cellular senescence under MDH2 inhibition.

### MDH2 inhibition delays liver aging and upregulates H3K27me3 in vivo

**Fig. 6:**
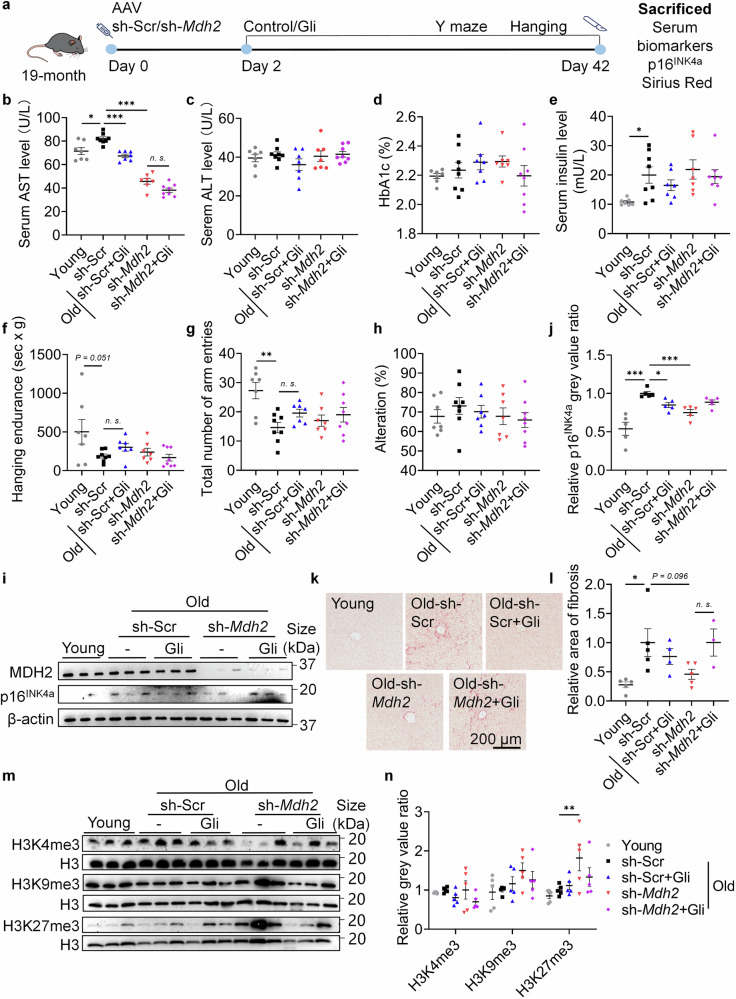
MDH2 inhibition delays hepatic aging and induces H3K27me3 level in vivo. **a** Diagram of MDH2 knockdown assay in naturally aged mice (19-month). **b** AST level of mice in different groups. Mice at 2-month age were used in Young groups. **c** ALT level of mice in different groups. **d** HbA1c ratio in serum of mice in different groups. **e** Serum insulin level of mice in different groups. **f** Behavior index of hanging endurance in mice of different groups. **g** Total number of arm entries in Y-maze of mice in different groups. **h** Alteration rate in Y-maze of mice in different groups. **i** Relative MDH2 and p16^INK4a^ level in mouse livers. **j** Quantification of **i**. **k** Sirius red staining of mouse livers. **l** Relative area of fibrosis in mouse livers, quantification of **k**. **m** Relative H3K4me3, H3K9me3 and H3K27me3 level in mouse livers of different groups (*n* = 5 for all groups). **n** Quantification of **m**. Young represents mice at 2-month age, and Old represents mice at 20.5-month age. Sample size for **b**-**h**: Young, *n* = 7; sh-Scr, *n* = 8; sh-Scr+Gli (10 mg/kg), *n* = 7; sh-*Mdh2*, *n* = 7; sh-*Mdh2+*Gli (10 mg/kg), *n* = 8. Sample size for **i**: *n* = 5 for all groups. Sample size for **k**: Young, n = 5; sh-Scr, *n* = 5; sh-Scr+Gli (10 mg/kg), *n* = 4; sh-*Mdh2*, *n* = 5; sh-*Mdh2*+Gli (10 mg/kg), *n* = 3. Error bars represent the standard deviation (± SEM.). Every dot in the plots presents the data of 1 mouse. The significance of differences (*p < 0.05, **p < 0.01, ***p < 0.005) of all panels were analyzed using Tukey’s multiple comparisons tests

Liver-specific sh-*Mdh2* through tail vein AAV-injection was used to test whether the effects of Gli are dependent on MDH2 in vivo (Fig. 6a). 2-month male mice (young) were used to compare with 20.5-month male sh-Scr/sh-*Mdh2* mice (naturally aged). Aspartate aminotransferase (AST) and alanine aminotransferase (ALT) levels are serum markers of liver injury.^37^ In mice at 20.5-month age, serum AST level (Fig. 6b) increased significantly compared to mice at 2-month age, while ALT level remained unchanged (Fig. 6c). Gli treatment and liver-specific sh-*Mdh2* significantly reduced serum AST level in mice at 20.5-month age without affecting ALT level (Fig. 6b, c), suggesting that MDH2 inhibition or knockdown reversed the age-related AST elevation without causing liver injury. Given that MDH2 gain-of-function was previously observed in familial hyperglycemia cases,^38^ we tested serum glycosylated hemoglobin (HbA1c) ratio to determine if Gli treatment or liver-specific sh-*Mdh2* would cause long-term blood glucose fluctuation. Gli administered at 10 mg/kg or sh-*Mdh2* did not significantly alter HbA1c level in mice at 20.5-month age (Fig. 6d), indicating stable blood glucose level in Gli or sh-*Mdh2* treated mice. Serum insulin level in mice at 20.5-month age was higher than mice at 2-month age (Fig. 6e), suggesting early-stage of insulin resistance in naturally aged mice. Neither Gli treatment nor *Mdh2* knockdown significantly altered the insulin level, further confirmed that the homeostasis of glucose metabolism was maintained under MDH2 inhibition or knockdown. The hanging endurance (Fig. 6f) and the total arm entries in Y-maze test (Fig. 6g) of mice at 20.5-month age were lower than the endurance and arm entries of mice at 2-month age, suggesting degeneration of physical abilities during the aging process. Gli and liver-specific sh-*Mdh2* slightly improved the physical performance of mice, while these improvements were unsignificant (Fig. 6f,g). The alteration rate of mice in Y-maze was also tested, and no difference were found between groups (Fig. 6h).

Testing effects on hepatic aging, Gli and sh-*Mdh2* significantly reversed the elevated level of p16^INK4a^ in the livers of mice at 20.5-month age, while sh-*Mdh2* nullified the effects of Gli (Fig. 6i, j). This result demonstrates that Gli relies on MDH2 to exert its anti-aging effect in vivo. As a sign of liver aging, hepatic fibrosis level was also tested.^39^ As expected, sh*-Mdh2* reduced the fibrosis area in the livers of mice at 20.5-month age (Fig. 6k, l), confirming that MDH2 inhibition delays hepatic aging. Effects on histone methylation were also observed in vivo. Sh-*Mdh2* barely affected H3K4me3 while significantly upregulated H3K27me3 in livers of naturally aged mice (Fig. 6m, n). Since H3K27me3 help inhibit transcriptional activation of p16^INK4a^ in the process of cellular senescence,^40,41^ these results supported that MDH2 inhibition remodels histone methylation and stabilizes the chromosome to relieve aging phenotypes.

**Fig. 7:**
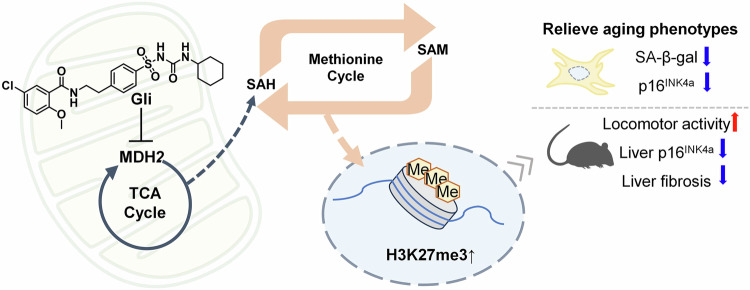
Diagram of the anti-aging effects of metabolic-epigenetic regulation under MDH2 inhibition

In summary, our findings demonstrated that MDH2 inhibition (via Gli or sh-*Mdh2*) interferes the TCA cycle, enhances the methionine cycle flux, inducing methylation modification on H3K27me3, and thereby alleviating cellular senescence and tissue aging through metabolic-epigenetic regulation (Fig. 7).

## Discussion

In this study, we performed ABPP and discovered MDH2 as an anti-aging target that can be inhibited by Gli. MDH2 is a mitochondrial enzyme in the TCA cycle and malate-aspartate shuttle, conducting conversion of malic acid and oxaloacetic acid.^15^ Inhibiting MDH2 has been shown to alleviate anoxia-reoxygenation-induced senescence in renal tubular epithelial cells by reducing TCA cycle-produced electron donors and ETC-derived oxidative stress.^42^ However, the role of MDH2 in replicative senescence of cells and natural aging of mammals has not been clearly elucidated. Here we found that inhibition or knockdown of MDH2 alleviates aging phenotypes in replicative senescent fibroblasts and naturally aged mice livers, whereas overexpression of MDH2 exacerbates replicative cellular senescence in fibroblasts. These results validate MDH2 as an amenable target for aging intervention in the metabolic pathways.

MDH2 inhibition was found to upregulate cellular SAM/SAH ratio (Fig. 5f), possibly due to alterations of serine and folate-mediated one-carbon metabolism caused by TCA cycle blockage.^43,44^ Serine can be dehydroxymethylated to glycine while converting tetrahydrofolate (THF) to 5,10-methylene-THF in mitochondria.^45^ When malate oxidation is disrupted by MDH2 inhibition, more mitochondrial NAD^+^ can be utilized to produce 10-formyl-THF and transport formate to the cytoplasm. Thus, inhibiting MDH2 provides more methyl groups to enhance the methionine cycle flux (Supplementary Fig. 6). Additionally, expression of enzymes involved in SAH hydrolysis and SAM synthesis were found to be upregulated under 5-days’ MDH2 inhibition (Supplementary Fig. 5b, c), proving the enhanced metabolic flux of methionine cycle. Also, methylation level of histone H3 were upregulated under Gli treatment and *Mdh2* knockdown (Fig. 5j, k and Fig. 6m, n), where H3K27me3 played a crucial role in reversing cellular senescence (Supplementary Fig. 5d–i). Thus, our results validate MDH2 as an anti-aging target regulating the metabolic-epigenetic circuit.

Among the tested sulfonylureas, Gli exhibited the highest MDH2 inhibition activity. As a hypoglycemic drug, Gli induces insulin secretion, but is also reported to cause “secondary failure” of insulin secretion capacity in long-term usage on diabetic patients.^46^ Since both insulin resistance and failure of insulin secretion feature the age-related metabolic disorders, we tested the serum insulin level in aged mice. Neither Gli supplementation nor *Mdh2* knockdown affected the secretion of insulin in our 40-day model (Fig. 6e). However, whether inhibition of MDH2 in a longer extent can cause degradation of insulin secretion capacity or insulin sensitivity shall be clarified. In addition, inhibition of MDH2 forces the activation of mito-cytoplasm shuttle of malic acid^42,47,48^ (Supplementary Fig. 7), which may increase the vulnerability of energy metabolism under metabolic stress. Thus, effects of lifetime MDH2 blockage on glycose, lipid and protein metabolism and the hormones regulating metabolic pathways shall be tested to ensure the security. Though patients with age-related liver fibrosis might benefit from MDH2 inhibition (Fig. 6k, l), usage of Gli must be under supervision to avoid hypoglycemic effects and other negative consequences on energy metabolism.

Besides the anti-senescence effects of Gli dependent on MDH2, effects of Gli independent of MDH2 were also observed. Gli was found to reduce OCRs, thereby increasing the coupling efficiency of oxidative phosphorylation independent of MDH2 (Supplementary Fig. 8a–d), suggesting the existence of other targets enhancing the coupling efficiency. Also, we found that Gli decreased IL-6 expression in hepatic tissues from both sh-Scr and sh-*Mdh2* mice (Supplementary Fig. 8e). Consistently, Gli inhibited serum IL-6, MMP3, and GDF15 (Supplementary Fig. 8f–h) and decreased the transcription level of *Il-6* in sh-*Mdh2* MEFs (Supplementary Fig. 8i), showing that Gli have additional targets in regulating SASP. Supportively, Gli was reported to be a broad-spectrum inhibitor of ABC transporters suppressing IL-6 and MMP3 downstream of NF-κB - ABCA1 axis.^49^ In addition, the expression of IL-6 was barely activated in cellular senescence induced by methylation loss of H3^50^ and inhibition of CDKs.^51,52^ As regulation of p16^INK4a^ dedicate to unique SASP profiles without conventional NF-κB driven SASP,^53^ our results suggest that Gli acts on both MDH2 and ABC transporters, and thus exerts much border suppressive effects on SASP.

In conclusion, our study introduces a novel approach for relieving aging phenotypes by targeting MDH2, thereby enhancing methionine cycle flux and reversing age-related alterations in histone methylation. As a small molecule inhibitor of MDH2, Gli presents a promising lead compound that can be modified to achieve optimal MDH2 inhibition activity and pharmacokinetic properties. Derivatives based on Gli can be developed to better target MDH2 and hold great therapeutic potential for relieving aging and mitigating age-related diseases through metabolic intervention.

## Materials and methods

### Ethic statements

Animal handling and procedures were performed in accordance with National Institutes of Health guidelines, and approved by the ethical committee of Hainan University (contract No. HPIACUC2023083).

### MDH2 activity assays

The coding region for amino acids 20-338 of human MDH2 was synthesized by IGE Biotechnology Co., Ltd (Guangzhou), and subcloned into the expression vector pET28a. The resultant plasmid pET28a-MDH2 was transferred into *Escherichia coli* strain BL21 (Codonplus) for overexpression, which was grown in LB medium at 37 °C until A600 of 0.6, and 0.1 mM IPTG was added for further growth at 15 °C for 24 h. The recombinant proteins were purified by Ni-NTA column (Qiagen), Q-column (GE Healthcare), and Superdex 100 column (GE Healthcare). A typical purification yielded over 10 mg of MDH2 with a purity of > 95% from a 12-liter cell culture.

MDH2 activity was measured by testing consumption of NADH through absorbance of 340 nm. For brief, 1 nM MDH2 (4×), 0.8 mM NADH (4×), and 4% DMSO solution with different compounds were mixed and added to a 96-well plate. 2.4 mM oxaloacetic acid (4×) was added and immediately started measurement of absorbance at 340 nm on the microplate reader (Bio-Tek Instruments, Synergy H1). The absorbance was measured every minute for 30 min. Relative MDH2 activities were calculated by the formula: (OD_tested, 0 min_ - OD_tested, 30 min_) / (OD_blk, 0 min_ - OD_blk, 30 min_).

### In situ labelling and pulldown with Chl-P/Gli-P

Chemical probes of Chl (Chl-P) and Gli (Gli-P) were used for targets discoveries and confirmations.

Chl-P was used for pulldown experiments. Briefly, MRC-5 cells were grown to 90% confluency and treated with FBS-free MEM containing 200 μM Chl-P for 1 h. 2 mM Chl was used for competition. UV light of 365 nm was used to activate the photoreactive group (diazirine) of Chl-P, and formed an in situ covalent bond between Chl-P and targeted proteins. After labeling, cells were lysed and diluted to 1 mg/kg. Lysates were incubated with freshly prepared click chemistry reaction cocktail (20 μM Biotin-N_3_, 50 μM TBTA, 0.5 mM TCEP, and 0.5 mM CuSO_4_) for 2 h, and then loaded on Capturem Streptavidin Miniprep Columns (Tkara, 635733) for pulldown experiments as previously described.^54^ Proteins were collected, separated by SDS-PAGE, and visualized through silver staining. Protein bands between 30-40 kD were cut and entrusted to Bio-Tech Pack Technology Co., Ltd. (Beijing) for protein identification. Briefly, protein bands were washed with a solution containing 15 mM K_3_Fe(CN)_6_ and 50 mM Na_2_S_2_O_3_ to remove the staining, and then acetonitrile and 10 mM DTT/50 mM ammonium bicarbonate. Trypsin was used for digestion. 5% TFA-50% CAN-45% ddH_2_O were used for extraction of peptides, lyophilized, resuspended in 0.1% formic acid, and loaded for LC-MS/MS analysis. Data was analyzed and searched against target protein database of human using MaxQuant (1.6.2.10). Proteins with molecular weight between 30-40 kD and more than 5 unique peptides were defied as protein of high confidence. Potential targets with mass intensity highest in Chl-P treated groups were listed as potential targets.

For confirmation, Gli-P was used to label MRC-5 cells under UV irradiation. Protein lysates of labeled cells were loaded on Capturem Streptavidin Miniprep Columns (Takara, 635733) for pulldown experiments. Immunoblotting was used to detect MDH2 in pulldown samples.

### Grating-Coupled Interferometry

Experiments were performed with the Creoptix WAVEdelta system (Creoptix) using PCP WAVE chips (thin quasiplanar polycarboxylate surface). Chips were first conditioned with borate buffer (100 mM sodium borate, 1 M NaCl, pH 9.0). Then, the chips were activated with 1:1 mix of 400 mM N-(3-dimethylaminopropyl)-N′-ethylcarbodiimide hydrochloride and 100 mM N-hydroxysuccinimide. Recombinant MDH2 (20 μg/mL) in 10 mM sodium acetate (pH 5.0) was immobilized on the chip surface until saturation was reached. Passivation and quenching step were conducted with 1 M ethanolamine (pH 8.0). Multi-cycle kinetic analyses were performed to measure the binding of Gli to MDH2. All experiments were performed at 25 °C with a 1:2 dilution series from a maximum concentration of 50 μM, in running buffer (1×HBS-EP+, 10 mM HEPES, 150 mM NaCl, 3 mM EDTA, 0.005% P20, pH 7.4,) with addition of 3% DMSO. Blank injections were used for double referencing, and a DMSO calibration curve was used for bulk correction. Analysis and correction of the obtained data were performed using the Creoptix WAVE control software (correction applied: X and Y offset; DMSO calibration; double referencing). A one-to-one binding model was used for the experiment.

### Animals and drug treatment

Male C57BL/6 J mice were purchased from Jiangsu Aniphe Biolaboratory Co., Ltd. Mice were housed in a SPF animal facility and kept on a regular diet with an environmental temperature control at 21–24 °C and a 12 h light-dark cycle. Maximum 5 mice were housed in 1 cage, and aggressive males were isolated to prevent fighting. Mice were inspected daily by the veterinary staff. Health screening was done at 3 months intervals, which is consist of serological screening and fecal and fur analysis for internal and external parasites.

For lifespan assay, Male C57BL/6 mice aged 12-month were randomly arranged into three groups: Ctrl group (solvent, *n* = 10), Gli group (Gli, 10 mg/kg, *n* = 12) and NMN group (NMN, 500 mg/kg, *n* = 10). For Control and Gli groups, solvent/Gli was given daily through intragastric administration. The solvent in the Control group was the same as that in the Gli group, which was 15% PEG400 + 85% saline. NMN was dissolved in water of NMN group. Water consumption was measured every 3 days to ensure an average NMN dosage as 500 mg/kg/d.

Frailty index was tested using the same mice in the lifespan assay started after 6-month solvent/Gli/NMN treatment (18-month-age), and was conducted according to previous methods.^20,55^ The frailty index includes a total of 31 indicators in 6 aspects, including integument (alopecia, loss of fur color, dermatitis, loss of whiskers and coat condition), physical (tumors, distended abdomen, kyphosis, tail stiffening, gait disorders, tremor, forelimb grip strength and body condition score), vestibulocochlear (vestibular distance, hearing loss, cataracts, eyes discharge/swelling, microphthalmia, corneal opacity, vision loss, menace reflex, and nasal discharge), digestive (malocclusions, rectal prolapse, vaginal/penile prolapse, diarrhea), respiratory system (breathing rate/depth), discomfort and others (mouse grimace, piloerection, temperature and weight). 0 (no sign of frailty), 0.5 (moderate phenotype) and 1 (severe phenotype) were used to evaluate the above indicators. Mice were tested once a month, and the frailty index was calculated as the average score of 31 indicators.

For drug treatment after tail vein injection of AAV packaged of sh-Scr or sh-*Mdh2*, Gli was dissolved in solvent containing 85% saline and 15% PEG400 to a final concentration of 1 mg/mL, and then administered by *intragastric administration* for 40 days. Mice were sacrificed on the 40^th^ day of Gli/solvent treatment, 8 h after intragastric administration. Serum was collected by centrifuging at 3,000 g/min, 4 °C, and used for ELISA assays. Hepatic tissues were collected on ice and stored at −80 °C before assays.

### Cells lines and senescence induction

MEFs were isolated from pregnant C57BL/6 J mice at 13.5 - day post coitum. The extraction process was in accordance with a previously described protocol^56^: Embryos were isolated from embryo sacs, and heads and limbs were removed. The remaining tissues were subjected to trypsinization in 0.05% trypsin/EDTA (Gibco, 25300-062) with DNAase I (Solarbio, D8071). Trypsin was inactivated by adding Dulbecco’s modified Eagle’s medium (DMEM, Gibco, C11995500BT), and cells were collected through centrifuging at low-speed (799 g, 5 min). The isolated MEFs were cultured on plates coated with 0.1% gelatine (sigma, G9391-100G) for 12 h and frozen when reaching approximately 90% confluency (P0). MEFs were cultured in DMEM (Gibco) supplemented with 10% fetal bovine serum (FBS, Gibco, 10099-141) and 1% penicillin/streptomycin (Yeasen, 60162ES76), and passaged when reaching 90% confluency. Replicative senescent MEFs (P7) were used for further assays.

MRC-5 cells were obtained from Cell Bank of Chinese Academy. MRC-5 cells were grown in Minimum Essential Medium (MEM, Gibco, 11090-081) with 10% FBS (Biochannel, BC-SE-FBS01), 1% penicillin/streptomycin (Yeasen, 60162ES76), 1% sodium pyruvate solution (Gibco, 11306-070), 1% GlutaMAX (Gibco, 335050-061) and 1% nonessential amino acids solution (Gibco, 11140-050). MRC-5 cells were treated with Dox (100 nM) every other day for the first three days, and cultured for the next four days to induce senescence.^57^ Drugs to be tested were added to MEM during the process. For replicative senescence, MRC-5 cells passaged to more than P42 was used.

NRK-52E cells were obtained from Cell Bank of Chinese Academy. NRK-52E cells were cultured in DMEM (Gibco) supplemented with 10% fetal bovine serum (FBS, Gibco, 10099-141) and 1% penicillin/streptomycin (Yeasen, 60162ES76), and passaged when reaching 90% confluency. NRK-52E cells were treated with MMC (0.5 μM) for 2 days, and cultured for the next 2 days to obtain MMC-induced senescence.^37^ Drugs to be tested were added to DMEM after removal of MMC, and kept for 2 days before assays.

H1299 cells HEK293T cells were cultured in RPMI 1640 medium (Corning, 10-040-CV) supplemented with 10% fetal bovine serum (Vivacell, C04001-500). To generate stable cell lines for subcellular lactate detection, the pLVX lentiviral plasmids encoding FiLa sensors were constructed. Lentivirus was produced by co-transfecting two lentiviral packaging vectors (pMD2.G and psPAX2) in HEK293T cells. Lentiviral supernatants were collected 48 and 72 h after transfection. H1299 cells in 6-well tissue culture plates were infected in media containing 8 mg/mL polybrene. After infection, the virus was removed, and cells were selected with 0.2-1 µg/mL puromycin for one week to obtain stable cell lines.

### Live-cell lactate measurement using FiLa

H1299 cells stably expressing FiLa, FiLa-Nuc, FiLa-Mit, FiLa-C, FiLa-C-Nuc and FiLa-C-Mit were seeded in 96-well black bottom plates. After 12-16 h, cells were treated with different compounds for 2 h. Then, the cells were washed twice with HBSS containing 25 mM glucose after carefully removing the medium. Data was recorded immediately after the addition of 100 μL HBSS with 25 mM glucose at 37 °C by a Synergy Neo2 Multi-Mode Microplate Reader (BioTek) with 420 BP 27 nm and 485 BP 20 nm excitation filters and a 532 BP 40 nm emission filter. Fluorescence values were background corrected by subtracting the intensity of the cell samples not expressing sensors.^28^

### Mitochondria ROS level measurement

mtROS level of MEFs was measured using a microplate reader (Bio-Tek Instruments, Synergy, H1). Cells were incubated with -/Gli for 2, 4, 8, 12, and 24 h. These cells were then washed by PBS, stained by MitoSOX^TM^ (5 μM) for 30 min, washed by PBS again, and measured using a microplate reader. 12 wells were measured for each group and MitoTracker^TM^ Green FM (200 nM) was used for normalization. Significance was determined using the 2-way ANOVA.

### Lentivirus construction and cellular infection

Constructions of Lentivirus were entrusted to Tai Leng Biotechnology Co., Ltd (Shanghai).

MEFs were transduced with a *Mdh2* shRNA or overexpression vector. shRNA sequences are as follows:

sh-Scr: 5′ - CGCTGAGTACTTCGAAATGTC - 3′.

sh-*Mdh2*: 5′ - GAGCAAATGTGAAAGGCTACC - 3′.

The sequence of *Mdh2* overexpression is as shown on https://www.ncbi.nlm.nih.gov/nuccore/NM_008617.2.

The empty vector was packaged as the negative control for MDH2-OE.

MEFs were seeded into a 6-well plate in the number of 3×10^^^5. Once adhered, MEFs were transfected with lentivirus in DMEM containing 5 μg/mL polybrene (shRNA: MOI = 60; OE: MOI = 15) for 24 h. Following infection, the culture medium was replaced with fresh DMEM for 2 days, and then replaced with DMEM containing 0.5 μg/mL puromycin for 2-day selection. Subsequently, lentivirus-transfected MEFs were cultured in and passaged when confluence in DMEM with 0.25 μg/mL puromycin. The knockdown efficiency was assessed on the 7^th^ day post-transfection.

MRC-5 cells were then transduced with a *Mdh2* shRNA vector. *Mdh2* shRNA sequences are as follows:

sh-Scr: 5′ - GTCTCCACGCGCAGTACATTT - 3′.

sh-*Mdh2*: 5′ - GCCACTTTCACTTCTCCTGAA - 3′.

MRC-5 cells were seeded into a 6 cm cell culture dish in the number of 3 × 10^^^5. Once adhered, MRC-5 cells were transfected with lentivirus in MEM containing 5 μg/mL polybrene (shRNA: MOI = 4) for 12 h. Following infection, the culture medium was replaced with fresh MEM for 2 days, and then replaced with MEM medium containing 2 μg/mL puromycin for 48-h selection. Lentivirus-transfected MRC-5 cells were cultured in and passaged when confluence in MEM containing 0.5 μg/mL puromycin. The knockdown efficiency was assessed on the 7th day post-transfection.

### Construction of AAV packaged plasmids and mice infection

Construction of AAV packaged of sh-Scr/sh-*Mdh2* interfering plasmid with liver-specific promoter was entrusted to Tai Leng Biotechnology Co., Ltd (Shanghai). The sequences are as follow:

sh-Scr: 5′ - GTCTCCACGCGCAGTACATTT - 3′

sh-*Mdh2*: 5′- AGCTGAAAGCCTCCATCAAGA -3′

Male C57BL/6 J mice were injected with AAV (1E + 11VG units per 25 g body weight of mice) via the tail vein.

### SA-β-gal staining

Cells were washed with PBS once and fixed with 0.2% glutaraldehyde and 2% formaldehyde in PBS for 5 min at room temperature. Washing three times with PBS, MRC-5 cells or MEFs were incubated with SA-β-gal staining solution (1 mg/mL X-gal, pH = 6.0) overnight at 37 °C. After SA-β-gal staining, the cells were washed with PBS for three times and stained nucleus with Hoechst 33258 (Keygen, 1:1000 dilution).

The images of SA-β-gal staining were taken by the inverted fluorescence microscope (Nikon, Ti-S), and processed in ImageJ. For cells, SA-β-gal positive cells rate was calculated by the number of X-gal-stained cells dividing the number of total cells. Quantification was obtained from more than 15 photos of one biological replication.

### Targeted LC-MS metabolomics analyses

Wild-type MRC-5 cells were treated with Gli for 2 h and 24 h. sh-Scr/sh-*Mdh2* MRC-5 cells were treated with Gli for 2 h.

All cells were collected on ice and the numbers of cells were recorded for normalization.

Targeted metabolomics assays were entrusted to Applied Protein Technology Co., Ltd (Shanghai). In brief, samples were washed with precooled PBS and metabolites were extracted with precooled methanol/acetonitrile/water solution (2:2:1, v/v) to remove the protein. The mixture was centrifuged in 14,000 g for 20 min at 4 °C, and then supernatant was vacuum dried.

For LC-MS analysis, samples were redissolved in acetonitrile/water solution (1:1, v/v). Metabolites were separated by UHPLC, using an Agilent 1290 Infinity LC column, and tested by 6500 Q-trap system (AB SCIEX). Peak areas and insets of each substance were obtained by peak extraction from raw MRM data using MultiQuant or Analyst software. The ratio of the labeled peak area, and the content was calculated from the standard curve. The significant differential metabolites (*t*-test *p* value < 0.05) were used for KEGG pathway analysis.

### Physical functions

All physical functional assays on mice were conducted one week before sacrifice.

For Y-maze assay, arms of the Y maze (Shanghai Xinruan Information Technology Co., Ltd, XR-XY1032) were denoted as A, B, and C. Mice were placed in arm A and allowed to move freely. The numbers of arm entries were recorded and the alteration rates were tested for 8 min. Alternation (%) = number of alternations / (total number of arm feeds-2) × 100%.

For grip strength assay, mice were placed in the grid of the grip strength meter (Shanghai Xinruan Information Technology Co., Ltd, XR501) and pulled backward. Peak grip strength was recorded. The tests were repeated three times and the averaged grip strength was used for analysis.

For treadmill assay, mice were trained for 3 days before formal experiments. The training condition was conducted on a 5° slope (5 m/min, 2 min; 7 m/min, 2 min; 9 m/min, 1 min). In formal experiments, the running speed was started at 5 m/min and steadily increased by 2 m/min every 2 min until mice were exhausted. The total work was calculated as the formula: body weight (kg) ×g (9.8 m/s^2^) × distance (m) × sin (5°).

For rotarod assay, mice were placed on the rod (Shanghai Xinruan Information Technology Co., Ltd, XR-6C) rotating at an initial speed of 4 rpm/min, and then accelerated from 4 to 40 rpm/min within 300 s. The time that mice stayed on the rod was recorded for consecutive 3 days and the results were averaged from three tests.

For hanging endurance assay, mice were ensured to grasp a 2.5 mm metal wire with their forelimbs, and the hanging time was recorded. The results were normalized to body weight as hanging duration (s) × body weight (g), and then the results were averaged from three tests.

### Western blotting

Mouse livers were suspended in RIPA lysis buffer (Strong) (Yeasen, 20201ES60), and homogenized with tissue homogenizer (Servicebio, KZ-III). Homogenates were centrifuged in 12,000 g for 15 min at 4 °C, and the suspensions were collected. 5× protein loading buffer was added to the suspensions, and heated (95 °C, 10 min) to prepare protein lysates ready for electrophoresis. For cells, protein lysis was conducted using RIPA lysis buffer (Weak) (Yeasen, 20114ES60). Ultrasonication was used for homogenization of samples.

Protein lysates were loaded onto the SDS-PAGE gel and subjected to electrophoresis. After that, proteins were transferred to a nitrocellulose (NC) membrane and subjected to immunoblotting with the indicated antibodies. Immunoblotting images were acquired using an imaging system (Tanon-4600SF). The following antibodies were used in this study: anti-β-actin (Abclonal, AC026), anti-HSP90 (Abcam, ab203126), anti-β-Tubulin (Solarbio, K200059M), anti-p16^INK4a^ (Abcam, ab211542), anti-p16^INK4a^ (Proteintech, 10883-1-AP), anti-MDH2 (Abcam, ab250541), anti-MMP1 (Abcam, 134184), anti-pAMPK alpha (Thr 172) (CST, 2535), anti-AMPK alpha (Proteintech, 10929-2-AP), anti-H3 (Abclonal, A2348), anti-H3K4me3 (Abclonal, A22225), anti-H3K9me3 (Abclonal, 22295), anti-H3K27me3 (Abclonal, 22396), Peroxidase AffiniPure Goat Anti-Rabbit IgG (H + L) (Yeasen, 33101ES60).

### Quantitative real-time PCR

Total RNA was extracted, purified, and concentrated from MEFs using an RNA Isolation Kit (Yeasen, 19221ES50), and reverse transcripted to cDNA using a reverse transcription kit (Yeasen, 11141ES60). The mRNA expression of *Il-6* was analyzed using the Hieff UNICON Universal Blue qPCR SYBR Green Master Mix (Yesen, 11184ES08) on qPCR detecting system (BIO-RAD, CFX96). *Gapdh* was used as a control to normalize the expression of target genes using the -ΔΔCt method. The primers were used as follows:

*Tuba1a*, forward: 5' - CCGCGAAGCAGCAACCAT - 3'; reverse: 5' - CCAGGTCTACGAACACTGCC - 3'.

*Il-6*, forward: 5' - TGTTCTCTGGGAAATCGTGGA - 3'; reverse: 5' - TGCAAGTGCATCATCGTTGTTC - 3'.

*Ahcy*, forward: 5' - CCCTACAAAGTCGCGGACATC - 3'; reverse: 5' - GAGGCTGAGTACATCTCCCG - 3'.

*Ahcyl2*, forward: 5' - ATGTCGGTGCAGGTTGTGTC - 3'; reverse: 5' - GGGCTCAGATCCTTCAGCTC - 3'.

*Mat2a*, forward: 5' - GCTTCCACGAGGCGTTCAT - 3'; reverse: 5' - AGCATCACTGATTTGGTCACAA - 3'.

*Mdh2*, forward: 5' - TTGGGCAACCCCTTTCACTC - 3'; reverse: 5' - GCCTTTCACATTTGCTCTGGTC - 3'.

### ELISA analysis

SASPs were tested by ELISA kits. The cell media was collected and centrifuged at room temperature in a speed of 799 rpm for 5 min at the end of treatments. The serum of mice was sampled from blood which separated from the eyeballs, and then centrifuged at 4°C in a speed of 13000 rpm for 15 min. After centrifugation, they were collected and stored at −80 °C until use. The liver samples were directly frozen in liquid nitrogen. The kits used were as follows IL-1β: RF7630; IL-6: RF6857; MMP3: RF2117, GDF15: RF2004, SAM: RF8761, SAH: RF8767, insulin: RF8164. All ELISA data was analyzed by Ruifan Biological Technology.

### Measurements of fibrosis area

Picrosirius red was used to stain the fibrosis area on mouse liver slices. Briefly, paraffin sections were dewaxed to water, washed three times with PBS, and stained with picrosirius red buffer for 8 min. Sections were washed with anhydrous ethanol for three times, dehydration to wax, and sealed with neutral resin. Images were taken using an inverted fluorescence microscope (Nikon, Ti-S) with 30× amplification. Photos were processed in ImageJ for measurement of fibrosis area.

### OCR tests

Sh-Scr/sh-Mdh2/OE-NC/OE-Mdh2 MEFs were plated in XF Pro Plated (Agilent, 103794-100) at 20,000 per well. Cells were treated with -/Gli (100 μM) for 1 h in a CO2 incubator at 37 °C before the experiment. The medium was replaced to Seahorse XF DMEM (Agilent, 103575-100) containing 10 mM glucose, 1 mM pyruvate, 2 mM glutamine and -/Gli (100 μM), and cells were maintained in a non-CO2 incubator at 37 °C for 1 h. 20 μL of 15 μM oligomycin, 22 μL of 5 μM FCCP, 25 μL of 5 μM Rotenone and 5 μM Antimycin were loaded into the injection ports of the XFe-96 sensor cartridge. During the experiment, the instrument (Agilent, Seahorse XFe96) injected the inhibitors into the wells at a given time point, while OCR was measured continuously. Data were analyzed by XFe-96 software.

### CUT&TAG-qPCR

CUT&TAG-qPCR was conducted using Hyperactive Universal CUT&TAG Assay Kit for Illumina Pro with CUT&Tag Stop Buffer for qPCR (Vazyme, TD904-C1) and Taq Pro Universal SYBR qPCR Master Mix (Vazyme, Q712) as the manufacturers’ instructions. Briefly, 1 × 10^5^ MEFs treated with -/Gli (100 μM) for 5 days were collected and treated with ConA beads. Anti-H3K4me3 (Abclonal, A22225), anti-H3K27me3 (Abclonal, 22396) and anti-IgG (Proteintech, 30000-0-AP) were incubated with cells overnight at 4 °C, and reacted with secondary antibodies (Vazyme, Ab207-01-AA) for 1 h at room temperature after being washed for 3 times. Samples were then incubated with pA/G-Tnp Pro for 1 h. DNA fragmentation was performed, and DNA Extract Beads Pro were used to collect DNA fragments. Stop Buffer was added, and heated at 95 °C for 5 min to release DNA. DNA spike-in was added as reference, and qPCR was conducted using primers as follows:

DNA spike-in, former: GCCTTCTTCCCATTTCTGATCC; reverse: CACGAATCAGCGGTAAAGGT.

*Cdkn2a*, former: GCTATGCCCGTCGGTCTG; reverse: ACATCGTGCGATATTTGCGTT.

### Statistics and drawings

Statistical analyses were performed by GraphPad 8.3.0. Unpaired student’s *t*-tests (two-tailed) were used for comparation between 2 groups. Dunnett’s multiple comparisons tests or Tukey’s multiple comparisons tests were used for comparation of 3 or more samples affected by single-factor. Sidak’s multiple comparisons tests or Tukey’s multiple comparisons tests were used for comparation of 3 or more samples affected by 2 factors. Statistical significance is represented in the figures by *p < 0.05, **p < 0.01, ***p < 0.001. For in vitro studies, samples were measured repeatedly, and at least 2 distinct samples were collected. For in vivo studies, mice were randomly assigned to treatment groups, and all replicates in this work represent different mice. G*Power 3.1.9.7 was used to calculate power of assays, and to ensure the sample size used in the present study was enough to obtain an actual power greater than 0.95. Data was normalized to the Ctrl/Dox/MMC/sh-Scr group. PowerPoint of Microsoft Office 365 was used for creating diagrams in Fig. 7 and Supplementary Figs. 6, 7.

## Supplementary information

Supplementary Table 1

Supplementary Table 2

Supplementary Metarials

Supplementary Data 1

Supplementary Data 2

## Data Availability

All the data during the current study are available within the paper and its Supplementary information, or from the corresponding author upon reasonable request.
