# Supplementary material for: Glibenclamide targets MDH2 to relieve aging phenotypes through metabolism-regulated epigenetic modification
Source: Signal Transduct Target Ther. 2025 Feb 17;10:67. doi: 10.1038/s41392-025-02157-3 (PMC11833132; doi:10.1038/s41392-025-02157-3)
Supplement: Supplementary file 3 — Supplementary Metarials [file 41392_2025_2157_MOESM3_ESM.docx]

Supplementary Materials for

Glibenclamide targets MDH2 to relieve aging phenotypes through metabolism-regulated epigenetic modification

Zhifan Mao^2†^, Wenwen Liu^1†^, Rong Zou^2†^, Ling Sun^1†^, Shuman Huang^2^, Lingyu Wu^1^, Liru Chen^1^, Jiale Wu^1^, Shijie Lu^2^, Zhouzhi Song^2^, Xie Li^2^, Yunyuan Huang^3^, Yong Rao^1^, Yi-You Huang^1^, Baoli Li^1*^, Zelan Hu^2*^, Jian Li^1,2,4*^

Correspondence to: jianli@ecust.edu.cn (J. L.); huzelan@ecust.edu.cn (Z. H.); baolili@hainanu.edu.cn (B. L.)

**This PDF file includes:**

Figures. S1 to S8

Tables S3

**Other Supplementary Materials for this manuscript include the following:**

Data S1 to S2

Tables S1 to S2

Figure. S1.


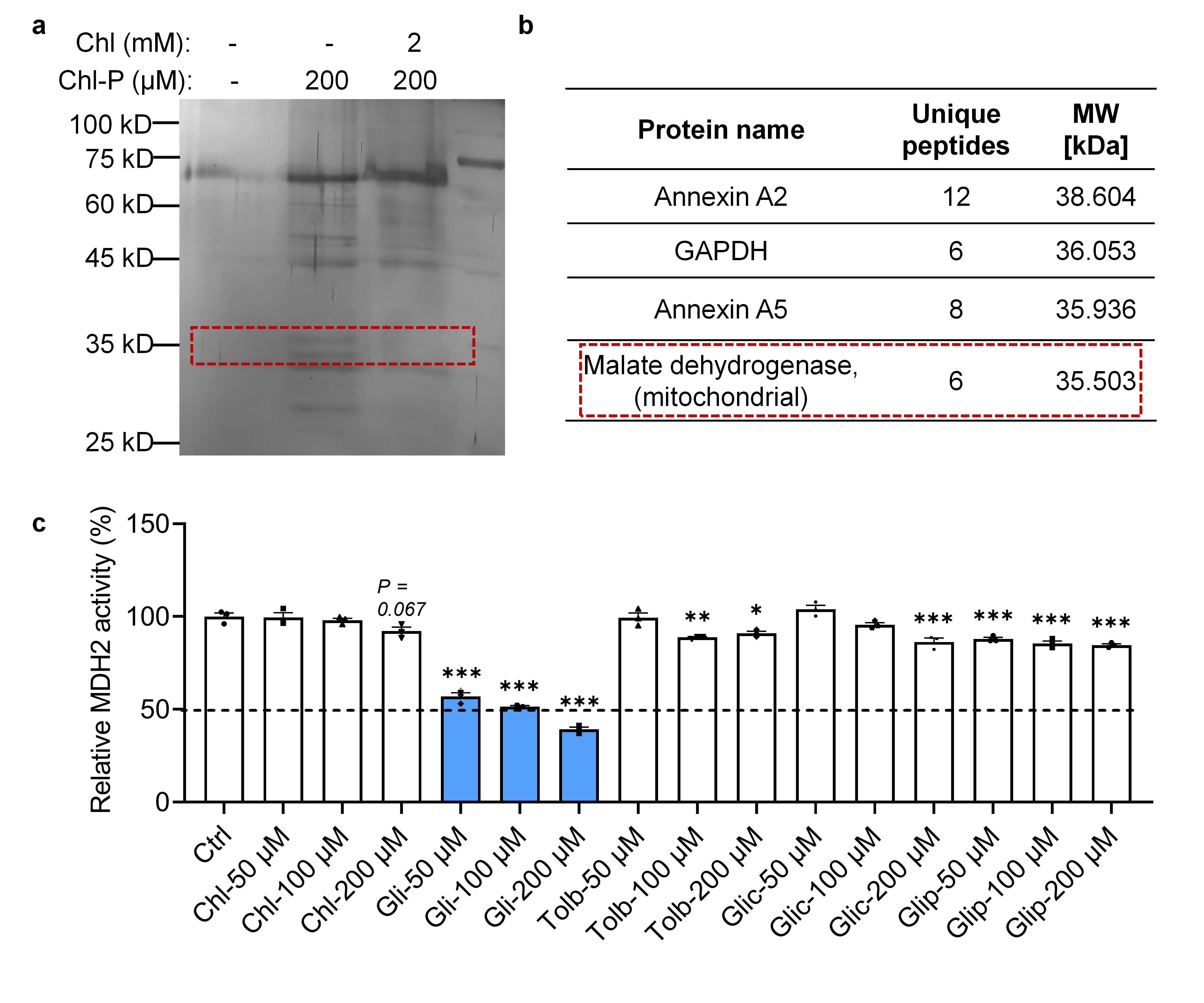


**MDH2 is found as an intervenable target.** **a**, Silver staining of protein labeled by the probe. **b**, Potential protein targets found through ABPP. **c**, MDH2 activity under treatment of sulfonylureas. Chl, chlorpropamide; Gli, glibenclamide; Tolb, tolbutamide; Glic, gliclazide; Glip, glipizide. Error bars represent the standard deviation (± SEM.). The significance of differences (**p* < 0.05, ***p* < 0.01, ****p* < 0.005) was analyzed with Dunnett’s multiple comparisons test.

Figure. S2.

**
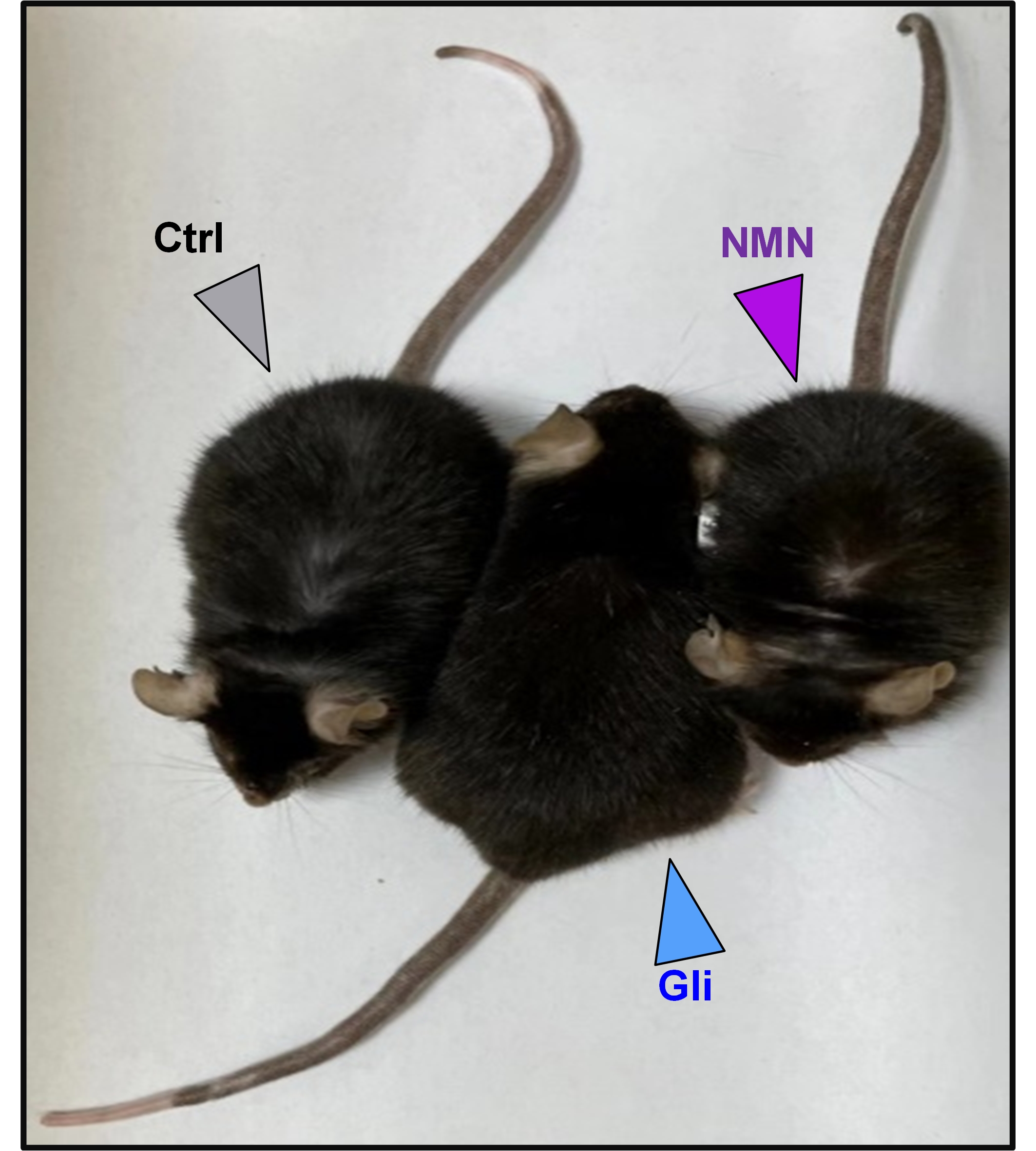
**

**Fur color and coat condition of aged mice.** Mice were treated with solvent (Ctrl), Gli (10 mg/kg) and NMN (500 mg/kg) daily from their 12-month-age and hair phenotypes of mice were observed at 29-month age.

Figure. S3.

**
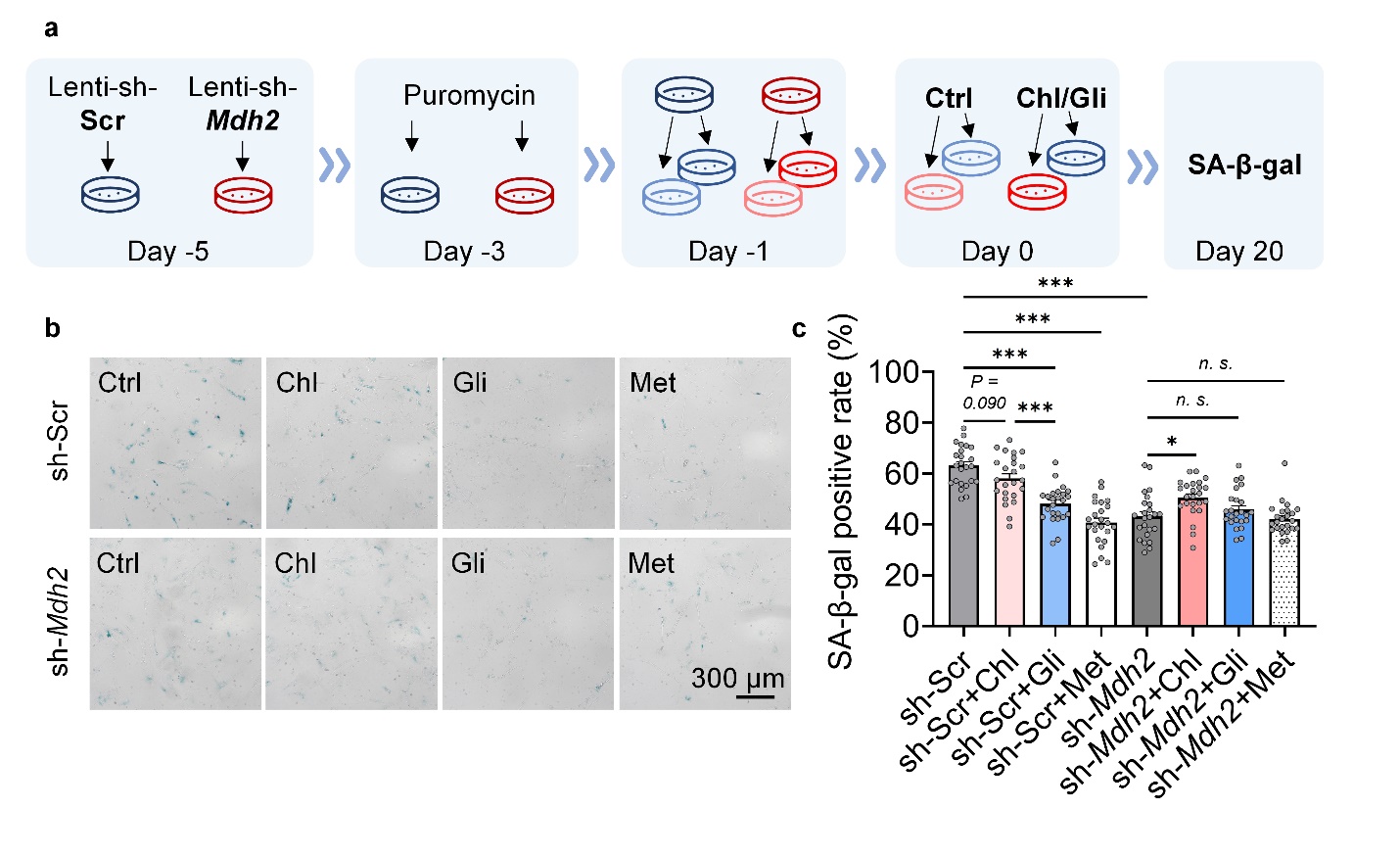
**

**Sulfonylureas relieve cellular senescence in replicative senescent cells dependent on MDH2. a**, Diagram of the experiment. **b**, SA-β-gal staining of sh-Scr/sh-*Mdh2* MRC-5 cells (P40) treated with -/Chl (200 μM)/Gli (100 μM)/Met (100 μM) for 20 days. **c**, Quantification of **b**. Error bars represent the standard deviation (± SEM.). The significance of differences (**p* < 0.05, ***p* < 0.01, ****p* < 0.005, n. s., not significant) was analyzed with Tukey’s multiple comparisons test.

Figure. S4.


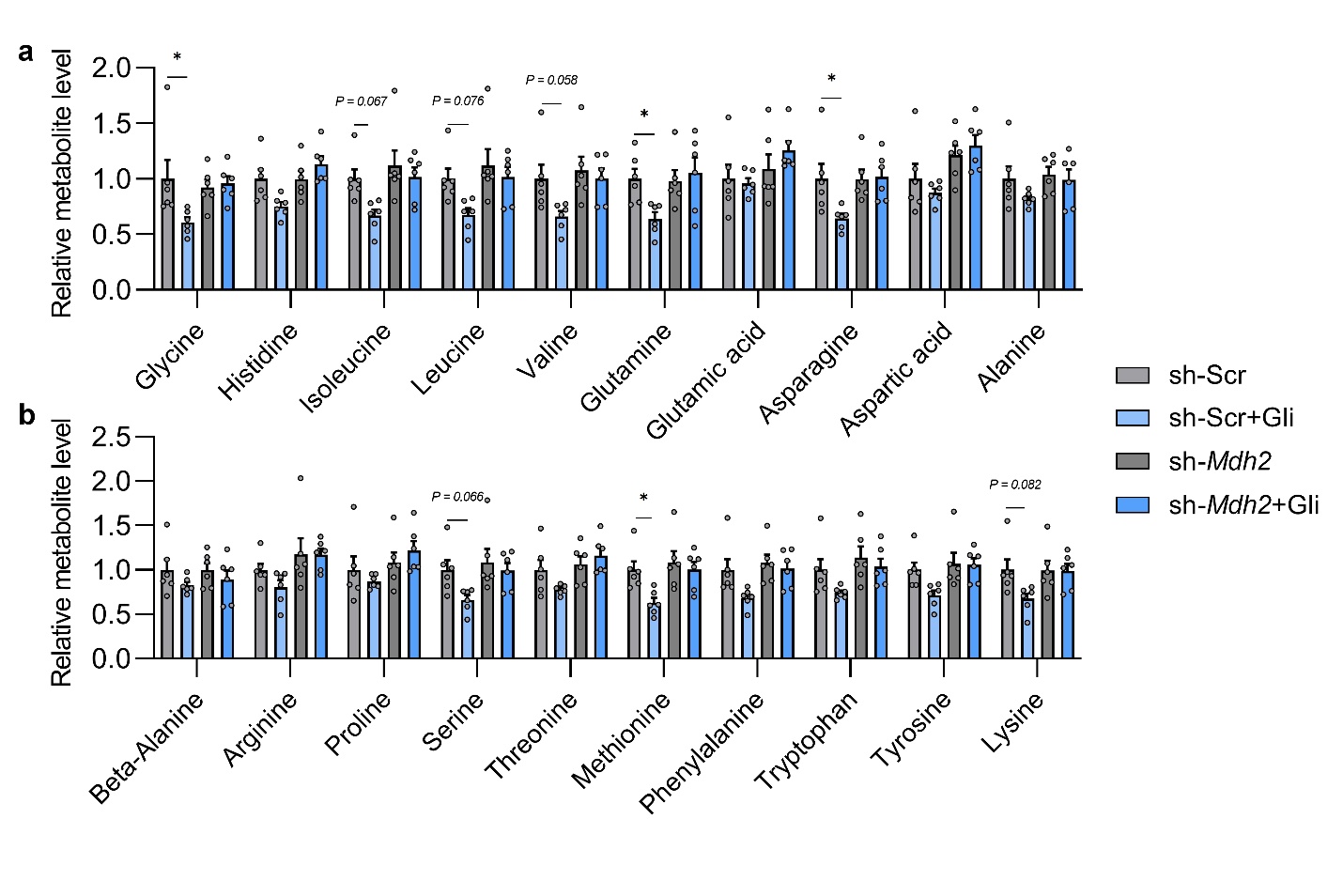


**Metabolic changes under MDH2 inhibition. a**, Relative glycine, histidine, isoleucine, leucin, valine, glutamine, glutamic acid, asparagine, aspartic acid, and alanine level in sh-Scr/sh-*Mdh2* MRC-5 cells (P40) with 2-hour -/Gli (100 μM) treatment. **b**, Relative beta-alanine, arginine, proline, serine, threonine, methionine, phenylalanine, tryptophan, tyrosine, and lysine level in sh-Scr/sh-*Mdh2* MRC-5 cells (P40) with 2-hour -/Gli (100 μM) treatment. Error bars represent the standard deviation (± SEM.). The significance of differences was analyzed with Tukey’s multiple comparisons tests.

Figure. S5.


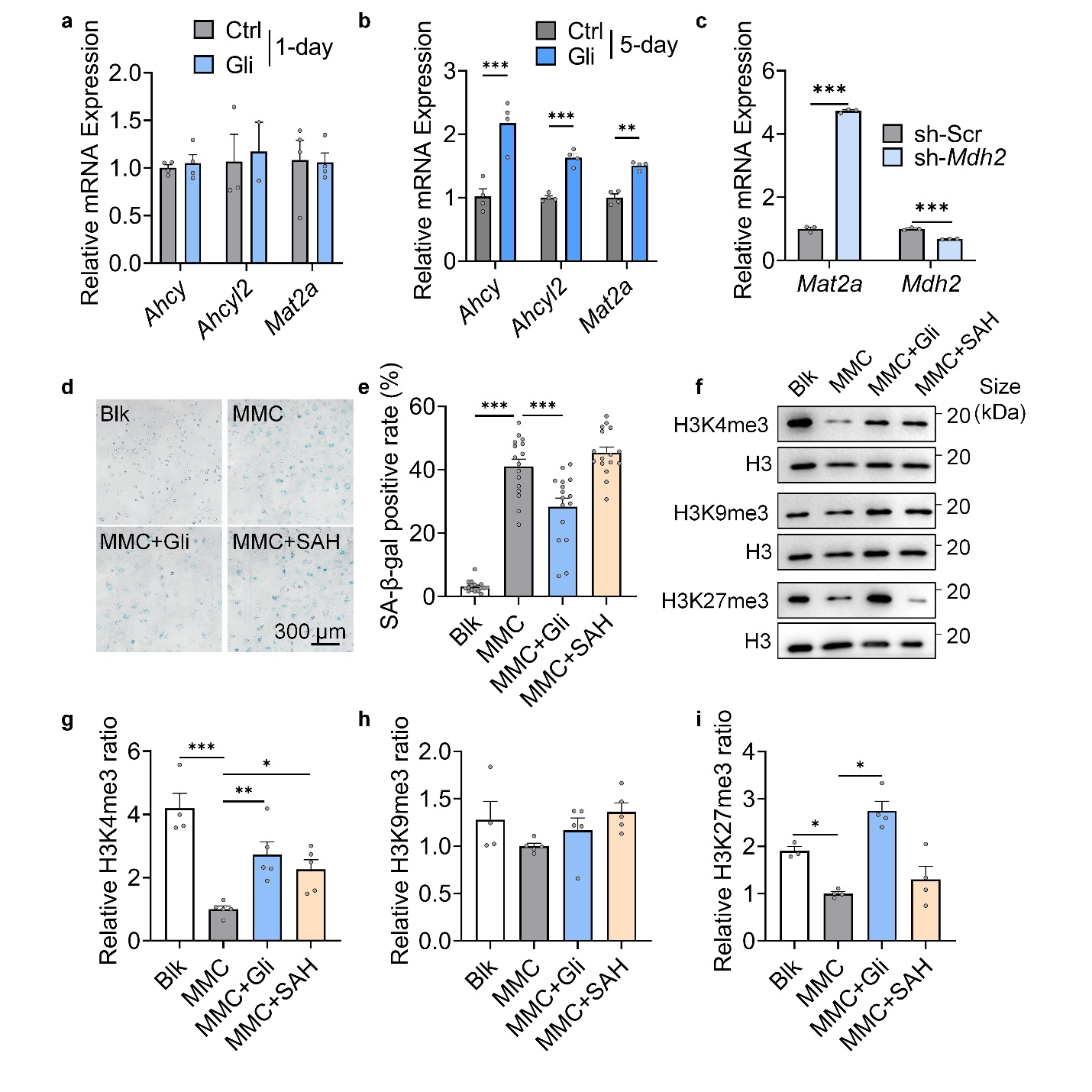


**Methionine cycle flux and histone methylation activated under MDH2 inhibition. a**, Relative mRNA level in MEFs (P7) treated with -/Gli (100 μM) for 1 day. **b**, Relative mRNA level in MEFs (P7) treated with -/Gli (100 μM) for 5 days. **c**, Relative mRNA level in MEFs (P7) transfected with sh-Scr/sh-*Mdh2*. **d**, SA-β-gal staining of Blk/MMC-induced senescent NRK-52E cells treated with -/Gli (100 μM)/SAH (100 μM) for 2 days. **e**, Quantification of **d**. **f**, Relative H3K4me3, H3K9me3, and H3K27me3 level in Blk/MMC-induced senescent NRK-52E cells treated with -/Gli (100 μM)/SAH (100 μM) for 2 days. **g**, Quantification of H3K4me3 grey value ratio in **f**. **h**, Quantification of H3K9me3 grey value ratio in **f**. **i**, Quantification of H3K27me3 grey value ratio in **f**. Error bars represent the standard deviation (± SEM.). The significance of differences in **a**, **b** and **c** were analyzed with Sidak’s multiple comparisons tests, and other significance were analyzed with Tukey’s multiple comparisons tests.

Figure. S6.


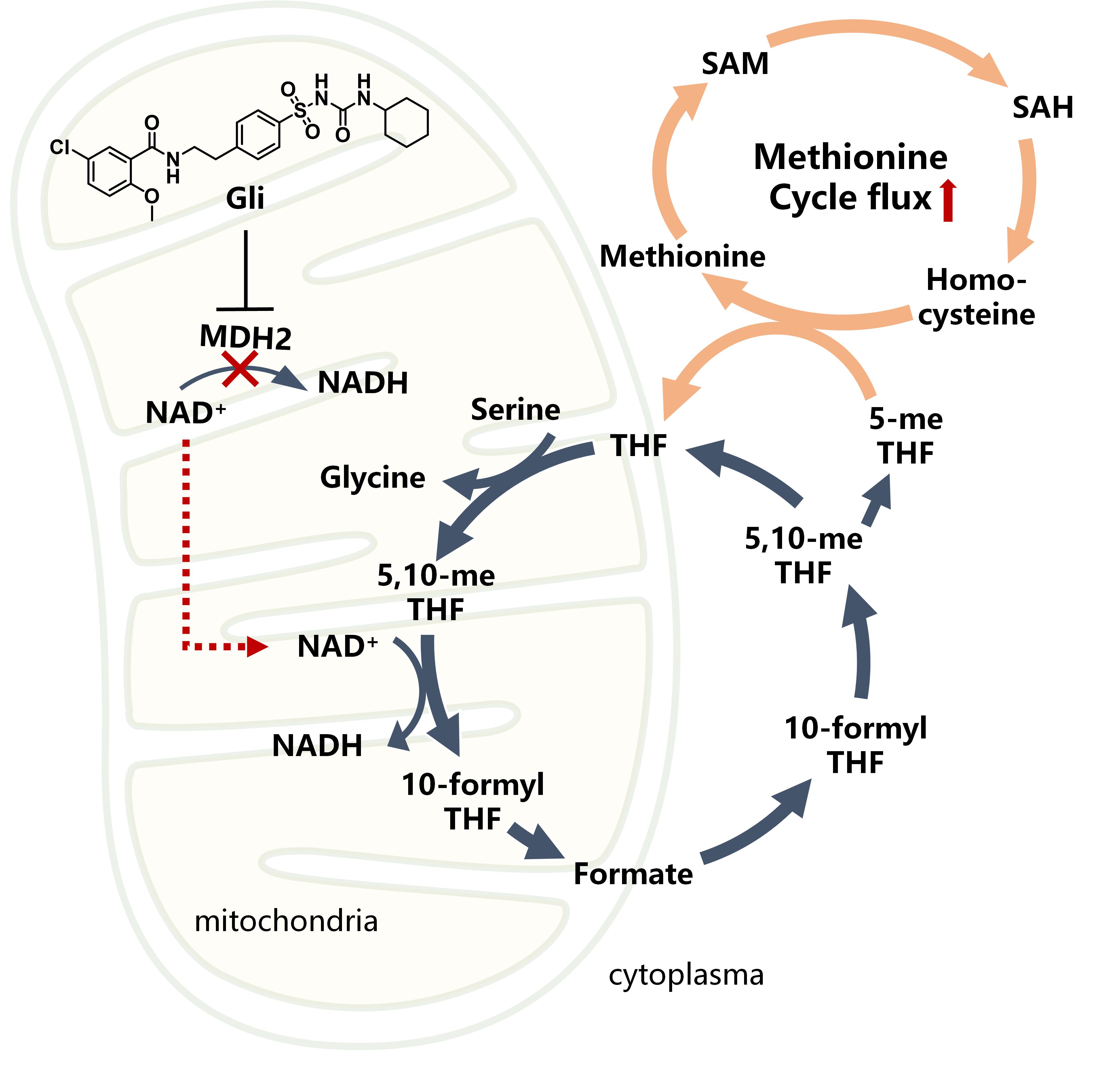


**Diagram of how MDH2 inhibition activates the flux of methionine cycle.** MDH2 inhibition blocks the reduction of mitochondrial NAD^+^, producing more coenzymes for folate-mediated one-carbon metabolism, and offers methyl groups to activate the flux of methionine cycle.

Figure. S7.


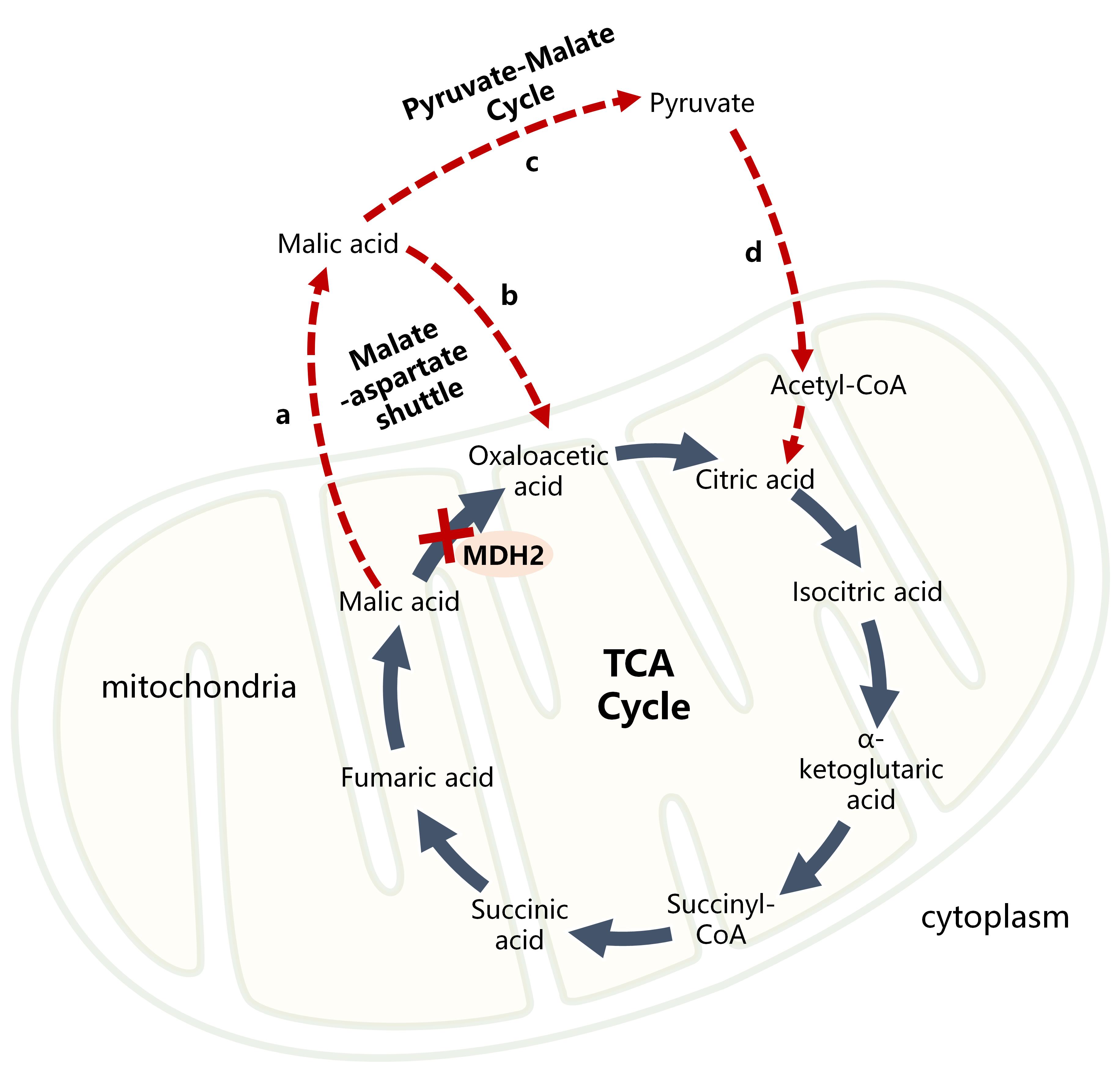


**Diagram of how pyruvate-malate cycle was activated under blockage of MDH2.** Under MDH2 inhibition or knockdown, malic acid is exported from mitochondria to cytoplasm through malate-α-ketoglutarate transporters (a) while imported same equivalent α-ketoglutaric acid. The α-ketoglutaric acid transported into mitochondria during the process stimulate the activity of aspartate-aminotransferases (b), which produces oxaloacetic acid inside mitochondria (Malate-aspartate shuttle). Malic acid transported outside mitochondria can also be decarboxylated by malic enzymes (c) to pyruvate, which enters mitochondria through mitochondrial pyruvate carriers (d), catalyzed to acetyl-CoA, and then condensates with oxaloacetic acid and reentered the TCA cycle (Pyruvate-malate cycle).

Figure. S8.


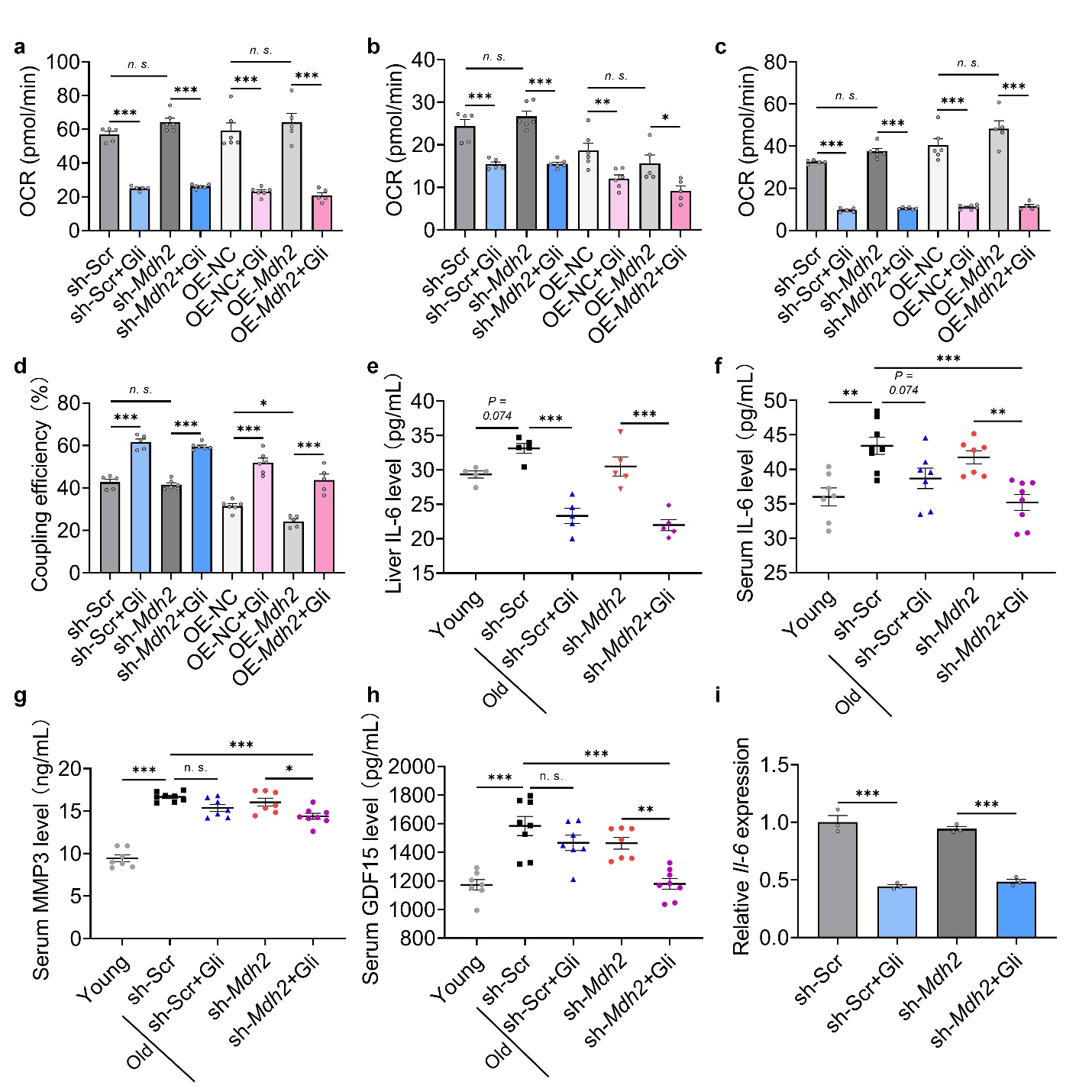


**Effects of Gli independent of MDH2. a**, Basal OCR in sh-Scr/sh-*Mdh2*/OE-NC/OE-*Mdh2* MEFs treated with -/Gli (100 μM) for 2 hours. **b**, ATP-relative OCR in sh-Scr/sh-*Mdh2*/OE-NC/OE-*Mdh2* MEFs treated with -/Gli (100 μM) for 2 hours. **c**, Proton leak related OCR in sh-Scr/sh-*Mdh2*/OE-NC/OE-*Mdh2* MEFs treated with -/Gli (100 μM) for 2 hours. **d**, Respiration efficiency in sh-Scr/sh-*Mdh2*/OE-NC/OE-*Mdh2* MEFs treated with -/Gli (100 μM) for 2 hours. **e**, Liver IL-6 level of mice in different groups. **f**, Serum IL-6 level of mice in different groups. **g**, Serum MMP3 level of mice in different groups. **h**, Serum GDF15 level of mice in different groups. **i**, Relative *Il-6* expression in sh-Scr/sh-*Mdh2* MEFs treated with -/Gli (100 μM) for 1 day. Sample size for **e**: n = 5 for all groups. Sample size for **f**-**h**: Young, n = 7; sh-Scr, n= 8; sh-Scr+Gli (10 mg/kg), n = 7; sh-*Mdh2*, n = 7 sh-*Mdh2*+Gli (10 mg/kg), n = 8. Error bars represent the standard deviation (± SEM.). The significance of differences (**p* < 0.05, ***p* < 0.01, ****p* < 0.005) were analyzed with Tukey’s multiple comparisons tests.

Table S3.

| **Metabolite Name** | **SG vs SC** | **RG vs RC** |
| --- | --- | --- |
| 6-Phosphogluconic acid (6-PGA) | **↓** | **-** |
| Asparagine | **↓** | **-** |
| Malic acid | **↑** | **-** |
| Taurochenodeoxycholic acid (TCDCA) | **↑** | **-** |
| Carnitine | **↓** | **-** |
| Leucine | **↓** | **-** |
| Lysine | **↓** | **-** |
| Galactitol | **↓** | **-** |
| Valine | **↓** | **-** |
| S-Adenosylhomocysteine (SAH) | **↓** | **-** |
| Serine | **↓** | **-** |

**Tendency of 11 metabolites with easy accessibility of 26 MDH2-dependent Gli-regulated differential metabolites.** SC: sh-Scr; SG: sh-Scr+Gli (100 μM); RC: sh-*Mdh2*; RG: sh-*Mdh2*+Gli (100 μM).

Data S1.

Synthesis and structural characterization of chemical probes. See file “Supplementary Data 1”.

Data S2.

Original films of western blots. See file “Supplementary Data 2”.

Table S1.

Differential metabolites in MRC-5 cells treated with Gli for 2 or 24 hours. See file “Supplementary Table 1”.

Table S2.

Differential metabolites in sh-Scr/sh-*Mdh2* MRC-5 cells treated with Gli for 2 hours. See file “Supplementary Table 2”.
