# Supplementary material for: Glibenclamide targets MDH2 to relieve aging phenotypes through metabolism-regulated epigenetic modification
Source: Signal Transduct Target Ther. 2025 Feb 17;10:67. doi: 10.1038/s41392-025-02157-3 (PMC11833132; doi:10.1038/s41392-025-02157-3)
Supplement: Supplementary file 4 — Supplementary Data 1 [file 41392_2025_2157_MOESM4_ESM.docx]

Supplementary Data 1

Glibenclamide targets MDH2 to relieve aging phenotypes through metabolism-regulated epigenetic modification

Zhifan Mao^2†^, Wenwen Liu^1†^, Rong Zou^2†^, Ling Sun^1†^, Shuman Huang^2^, Lingyu Wu^1^, Liru Chen^1^, Jiale Wu^1^, Shijie Lu^2^, Zhouzhi Song^2^, Xie Li^2^, Yunyuan Huang^3^, Yong Rao^1^, Yi-You Huang^1^, Baoli Li^1*^, Zelan Hu^2*^, Jian Li^1,2,4*^

Correspondence to: jianli@ecust.edu.cn (J. L.); huzelan@ecust.edu.cn (Z. H.); baolili@hainanu.edu.cn (B. L.)

Supplementary Text

Materials and instruments for synthesis

All chemicals, biological reagents, and solvents were purchased from commercial suppliers and were used without further purification. Suppliers of the materials including Sigma-Aldrich, Titan, Yeason, et al. The ^1^H NMR and ^13^C NMR spectra were recorded on a Bruker AMX-400 and AMX-600 spectrometer. Chemical shifts were reported in parts per million (ppm, δ) downfield from tetramethylsilane (TMS). Proton coupling patterns were described as singlet (s), doublet (d), triplet (t), quartet (q), multiplet (m) and doublet of doublets (dd). High resolution mass spectrometry (HRMS) data were obtained by electrospray ionization (ESI) using a Waters LCT Premier XE spectrometer.

Synthesis of Chl-P

Scheme 1


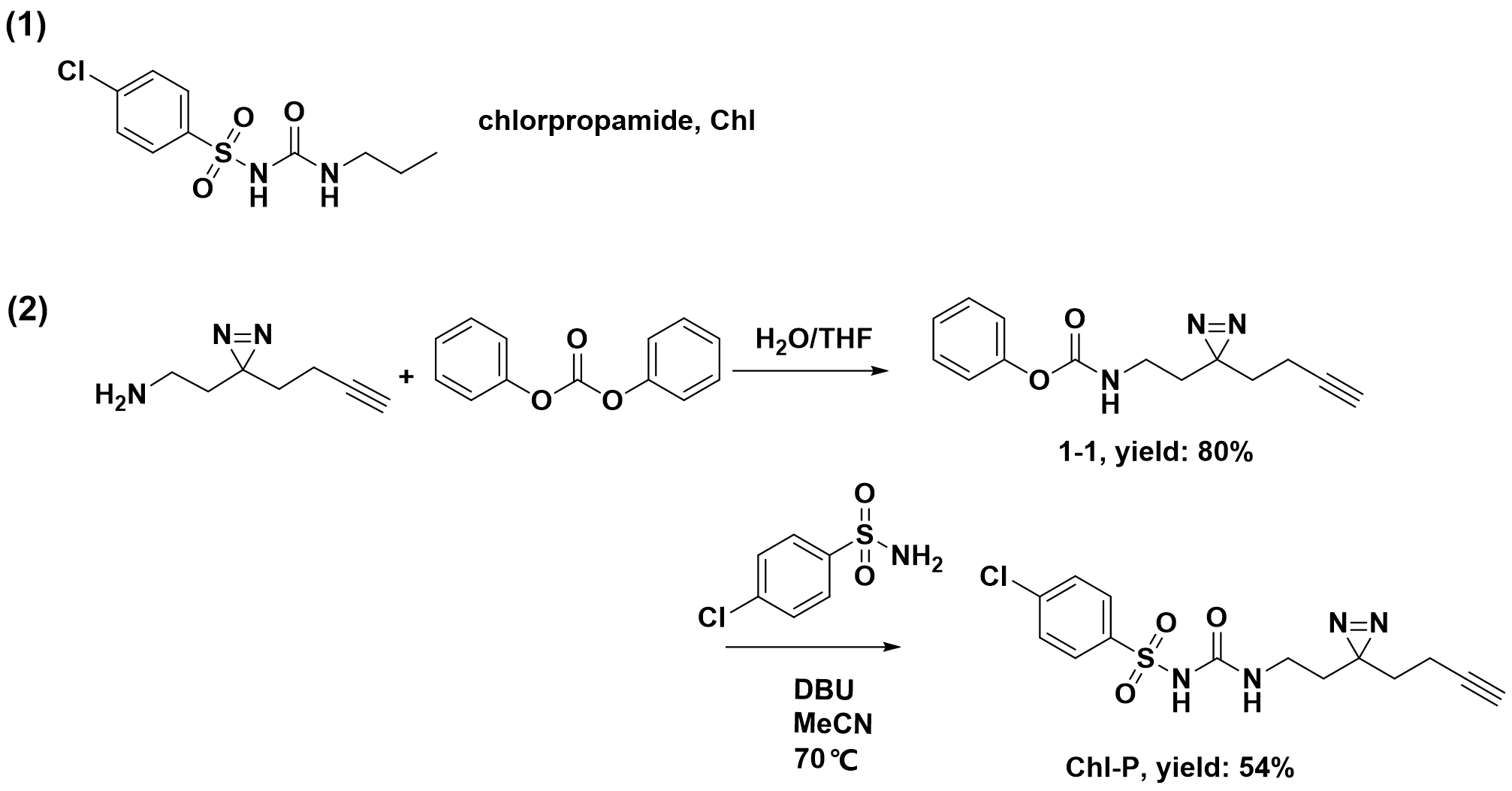


**Phenyl(2-(3-(but-3-yn-1-yl)-3H-diazirin-yl)ethyl)carbamate (1-1)**: Under nitrogen, 3-aminoethyl-3- (butyl-3-alkynyl) bisacridine (1 eq, 100 mg) (CAS: 1450752-97-2) and (1 eq, 156.15 mg) (CAS: 102-09-0) were diluted in a mixed solution of water (1.75 mL):tetrahydrofuran (194 μL) (9:1), and stirred for 16 hours under room temperature. The crude product was dissolved in ethyl acetate, extracted sequentially with 10% NaOH and saturated NaCl solution, dried by Na_2_SO_4_, and purified by silica column chromatography using PE:EA to yield **1-1** (150 mg, colorless oil, yield: 80%). **^1^H NMR (400 MHz, Chloroform-*d*)**: δ 7.29 (t, *J* = 7.7 Hz, 2H), 7.13 (t, *J* = 7.4 Hz, 1H), 7.06 (d, *J* = 8.0 Hz, 2H), 5.06 (s, 1H), 3.08 (q, *J* = 6.5 Hz, 2H), 2.03 – 1.93 (m, 3H), 1.70 (t, *J* = 6.7 Hz, 2H), 1.63 (t, *J* = 7.1 Hz, 2H).

**N-((2-(3-(but-3-yn-1-yl)-3H-diazirin-3-yl)ethyl)carbamoyl)-4-chlorobenzene-sulfonamide (Chl-P)**: Under nitrogen, compound **1-1** (1 eq, 160 mg), 4-chlorobenzenesulfonamide (1 eq, 107 mg) (CAS: 98-64-6), and DBU (3 eq, 127 μL) (CAS: 6674-22-2) was dissolved in ultra dry acetonitrile (4.3 mL). Mixture was stirred and reflux at 70 ℃ for 4 hours. Solvent was removed through rotary evaporation, and the crude product was dissolved in ethyl acetate and extracted through 0.1N HCl and saturated NaCl solution, dried by anhydrous Na_2_SO_4_, purified by silica column chromatography using DCM:MeOH to yield **Chl-P** (119 mg, white solid, yield: 54%, mp 74-76 ℃, purity: 98%). **^1^H NMR (400 MHz, Chloroform-*d*)**: δ 8.34 (s, 1H), 7.90 (d, *J* = 8.3 Hz, 2H), 7.54 (d, *J* = 8.3 Hz, 2H), 6.56 (t, *J* = 5.8 Hz, 1H), 3.11 (q, *J* = 6.4 Hz, 2H), 2.05 – 1.96 (m, 3H), 1.71 (t, *J* = 6.6 Hz, 2H), 1.62 (t, *J* = 7.1 Hz, 2H). **^13^C NMR (100 MHz, Chloroform-*d*)**: *δ* 151.76, 140.56, 137.99, 129.71, 128.55, 82.47, 69.61, 35.35, 32.49, 32.08, 26.63, 13.18. **ESI-HRMS** [M+Na]^+^ calcd for C_14_H_15_ClN_4_O_3_SNa^+^: 377.0446, found: 377.0449.

Synthesis of Gli-P

Scheme 2


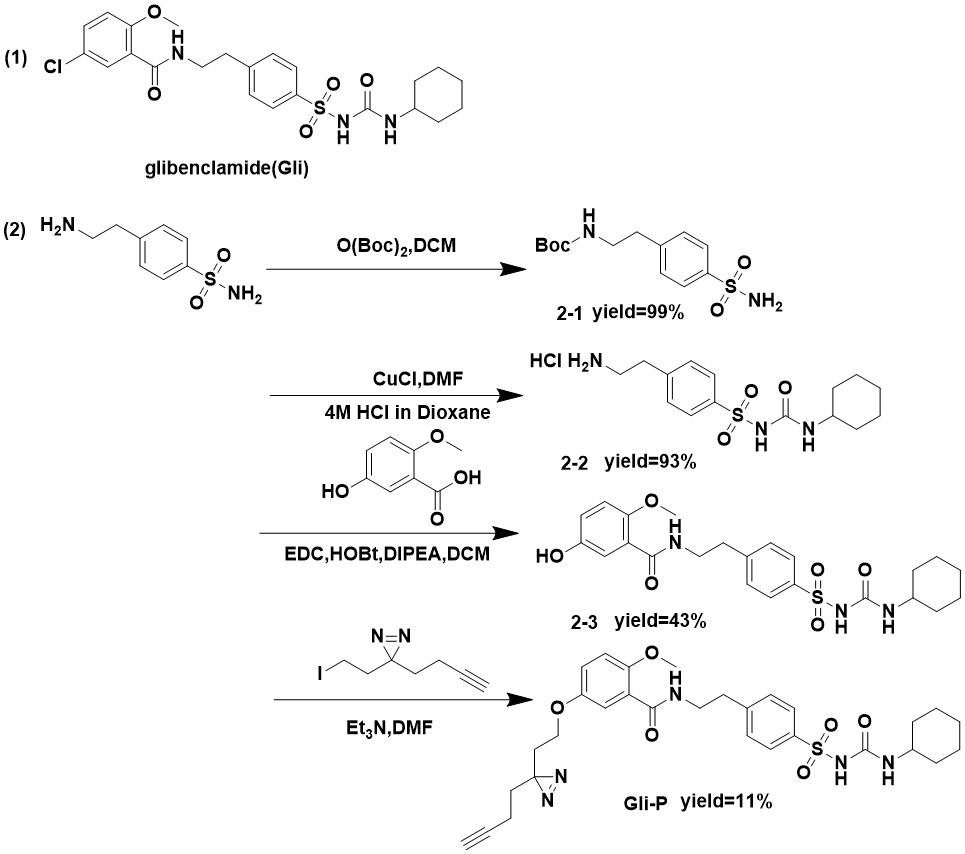


***tert*-butyl(4-sulfamoylphenethyl)carbamate (2-1):** 4-(2-aminoethyl) benzene-sulfonamide (1 eq, 1 g,) (CAS:35303-76-5) and tert-butyldicarbonate (1 eq, 1.09 g) (CAS: 24424-99-5) was dissolved in ultra dry dichloromethane (25 mL). The reaction mixture was stirred at room temperature for 5 h. The solvent was evaporated under reduced pressure to yield **2-1** (1.49g, white solid, yield: 99%). **^1^H NMR (400 MHz, Methanol-*d*_4_)**: δ 7.82 (d, *J* = 8.1 Hz, 2H), 7.39 (d, *J* = 8.0 Hz, 2H), 3.31 – 3.26 (m, 2H), 2.84 (t, *J* = 7.2 Hz, 2H), 1.41 (s, 9H).

**4-(2-Aminoethyl)-N-(cyclohexylcarbamoyl)benzenesulfonamide hydrochloride (2-2**): Under nitrogen, compound **2-1** (1 eq, 1.49 g) and CuCl (0.1 eq, 49 mg) (CAS: 7758-89-6) was dissolved in ultra dry *N*, *N*-Dimethylformamide (35 mL), and then isocyanatocyclohexane (2 eq, 1.24 g) (CAS: 3173-53-3) was added to the reaction slowly. The resulting reaction mixture was stirred at room temperature under argon for 1 d. The solution was poured into crushed ice water and quenched, and 1M hydrochloric acid is added dropwise until pH value was around 3. White solids will be precipitated in the solution. Suction filtration separates solids and liquids. Collected the solids and then 4 M HCl in dioxane (10 eq, 12.4 mL) was added. The new reaction mixture was stirred at room temperature for 3 h. The solvent was evaporated under reduced pressure to yield **2-2** (1.67 g, beige solid, yield: 93%). **^1^H NMR (400 MHz, Methanol-*d*_4_)**: δ 8.03 – 7.92 (m, 2H), 7.51 (d, *J* = 8.0 Hz, 2H), 3.45 – 3.38 (m, 1H), 3.24 (t, *J* = 7.7 Hz, 2H), 3.07 (t, *J* = 7.6 Hz, 2H), 1.88 – 1.52 (m, 7H), 1.33 – 1.16 (m, 6H).

**N-(4-(N-(cyclohexylcarbamoyl)sulfamoyl)phenethyl)-5-hydroxy-2-methoxy-benzamide (2-3):** 5-Hydroxy-2-methoxybenzoic acid (1 eq, 30 mg), HOBt (1 eq, 27 mg) and EDCI (1 eq, 34 mg) was dissolved in ultra dry dichloromethane (3 mL), and the reaction was stirred for 10 min, then compound **2-2** (1 eq, 67 mg) and DIPEA(3 eq, 69 mg) was added to the reaction and stirred at room temperature for 2 h. Then extracted with DCM. The organic layers were combined, washed with brine, dried by Na_2_SO_4_, and purified by silica column chromatography using DCM:MeOH to yield **2-3** (38 mg, white solid, yield: 43%). **^1^H NMR (400 MHz, Methanol-*d*_4_)**: *δ* 8.41 (s, 1H), 7.94 – 7.90 (m, 2H), 7.50 (d, *J* = 8.2 Hz, 2H), 7.34 (d, *J* = 3.0 Hz, 1H), 6.96 – 6.86 (m, 2H), 3.74 (s, 3H), 3.73 – 3.68 (m, 2H), 3.45 – 3.38 (m, 1H), 3.03 (t, *J* = 6.9 Hz, 2H), 1.79 – 1.55 (m, 5H), 1.34 – 1.15 (m, 5H).

**5-(2-(3-(but-3-yn-1-yl)-3H-diazirin-3-yl)ethoxy)-N-(4-(N-(cyclohexylcarbamoyl)-sulfamoyl)phenethyl)-2-methoxybenzamide (Gli-P):** Under nitrogen, compound **2-3** (30 mg, 1.0 eq.) and Et_3_N (19 mg, 0.18 mmol, 3.0 eq.) was dissolved in ultra dry *N*, *N*-Dimethylformamide (3 mL), then added 3-(but-3-yn-1-yl)-3-(2-iodoethyl)-3H-diazirine (1.1 eq) slowly to the reaction under argon and keep protect from light. The reaction was stirred at 50 ℃ for 12 h. Then extracted with ethyl acetate. The organic layers were combined, washed with brine, dried by Na_2_SO_4_, and purified by silica column chromatography using PE:EA to yield **Gli-P** (4 mg, white solid, yield: 11%, mp 154-155 ℃, purity: 98%). **^1^H NMR (400 MHz, Methanol-*d*_4_)**: *δ* 7.81 (d, *J* = 8.4 Hz, 2H), 7.55 (d, *J* = 8.4 Hz, 2H), 7.35 (d, *J* = 2.8 Hz, 1H), 6.96 – 6.87 (m, 2H), 3.76 (s, 3H), 3.70 (q, *J* = 6.0, 5.2 Hz, 2H), 3.66 – 3.60 (m, 2H), 3.53 (s, 1H), 3.04 (t, *J* = 6.8 Hz, 2H), 2.27 (t, *J* = 2.8 Hz, 1H), 2.01 (td, *J* = 7.6, 2.4 Hz, 2H), 1.82 (d, *J* = 11.6 Hz, 2H), 1.69 (d, *J* = 13.6 Hz, 2H), 1.62 (t, *J* = 7.6 Hz, 4H), 1.29 (d, *J* = 4.0 Hz, 4H), 0.90 (t, *J* = 6.0 Hz, 2H). **^13^C NMR (100 MHz, Methanol-*d*_4_)**: *δ* 166.57, 151.68, 151.13, 151.01, 146.63, 136.74, 129.91, 126.95, 121.90, 119.19, 116.70, 112.98, 82.17, 69.02, 55.54, 49.96, 40.87, 40.14, 34.83, 33.30, 32.21, 31.56, 29.37, 26.07, 25.09, 24.30, 12.42. **ESI-HRMS** [M+H]^+^ calcd for C_30_H_38_N_5_O_6_S^+^: 596.2537, found: 596.2539. [M+Na]^+^ calcd for C_30_H_37_N_5_O_6_SNa^+^: 618.2357, found: 618.2359.

**Spectra of chemical probes and the intermediates**

**Figure. S9.**


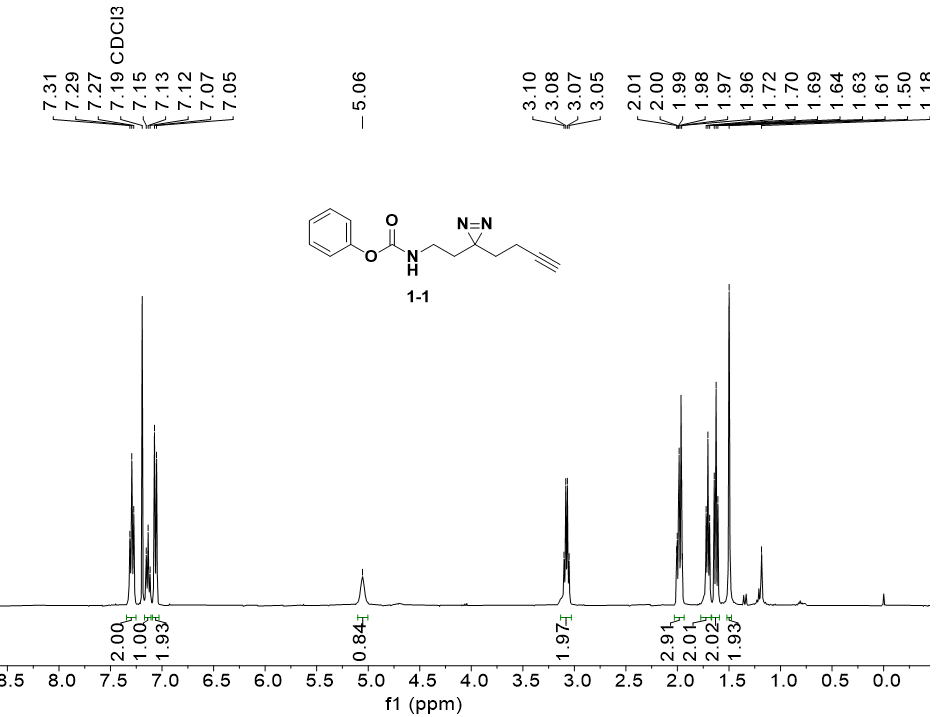


^1^H NMR spectrum of **1-1** in CDCl_3_

**Figure. S10.**


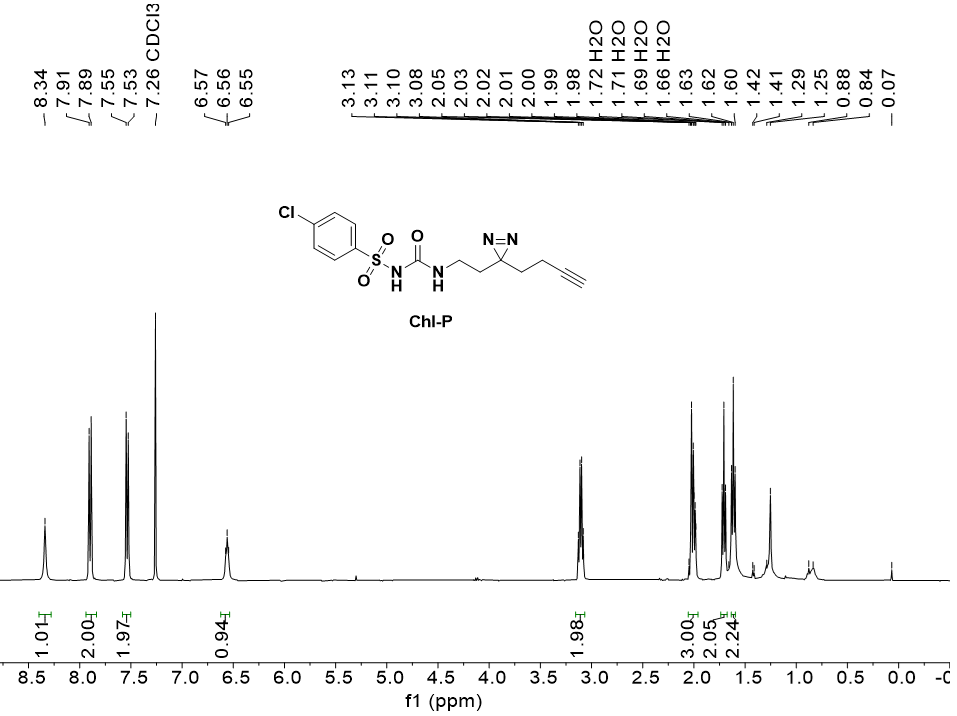


^1^H NMR spectrum of **Chl-P** in CDCl_3_

**Figure. S11.**


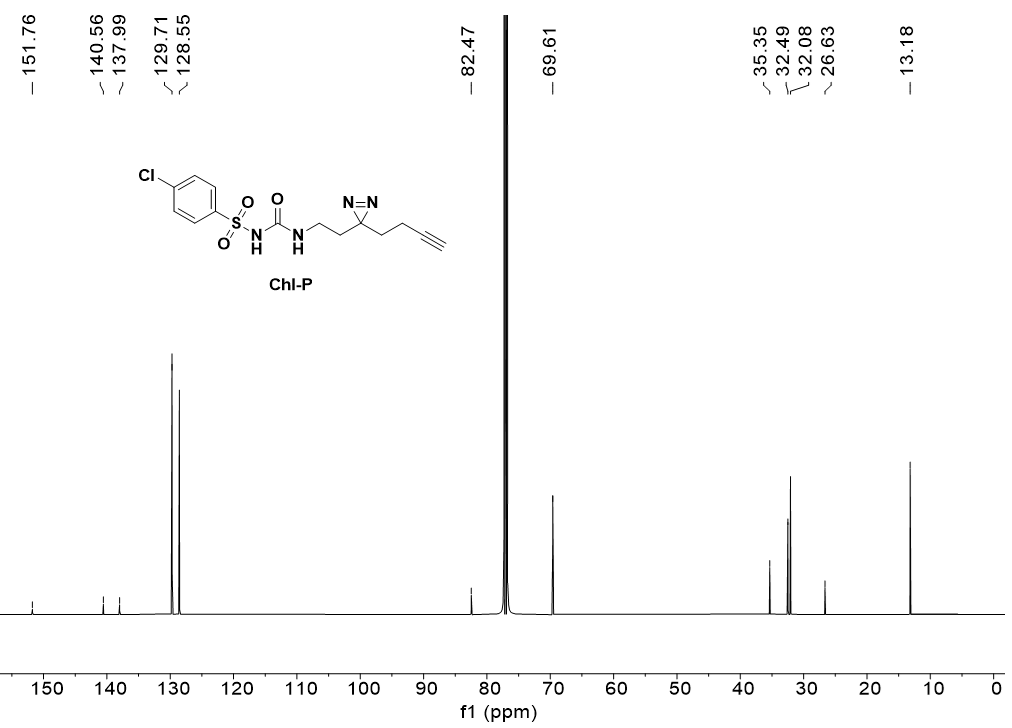


^13^C NMR spectrum of **Chl-P** in CDCl_3_

**Figure. S12.**

**
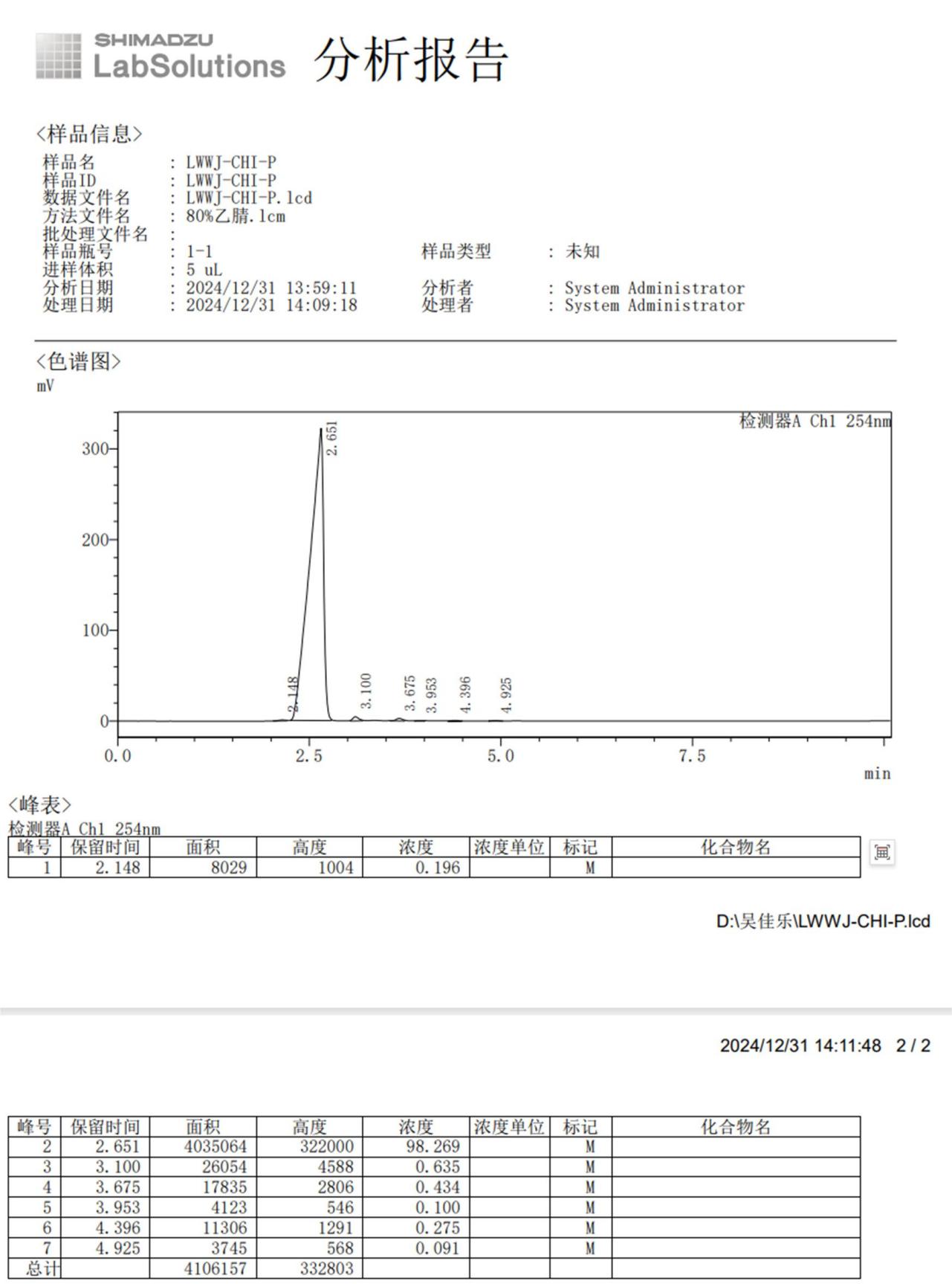
**

HPLC spectrum of **Chl-P**

**Figure. S13.**

**
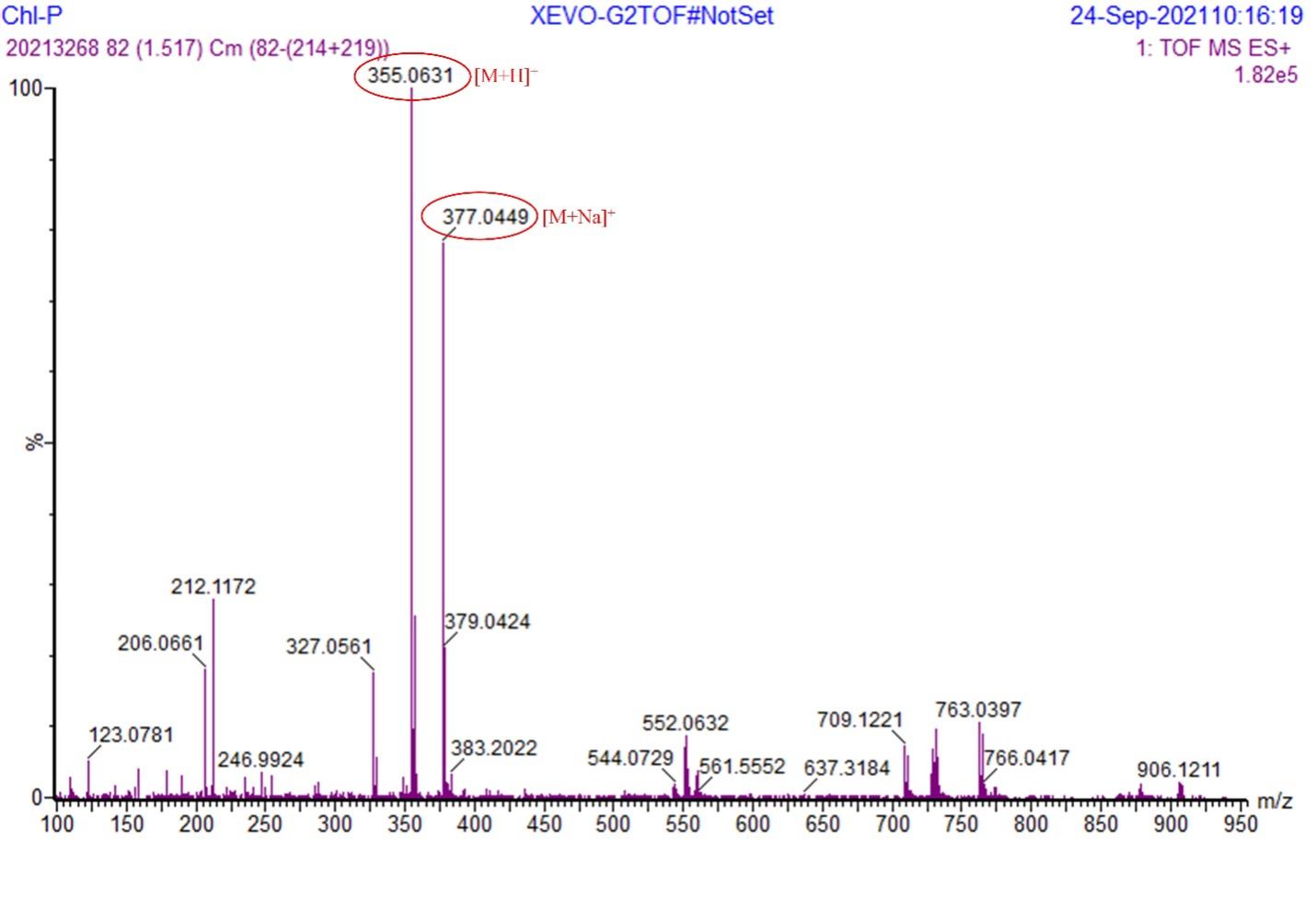
**

HRMS spectrum of **Chl-P**

**Figure. S14.**

^
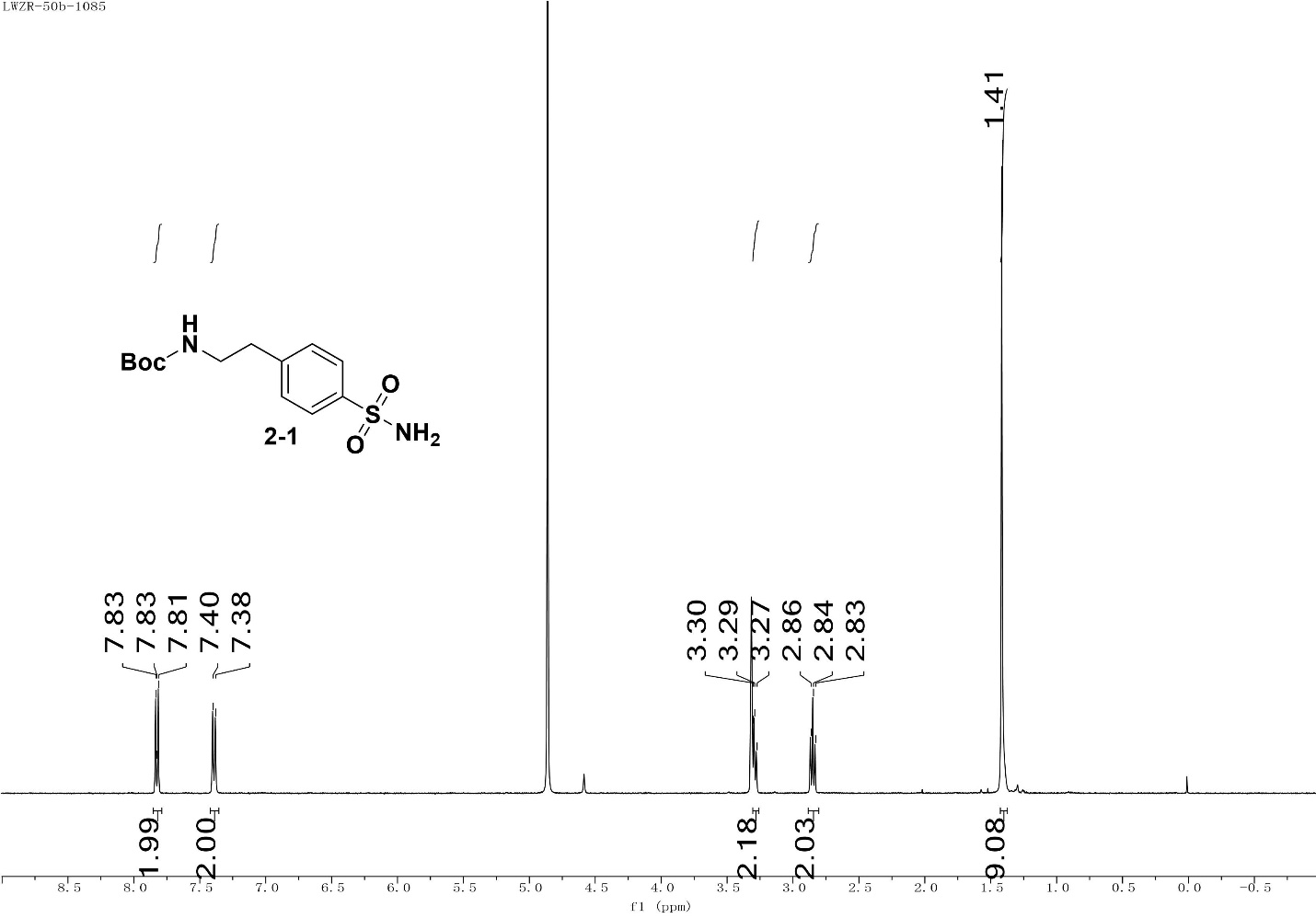
1^H NMR spectrum of **2-1** in MeOD

**Figure. S15.**

^
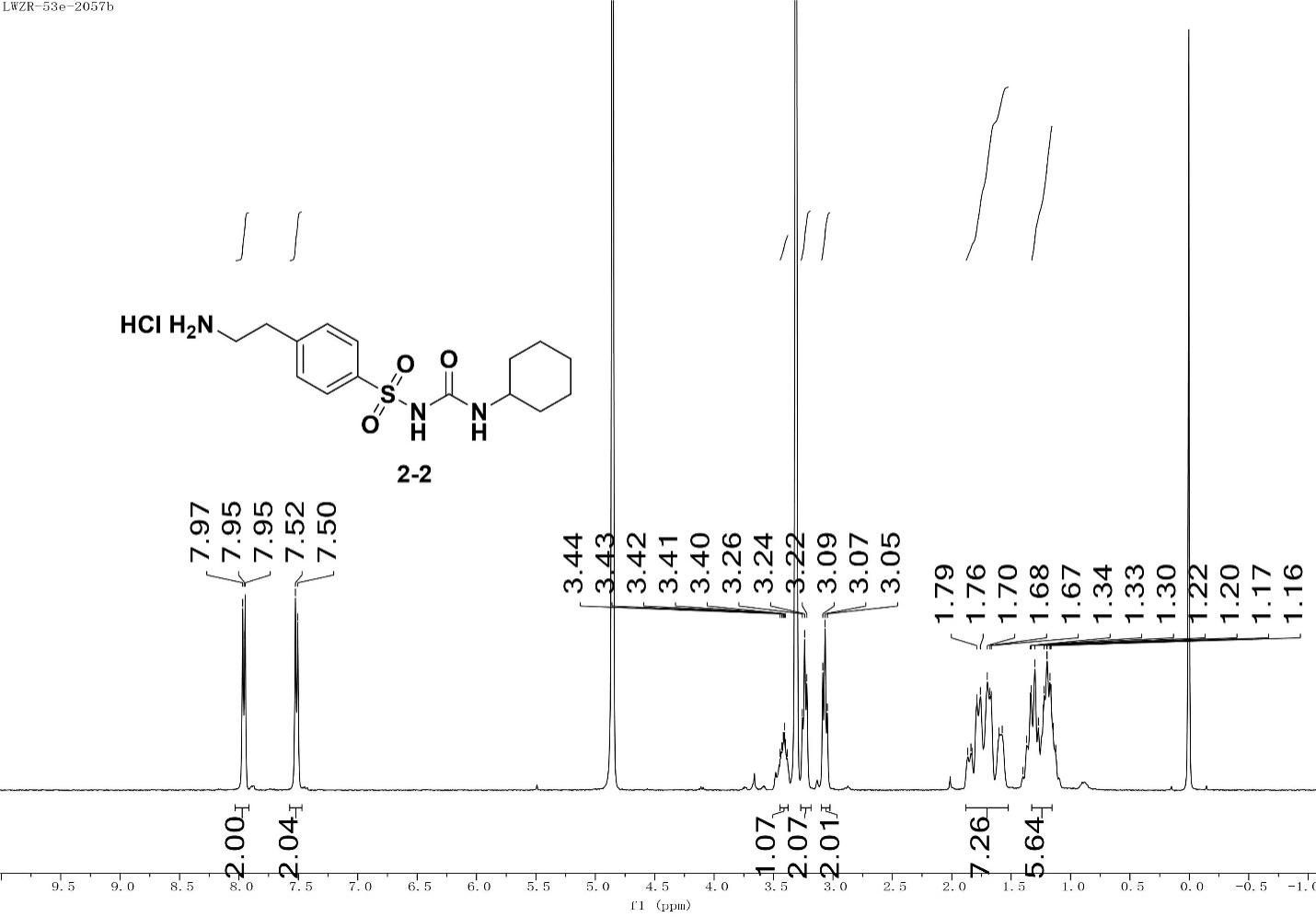
1^H NMR spectrum of **2-2** in MeOD

**Figure. S16.**

^
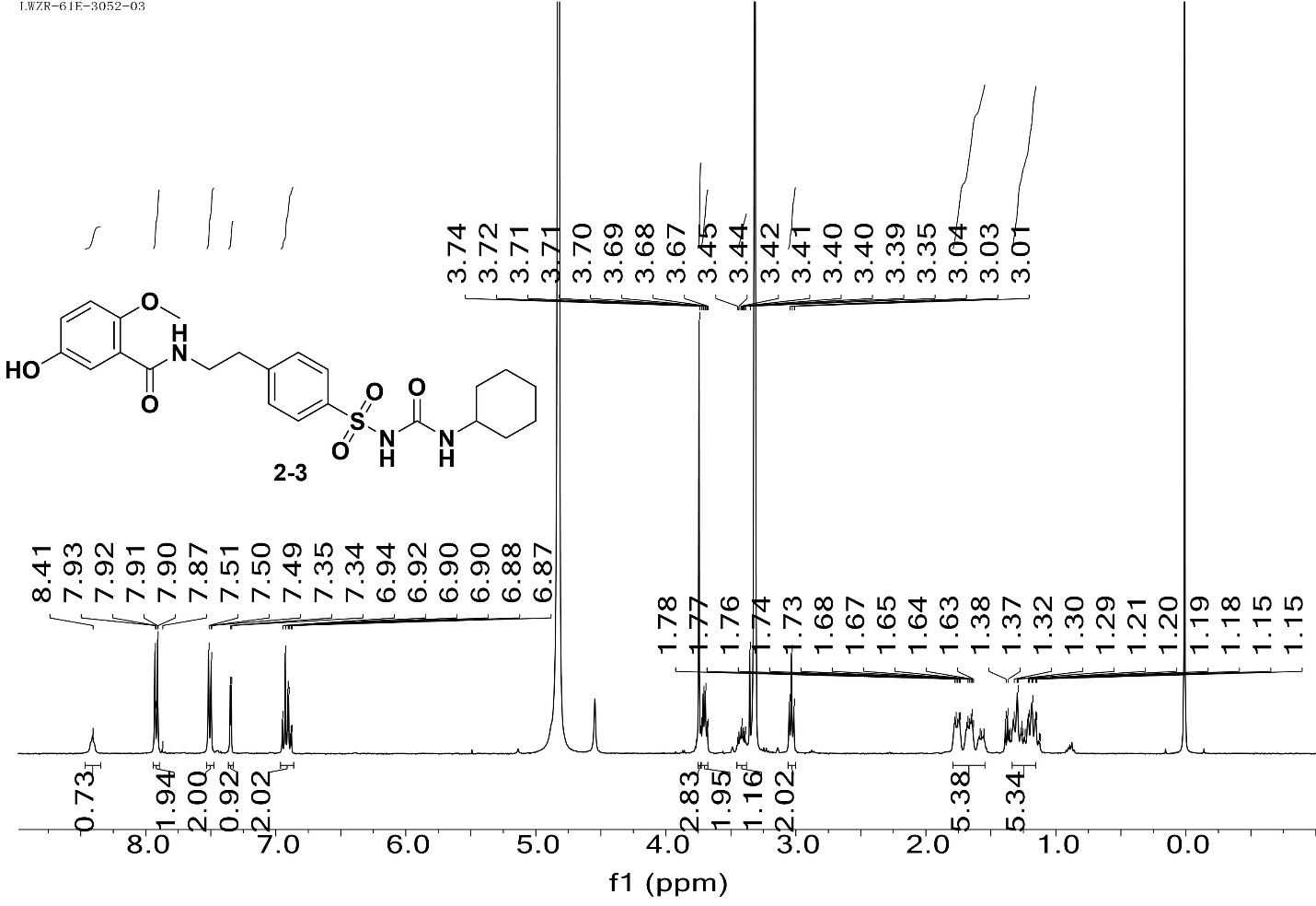
1^H NMR spectrum of **2-3** in MeOD

**Figure. S17.**

^
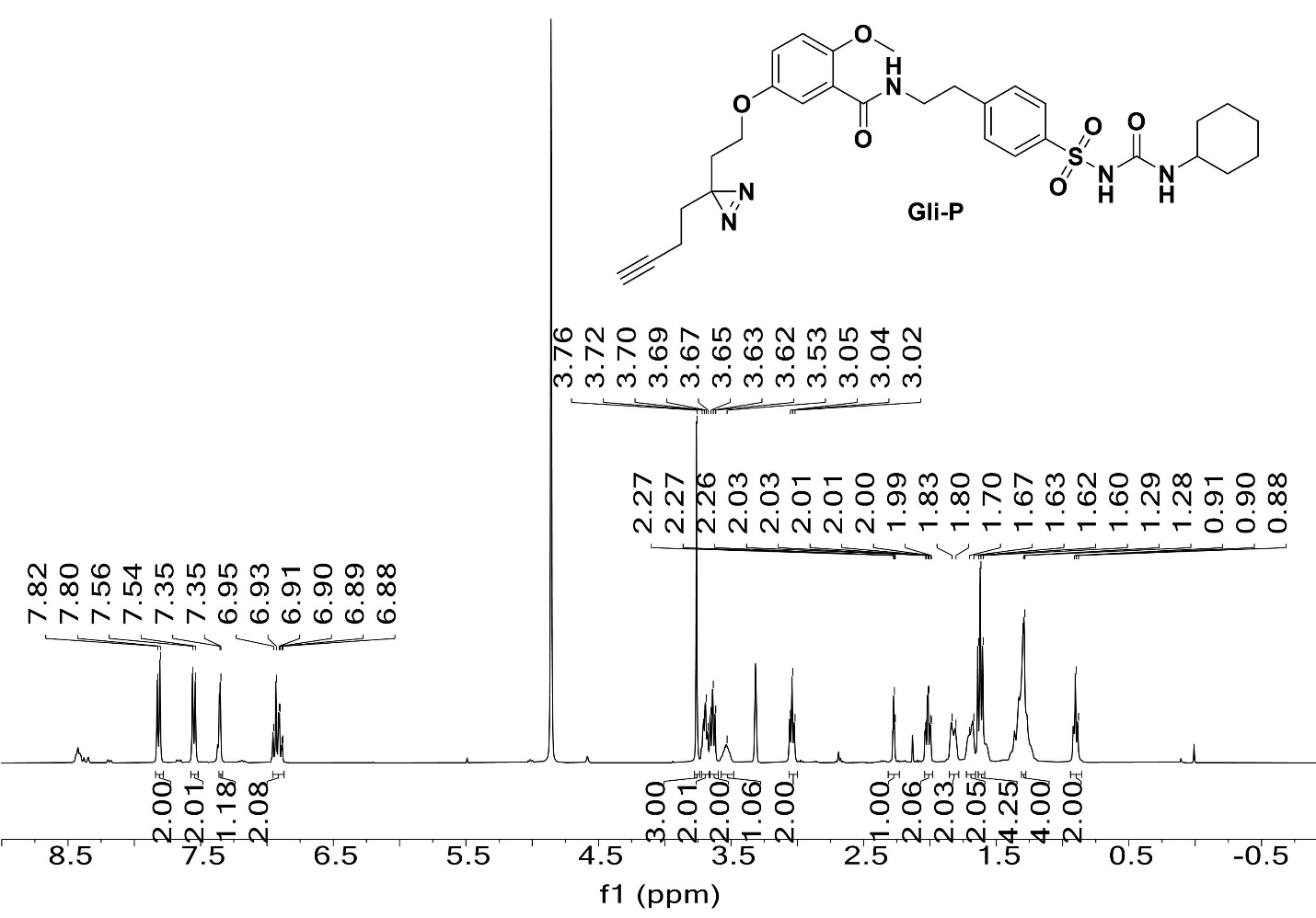
1^H NMR spectrum of **Gli-P** in MeOD

**Figure. S18.**


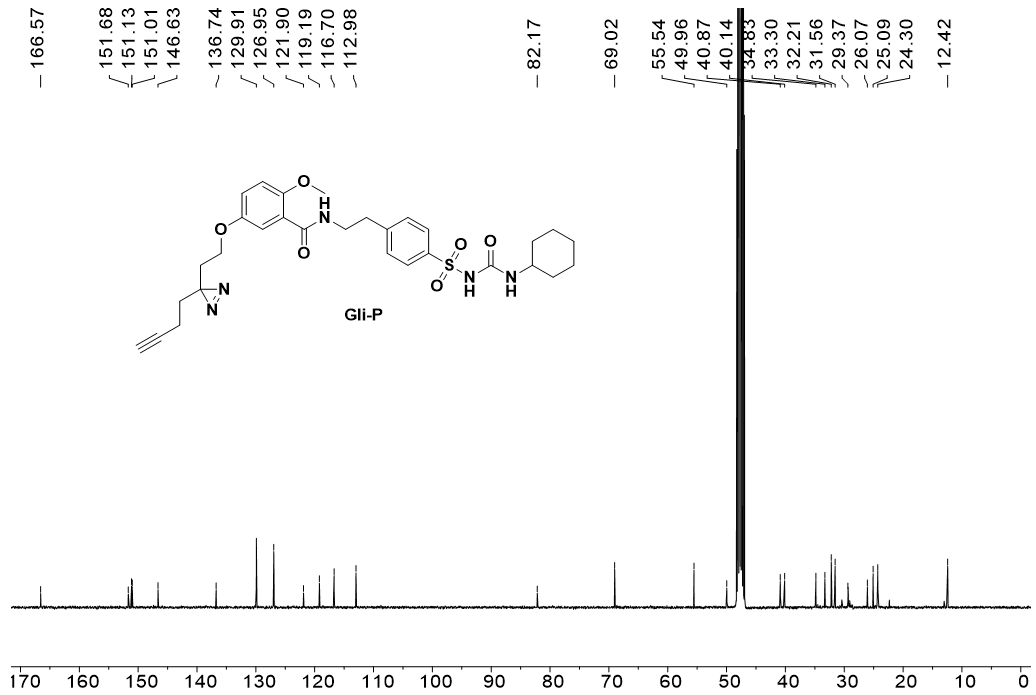


^13^C NMR spectrum of **Gli-P** in MeOD

**Figure. S19.**


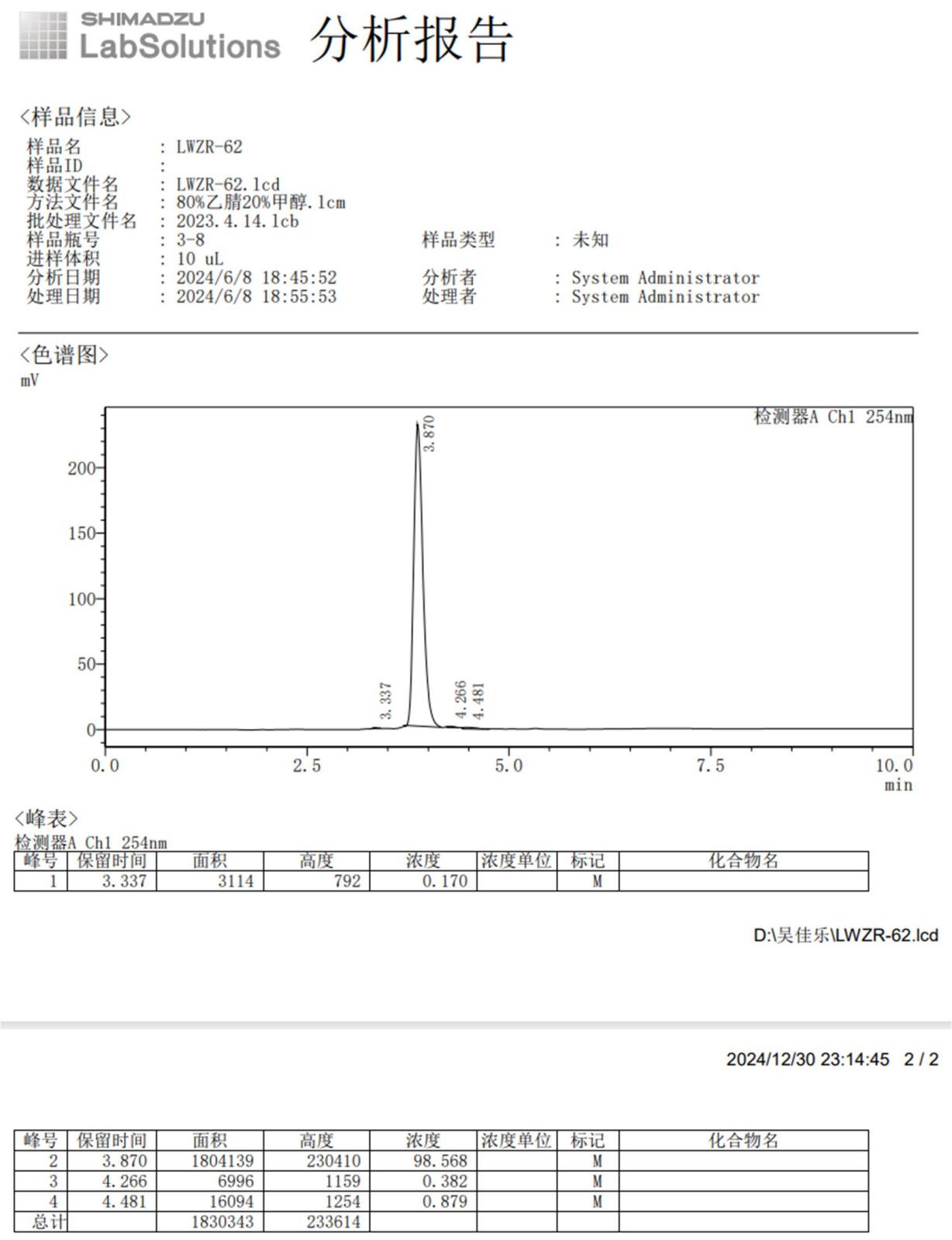


HPLC spectrum of **Gli-P**

**Figure. S20.**

**
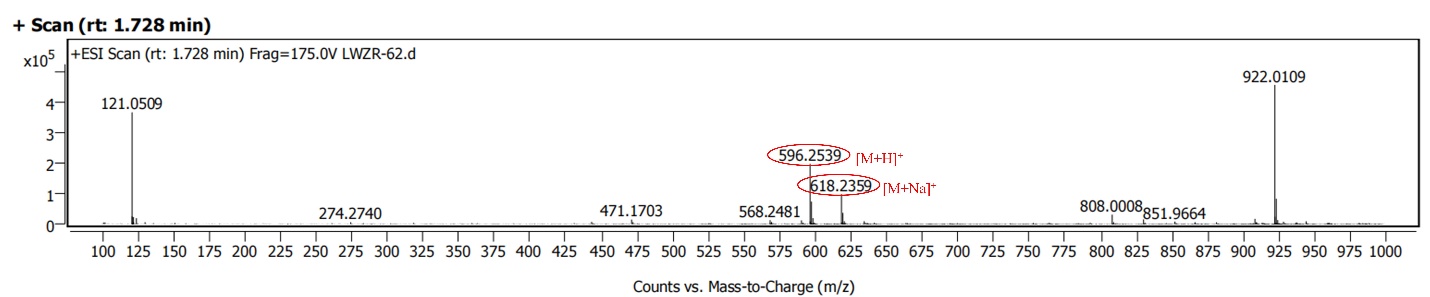
**

HRMS spectrum of **Gli-P**
