# Supplementary material for: Glibenclamide targets MDH2 to relieve aging phenotypes through metabolism-regulated epigenetic modification
Source: Signal Transduct Target Ther. 2025 Feb 17;10:67. doi: 10.1038/s41392-025-02157-3 (PMC11833132; doi:10.1038/s41392-025-02157-3)

Supplementary Data 2

Glibenclamide targets MDH2 to relieve aging phenotypes through metabolism-regulated epigenetic modification

Zhifan Mao^2†^, Wenwen Liu^1†^, Rong Zou^2†^, Ling Sun^1†^, Shuman Huang^2^, Lingyu Wu^1^, Liru Chen^1^, Jiale Wu^1^, Shijie Lu^2^, Zhouzhi Song^2^, Xie Li^2^, Yunyuan Huang^3^, Yong Rao^1^, Yi-You Huang^1^, Baoli Li^1*^, Zelan Hu^2*^, Jian Li^1,2,4*^

Correspondence to: jianli@ecust.edu.cn (J. L.); huzelan@ecust.edu.cn (Z. H.); baolili@hainanu.edu.cn (B. L.)

**Original films of Western blots**

Fig. 1b


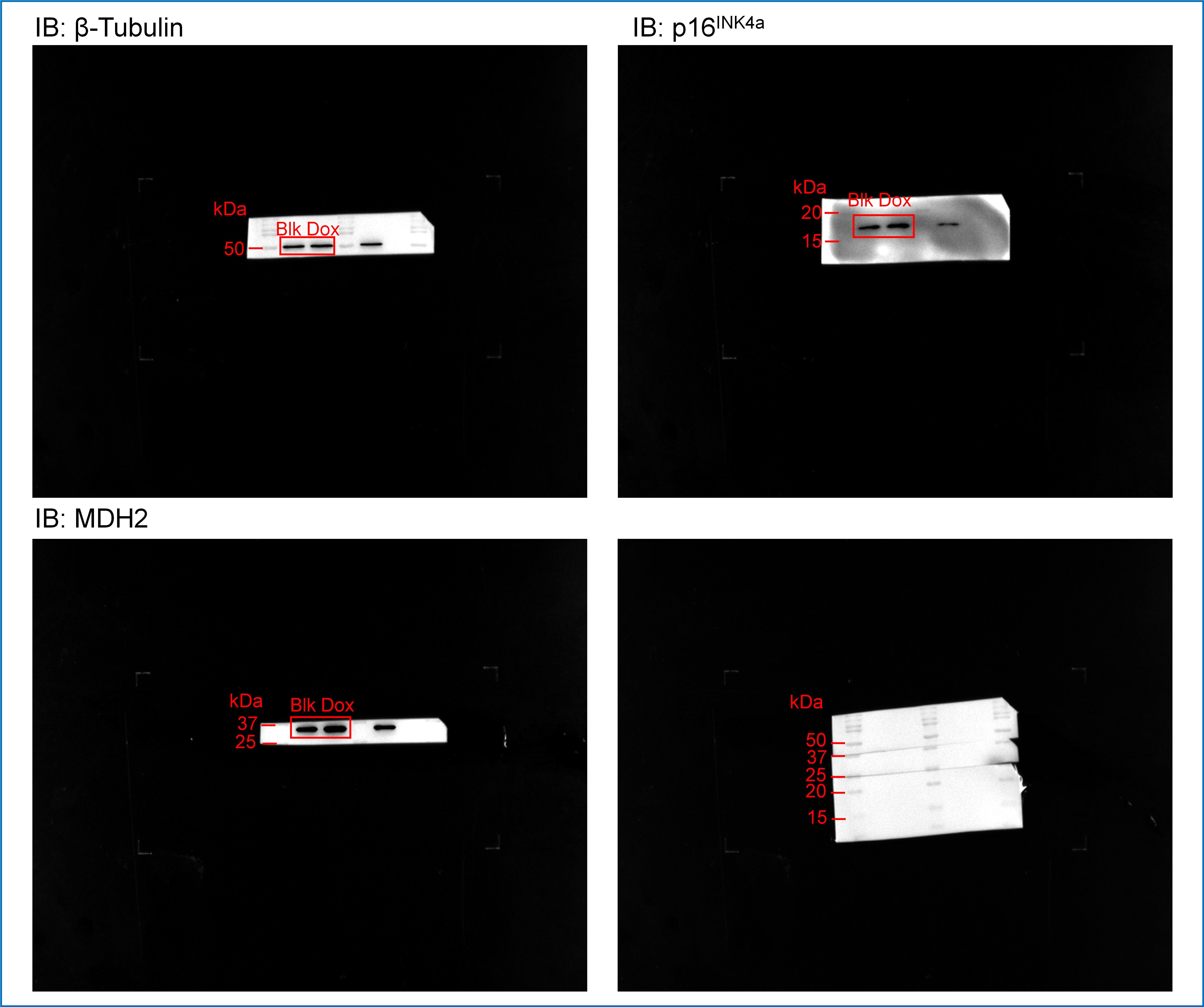


Fig. 1d


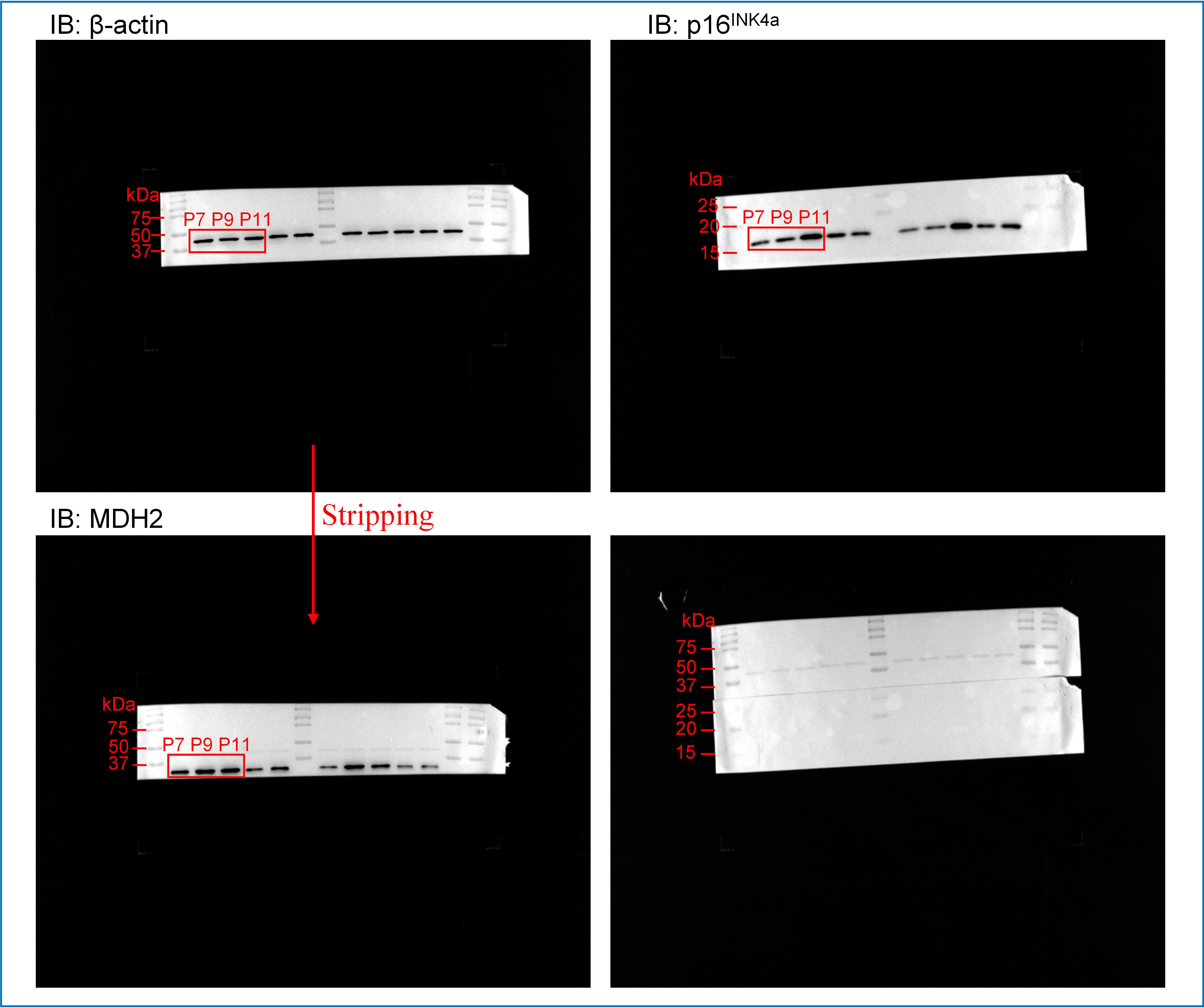


Fig. 1h


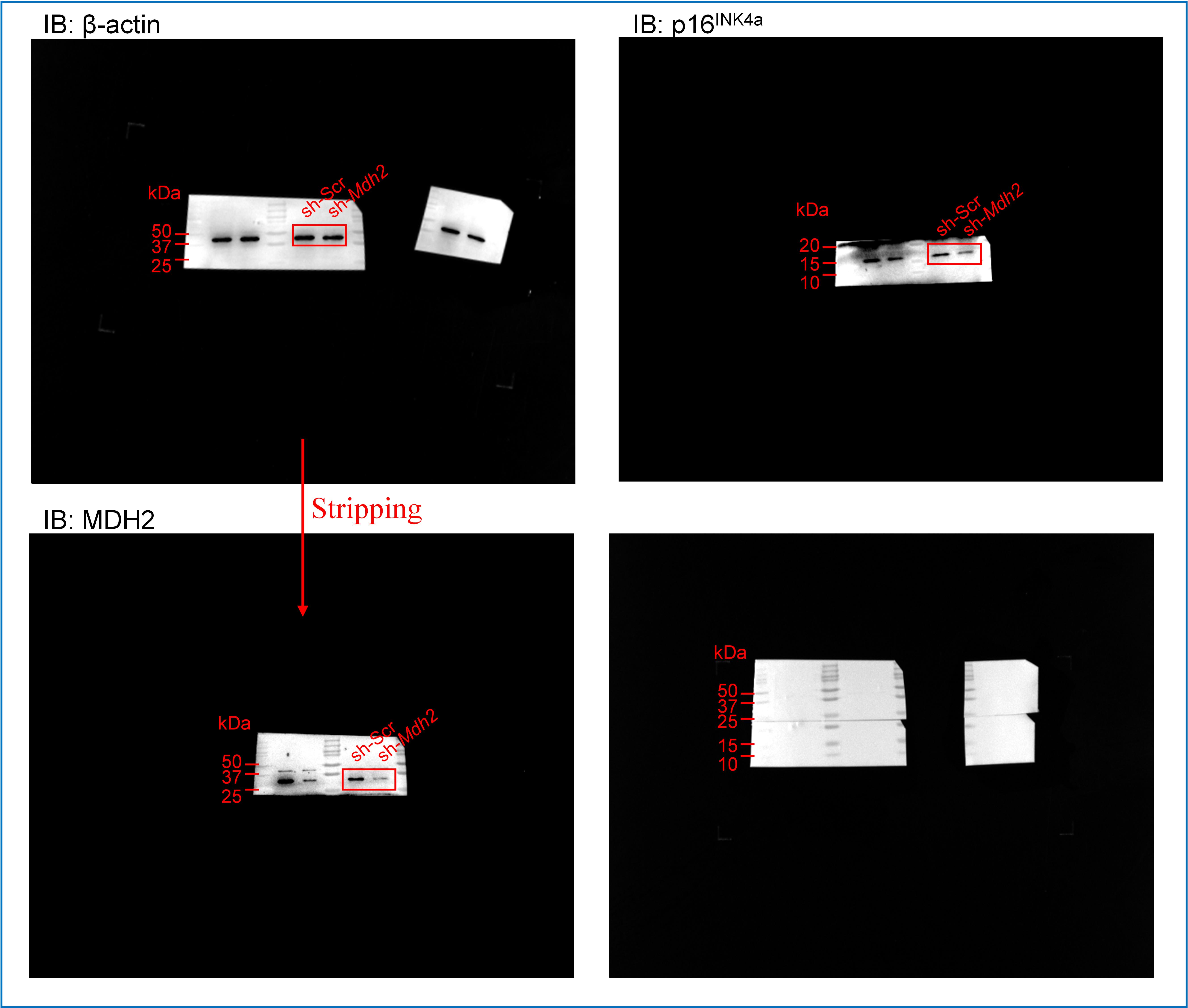


Fig. 1l


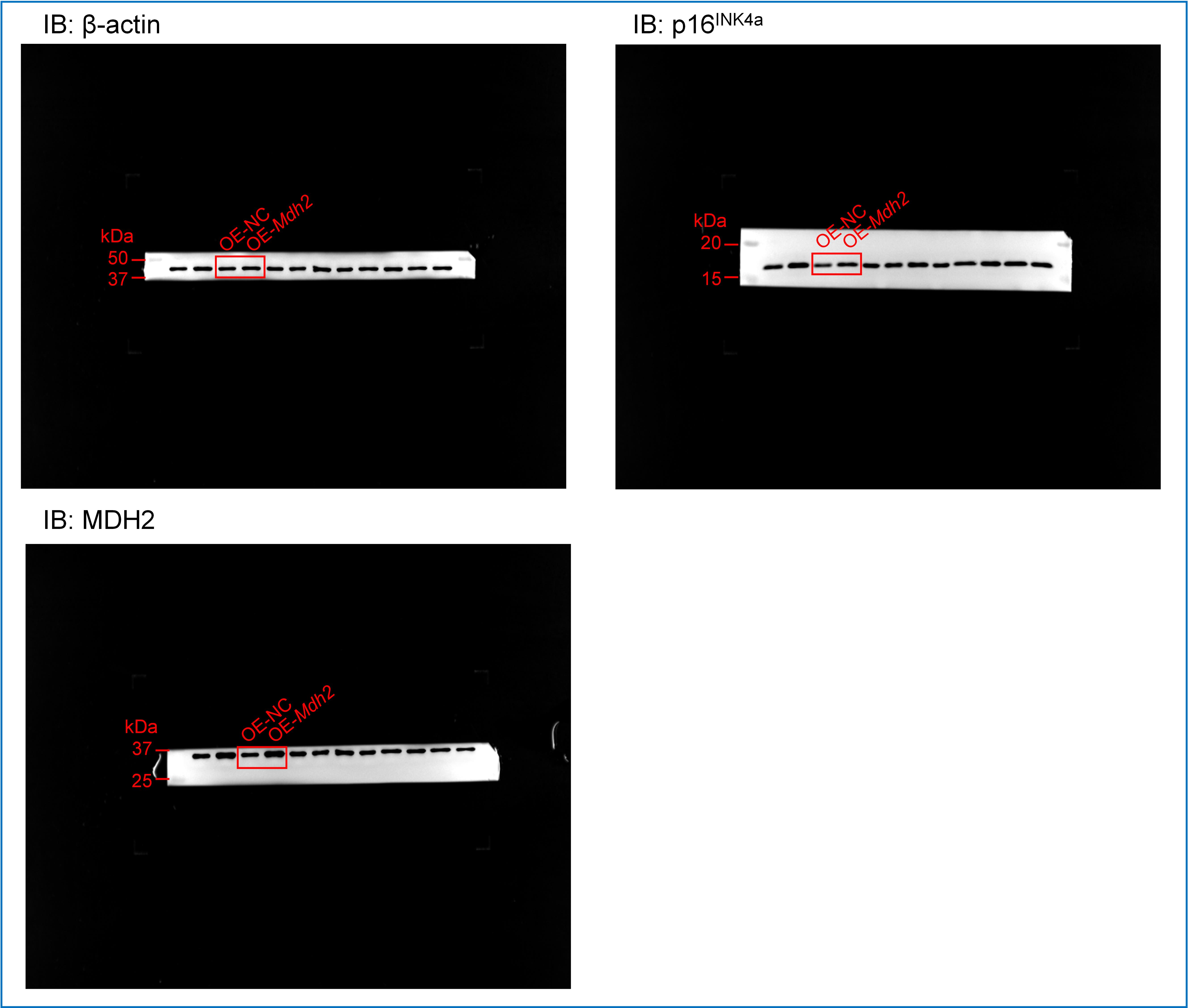


Fig. 2b


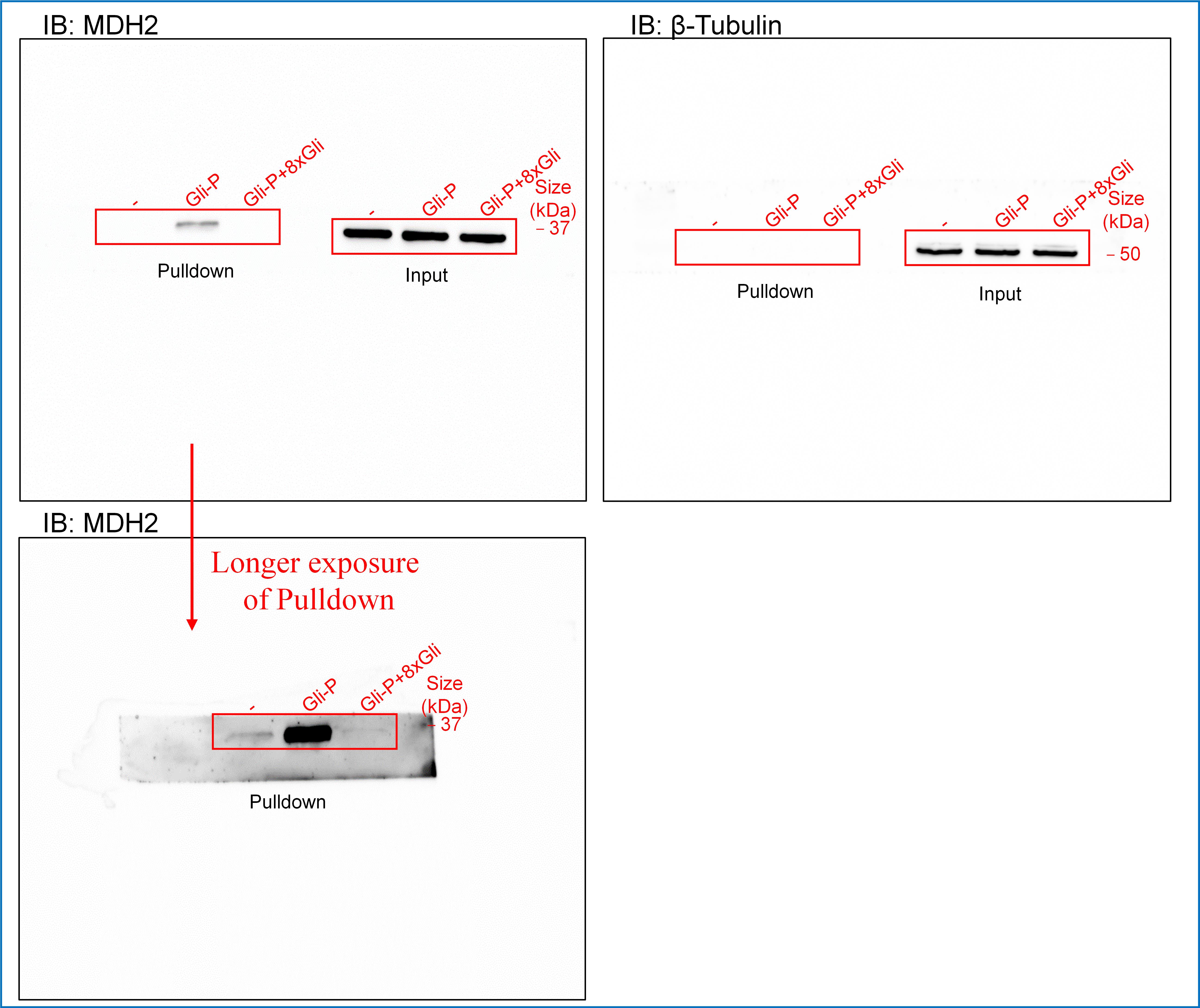


Fig. 2f


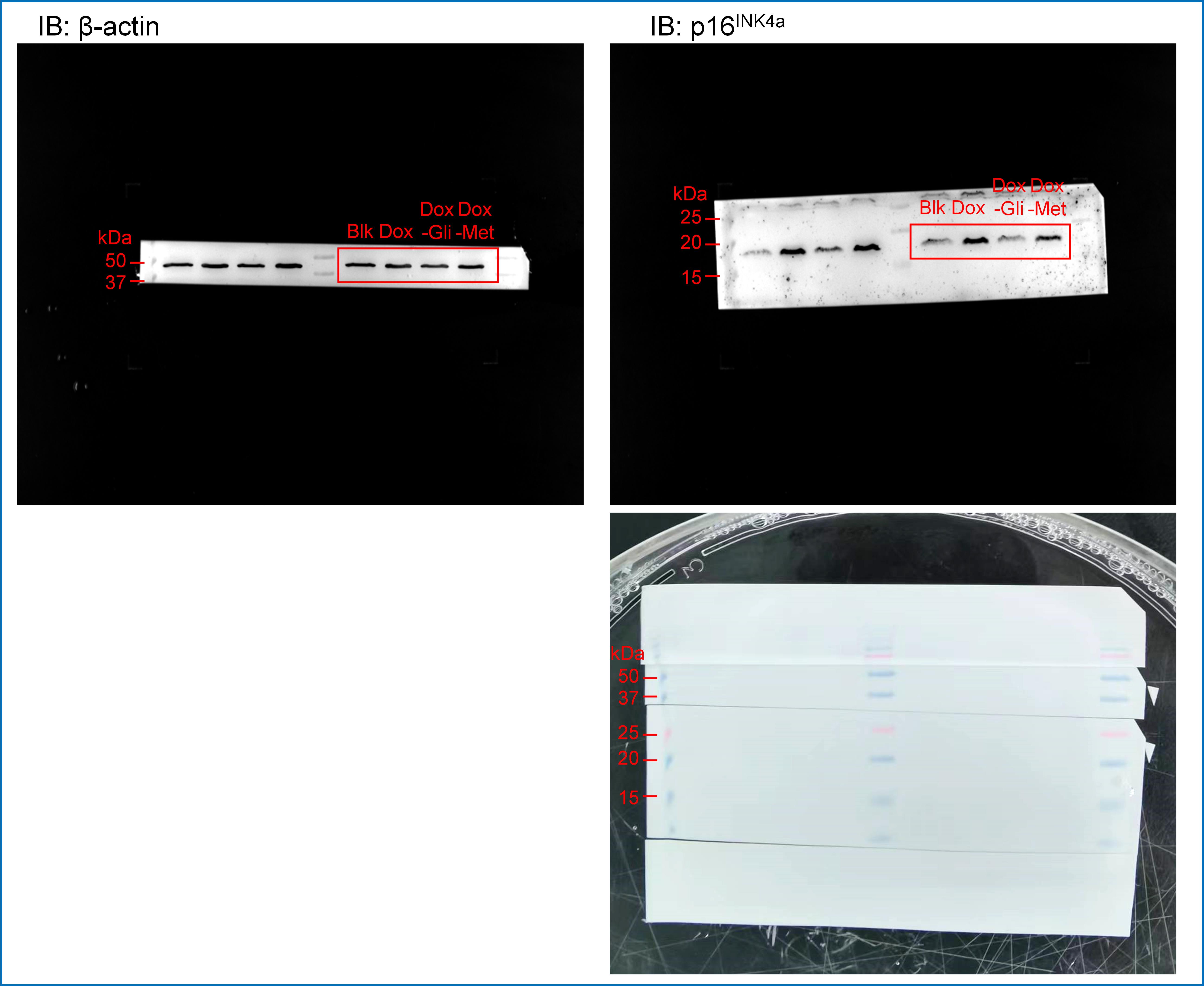


Fig. 2h


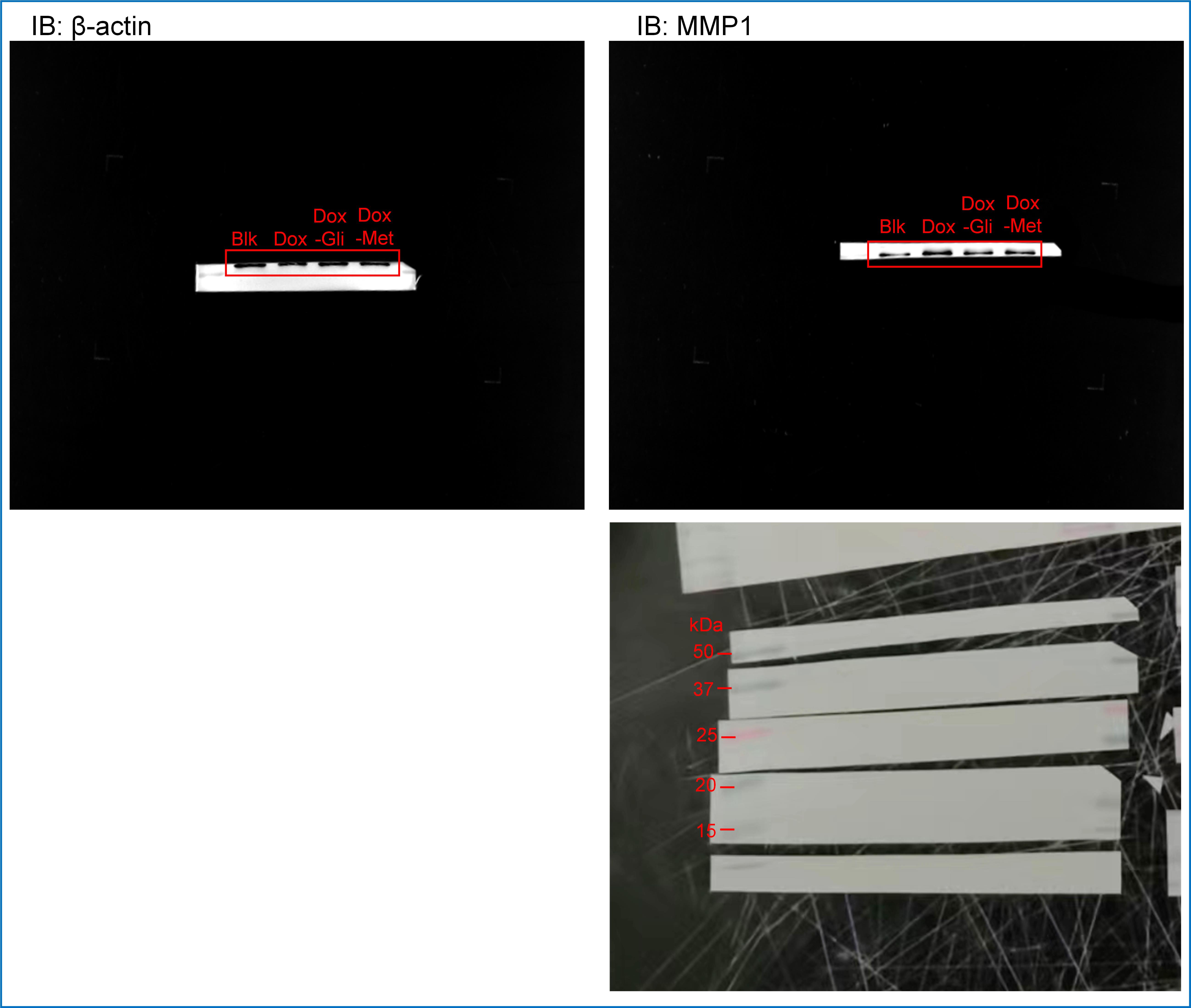


Fig. 2n


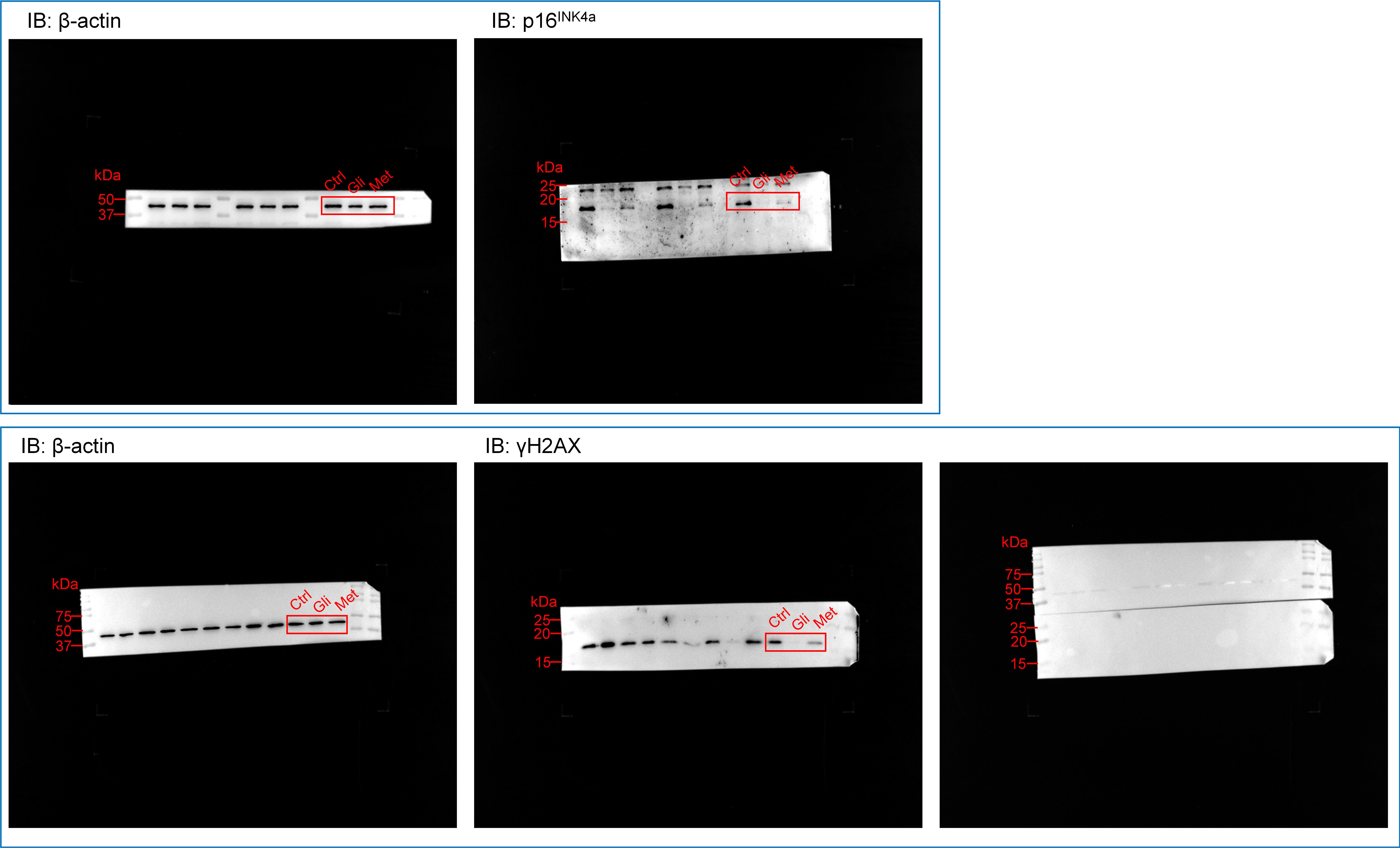


Fig. 3b


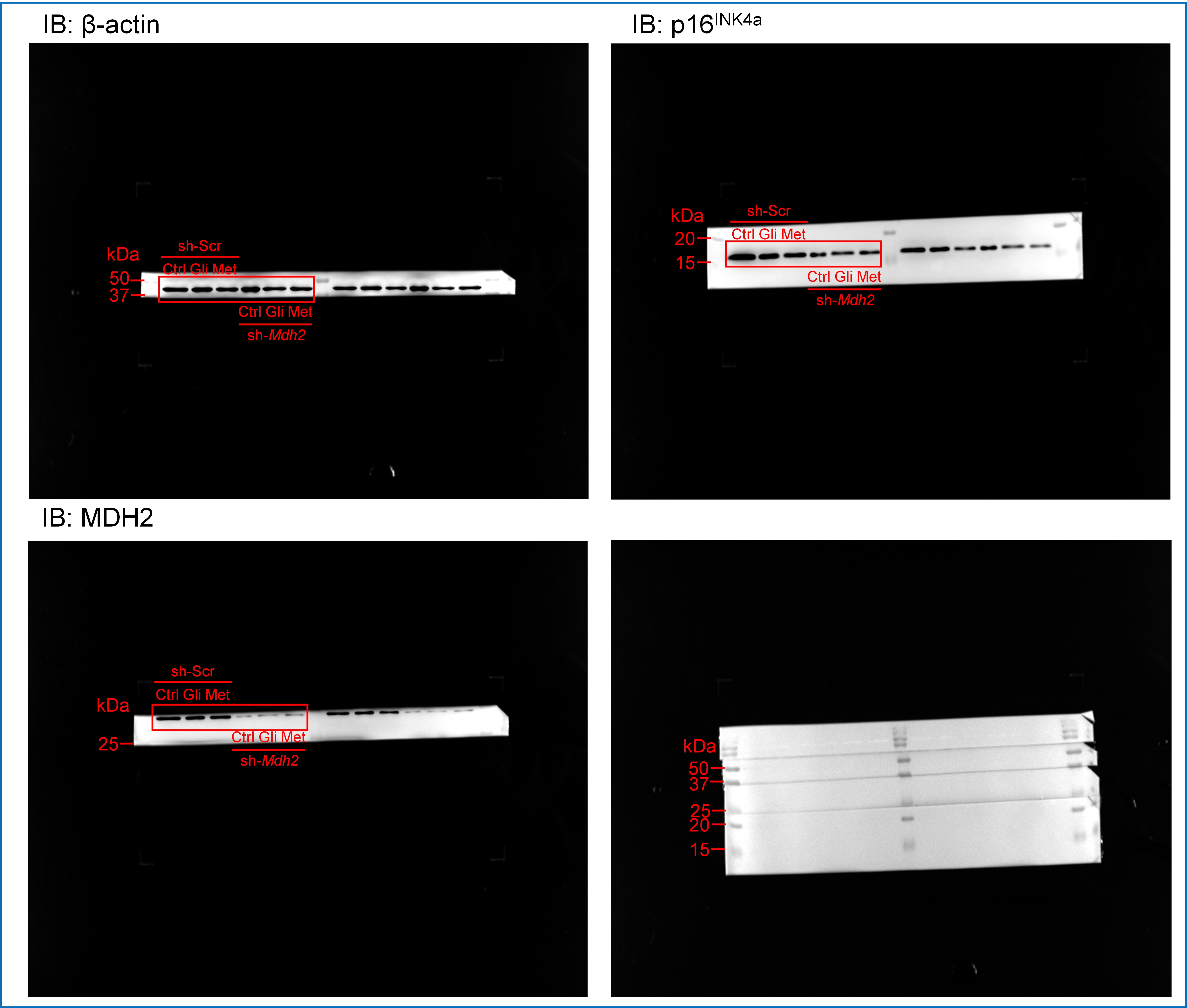


Fig. 3f


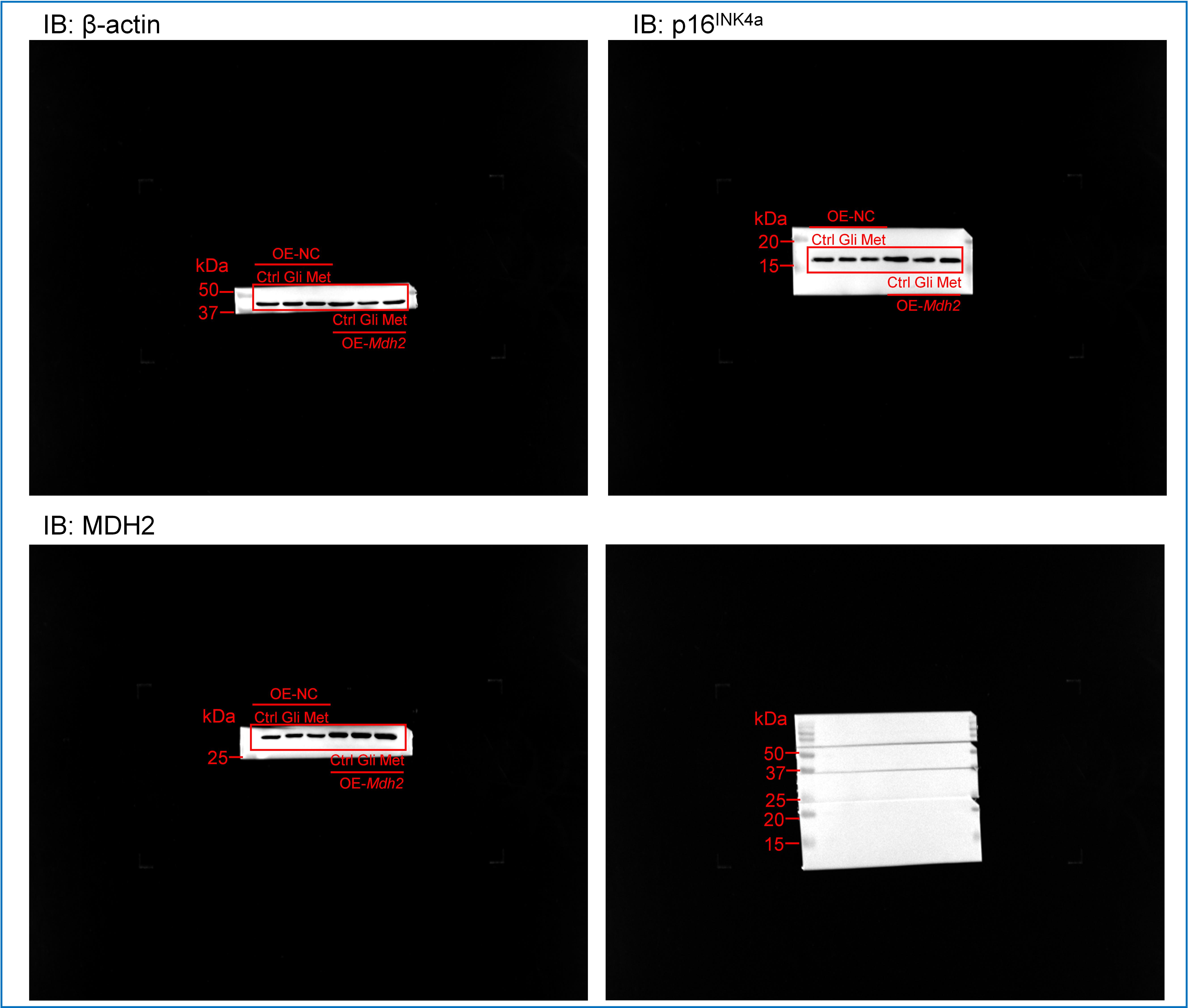


Fig. 5h


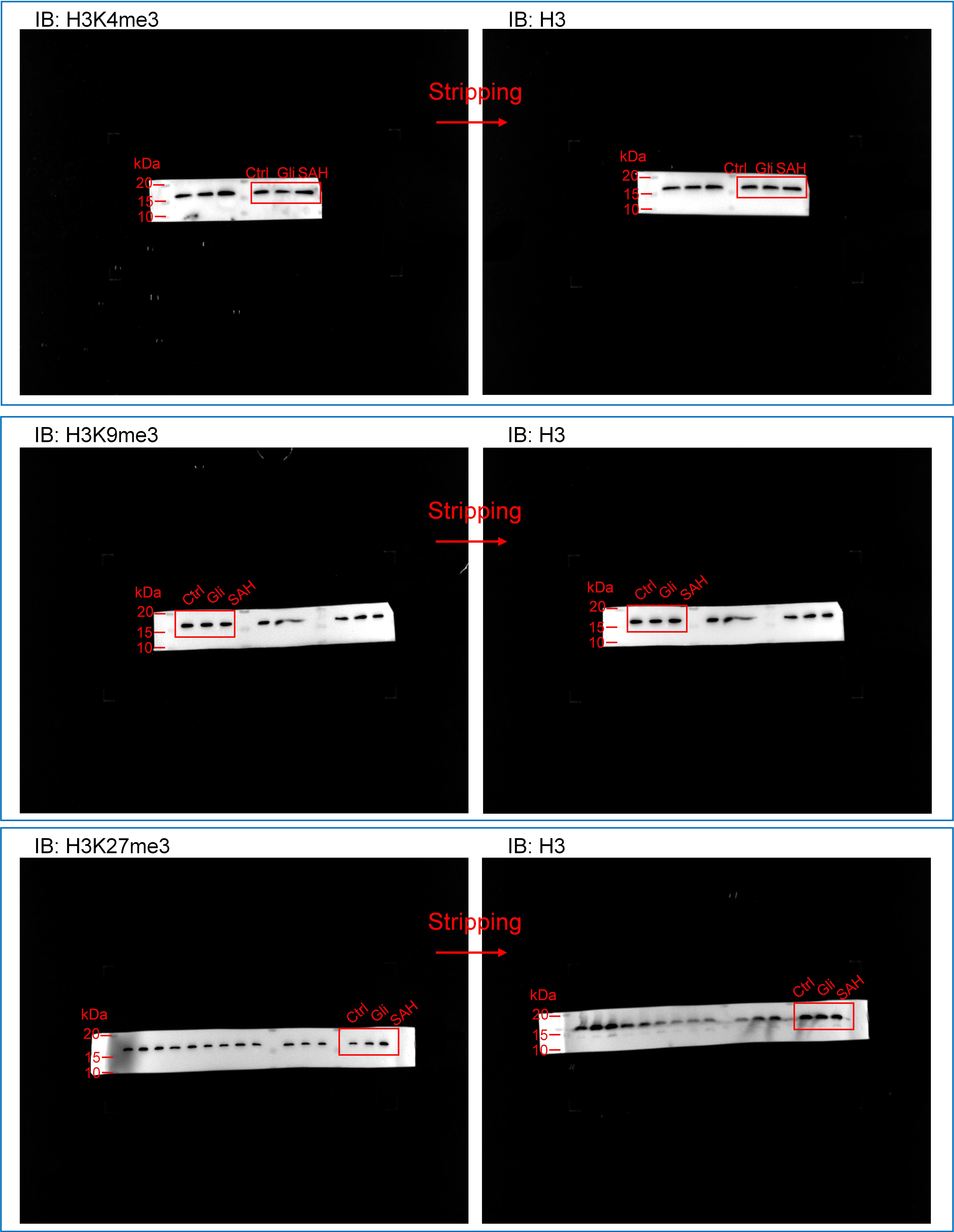


Fig. 5j


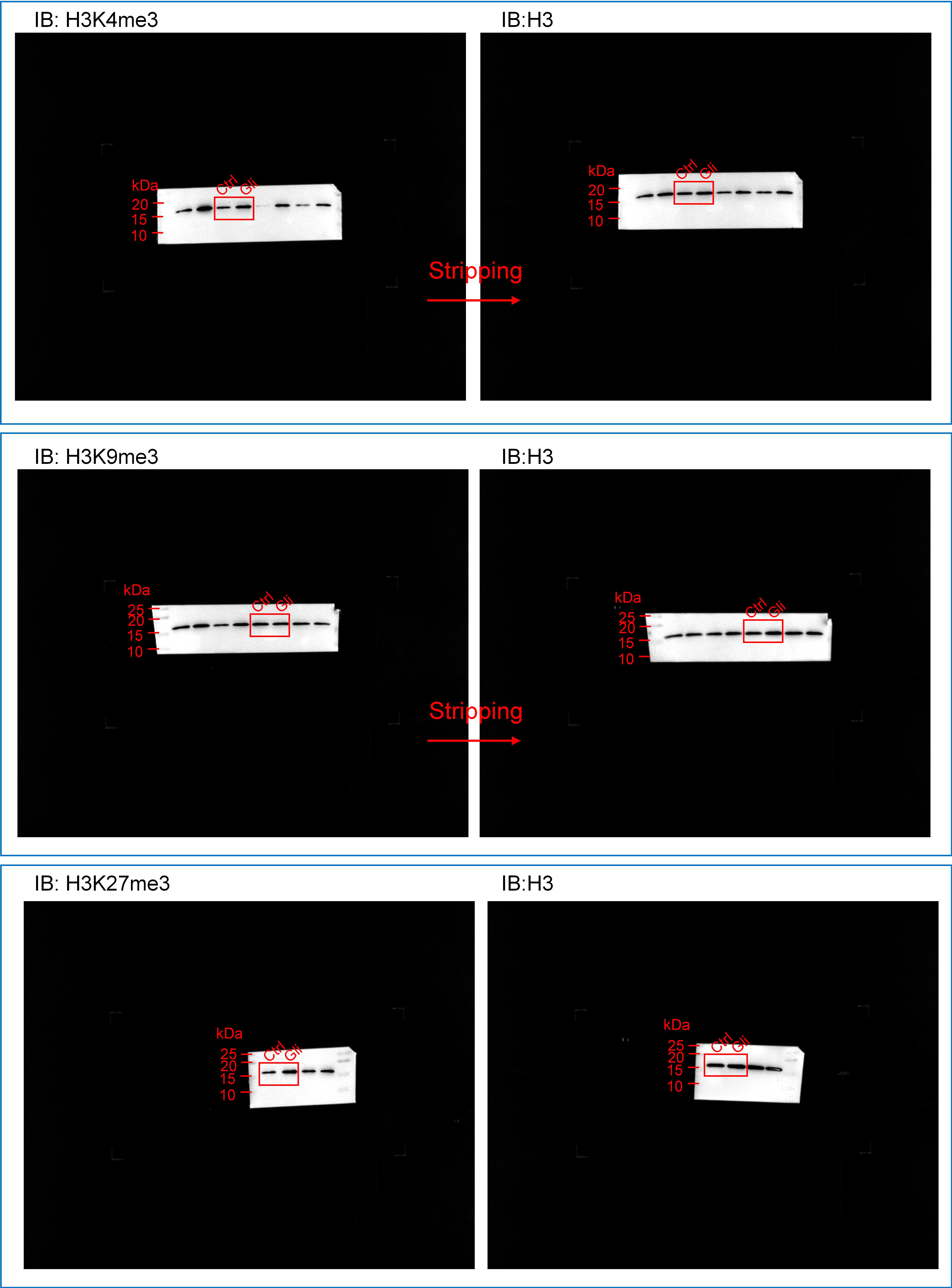


Fig. 6i


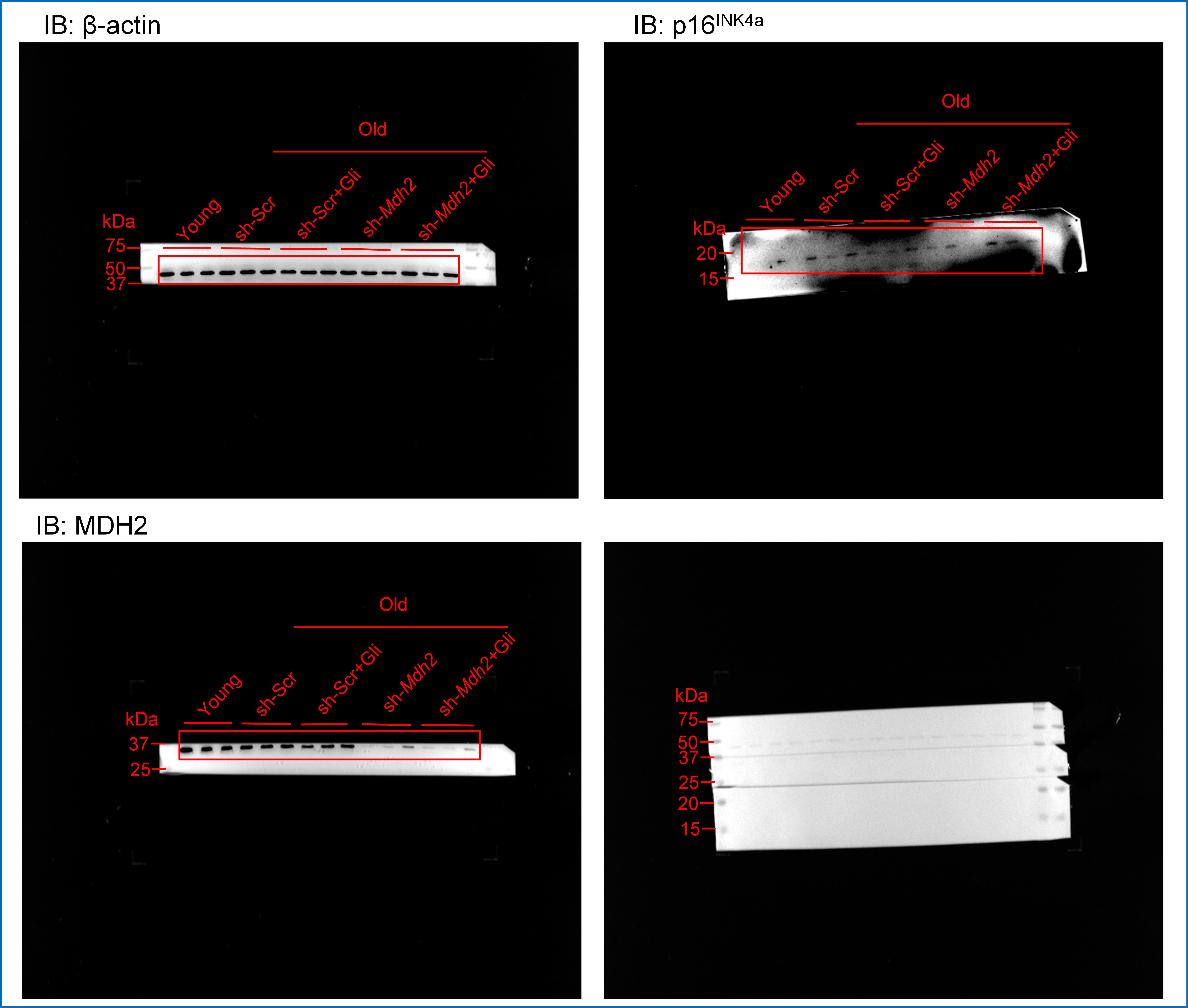


Fig. 6m


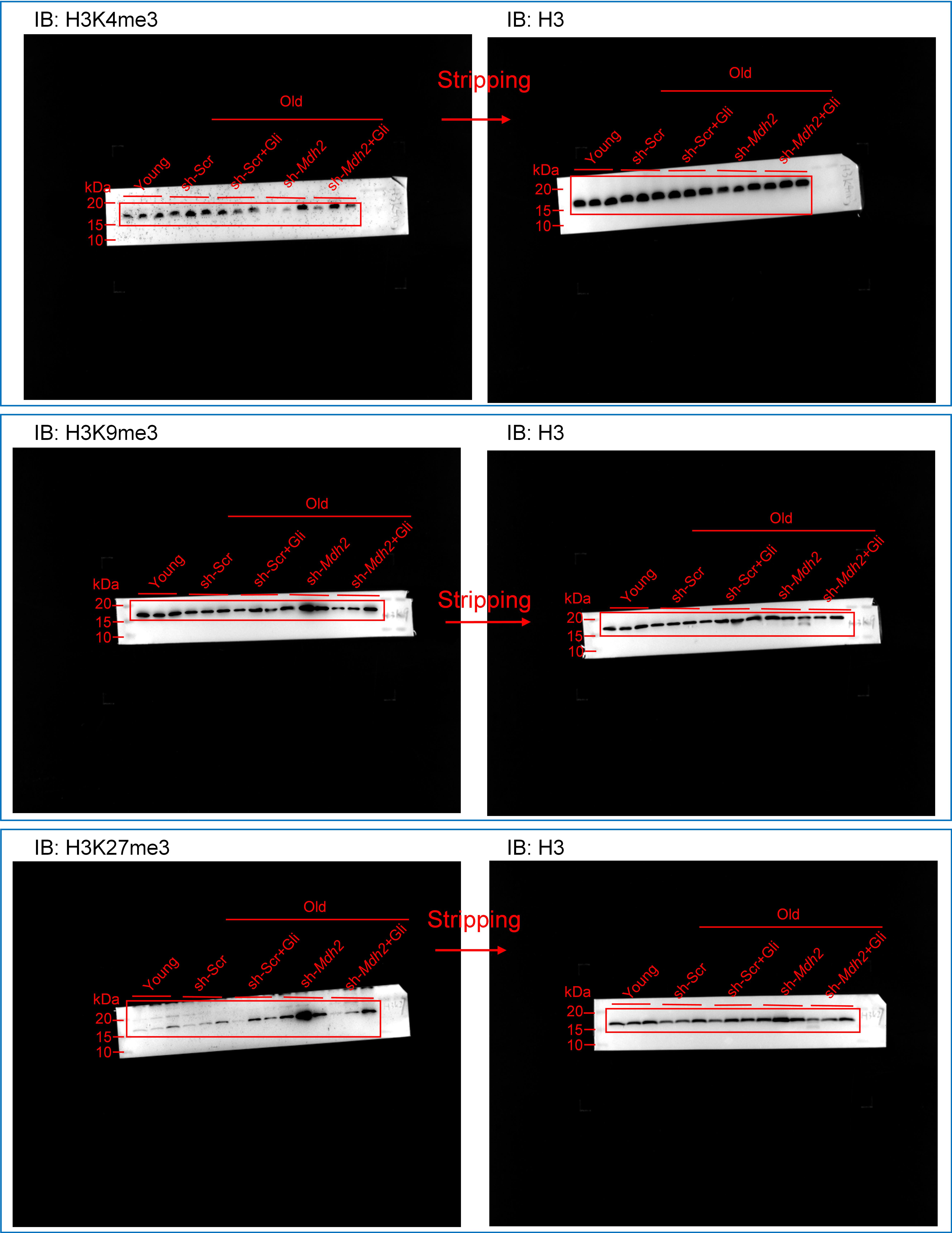

Supplement: Supplementary file 5 — Supplementary Data 2 [file 41392_2025_2157_MOESM5_ESM.docx]
